# Preoperative Biliary Drainage with Metal Stent Versus Early Surgery in Patients with Pancreatic Cancer: A Randomized Clinical Trial

**DOI:** 10.1245/s10434-026-19546-9

**Published:** 2026-04-08

**Authors:** Guido Costamagna, D. Nageshwar Reddy, Nam-hung Chia, Takao Itoi, Jacques Devière, Kit-fai Lee, G. V. Rao, Sergio Alfieri, Irene Lo, Kazuhiko Kasuya, Jean Closset, David L. Carr-Locke, Rohit Chandwani, Joyce Peetermans, Matthew Rousseau, James Lau

**Affiliations:** 1 Fondazione Policlinico Universitario Agostino Gemelli IRCCS (Università Cattolica del Sacro Cuore), Rome, Italy; 2 Asian Institute of Gastroenterology, Hyderabad, India; 3 Queen Elizabeth Hospital, Kowloon, Hong Kong, People’s Republic of China; 4 Tokyo Medical University, Tokyo, Japan; 5 Hôpital Erasme, Brussels, Belgium; 6 Prince of Wales Hospital, The Chinese University of Hong Kong, New Territories, Shatin, Hong Kong SAR People’s Republic of China; 7 CRMPG Gemelli Pancreatic Advanced Research Center, Rome, Italy; 8 Division of Gastroenterology and Hepatology, Weill Cornell Medicine, New York, NY USA; 9 Department of Surgery, Weill Cornell Medicine, New York, NY USA; 10 Department of Cell and Developmental Biology, Weill Cornell Medicine, New York, NY USA; 11 Sandra and Edward Meyer Cancer Center, Weill Cornell Medicine, New York, NY USA; 12 Endoscopy Division, Boston Scientific Corporation, Marlborough, MA USA

**Keywords:** Cholangiopancreatography, endoscopic retrograde, Drainage, Jaundice, obstructive, Pancreatic neoplasms/complications, Pancreatic neoplasms/surgery, Pancreaticoduodenectomy, Stents

## Abstract

**Background:**

Among patients with pancreatic cancer and biliary obstruction planned for pancreaticoduodenectomy, preoperative biliary drainage (PBD) may be considered during surgical delays. Higher complication rates have been reported for PBD using plastic stents versus early surgery. PBD with a self-expanding metal stent (SEMS) has not been compared with early surgery in a randomized controlled trial (RCT).

**Patients and Methods:**

We conducted a noninferiority RCT comparing PBD using a SEMS versus early surgery at 11 centers in 9 countries. We enrolled patients with resectable pancreatic or periampullary cancer and serum total bilirubin level ≥ 5.8 mg/dL, scheduled for primary resection. Primary endpoint was the proportion of patients reporting ≥ 1 serious adverse event (SAE) 120 days post-randomization. Secondary endpoints included rate of SEMS insertion, rate of curative-intent resection, and all-cause mortality.

**Results:**

Among 284 patients, 144 were randomized to PBD and 140 to early surgery. In the modified intention-to-treat primary endpoint analysis, ≥ 1 SAE(s) occurred in 29.0% (40/138) in the PBD group and 26.5% (36/136) in the early surgery group (between-group difference, 2.5%; one-sided upper 95% confidence limit, 11.7%; *P* = 0.011 for noninferiority). Among 144 PBD patients, 140 (97.2%) received a SEMS; 119 (82.6%) underwent surgery with curative intent. Among 140 early surgery patients, 14 (10.0%) underwent ERCP and drainage; 130 (92.9%) underwent surgery with curative intent in 115 (88.5%). During follow-up, 7.9% (11/138) in the PBD group and 8.0% (11/136) in the early surgery group died.

**Conclusion:**

Safety following PBD using SEMS was noninferior to early surgery for pancreatic or periampullary cancer. ClinicalTrials.gov, no. NCT01774019.

**Supplementary Information:**

The online version contains supplementary material available at 10.1245/s10434-026-19546-9.

Worldwide, nearly 450,000 new cases of pancreatic cancer are diagnosed each year.^1^ The incidence rate has increased from 5.0 to 5.7 per 100,000 population in the last two or three decades.^1^ An estimated 64,050 new cases were reported in the USA in 2023.^2^ Pancreatic cancer remains one of the deadliest cancers, with 5-year survival < 10% by most estimates.^1^ Surgery is the only potential cure.^3^

For the approximately 20% of patients with pancreatic cancer who are candidates for surgical resection, the role of preoperative biliary drainage (PBD) has been controversial.^4^ Adverse events associated with PBD led to questions regarding whether it should be considered. A Dutch multicenter randomized trial^5^ compared PBD using a plastic stent versus early surgery without PBD. The trial reported a high rate (46%) of PBD-related complications. Of these, 26% were cholangitis. This could be attributed to the small caliber of plastic stents. In a subsequent cohort study added to the Dutch trial, the authors concluded that use of a fully covered self-expanding metal stent (SEMS) should be preferred over plastic stents whenever PBD is indicated.^6^ Some^7^^–^^11^ but not all^12^^,^^13^ subsequent meta-analyses or clinical trials reported lower rates of reintervention with SEMS, but with a higher incidence of preoperative pancreatitis compared to plastic stents.^7^^,^^10^^,^^11^ Clinical practice guidelines recommend against routine PBD but acknowledge it may be considered in patients with cholangitis, severe symptomatic jaundice, delayed surgery, or before neoadjuvant therapy.^14^^–^^23^ This issue continues to be clinically relevant, as shown by an analysis of data from the National Surgical Quality Improvement Program (NSQIP), which documented an endoscopic stent in over half of patients undergoing pancreatoduodenectomy 2014–2017 who did not have neoadjuvant therapy.^24^

A randomized trial that compares PBD using SEMS versus early surgery is not available in the literature.^25^ In patients with planned curative-intent surgery to treat pancreatic or periampullary cancer, we conducted a multicenter, randomized, noninferiority trial to compare outcomes in patients who underwent PBD using SEMS versus those who received surgery without preoperative drainage. We hypothesized that PBD using SEMS would not negatively impact patient outcomes.

## Patients and Methods

### Trial Design

This study was a multicenter, noninferiority randomized clinical trial. The study was approved by the individual institutional review boards and ethics committees of all participating sites and registered in the ClinicalTrials.gov database (NCT01774019). Study participation began in February 2013 and ended in December 2021. All participants provided written informed consent before randomization. One gastroenterologist (D.C.L.) and a pancreaticobiliary surgeon (R.C.) independently adjudicated all SAEs and categorized postoperative complications according to the Clavien–Dindo classification. The study sponsor had a role in study design, trial execution, central database building and maintenance, data monitoring, statistical analysis, interpretation, and writing of the report, in collaboration with the academic investigators. The authors had access to the study data and have reviewed and approved the final manuscript.

Patients were followed until 120 days after randomization or 30 days after surgery, whichever came last, up to 150 days post-randomization. Patients who did not proceed to surgery, who transitioned to palliative care, or who were lost to follow-up continued to have safety follow-up until dropout or end of the study and were included in the primary endpoint analysis.

### Participants

The clinical trial was conducted at 11 centers in 9 countries: Australia, Belgium, China, France, Hong Kong, India, Italy, Japan, and the USA. The site investigators screened patients for study eligibility and enrolled participants. Inclusion criteria were: age 18 years or older with a diagnosis of distal biliary obstruction consistent with pancreatic cancer, distal bile duct cholangiocarcinoma and other periampullary cancers, deemed resectable on pancreatic protocol computed tomography (CT) or magnetic resonance imaging (MRI); serum total bilirubin level ≥ 100 μmol/L (5.8 mg/dL); proximal end of the tumor at least 2 cm from hilum; surgical candidate per pancreatobiliary surgeon after multidisciplinary discussion; planned for surgery with curative intent within 4 weeks of enrollment in the study. We excluded patients with benign biliary strictures, with surgically altered anatomy, undergoing neoadjuvant chemotherapy not considered for curative surgery, with previous biliary drainage by endoscopic retrograde cholangiopancreatography (ERCP) or percutaneous routes, or enrolled in another clinical trial within 90 days. Race/ethnicity data were not collected because of ethics policies at some sites that required data to be disaggregated by racial or ethnic origin.^26^

### Endoscopic Interventions

Patients assigned to the PBD group underwent ERCP and insertion of an uncovered or covered SEMS (WallFlex Biliary RX™, Boston Scientific Corporation, Marlborough, MA; 8 or 10 mm diameter, each with length of 40, 60, or 80 mm, except for the 8-mm-diameter × 40-mm-length, fully covered version). An uncovered SEMS was used in strictures involving the cystic duct confluence and in bile ducts with a low cystic duct confluence. Patients underwent resection after resolution of jaundice (defined as serum bilirubin below 5.8 mg/dL), and within 4 weeks of stenting.

### Outcomes

#### Primary Endpoint

The primary endpoint was the rate of ≥ 1 adjudicated serious adverse event (SAE),^27^ including preoperative and operative SAEs reported up to day 120 post-randomization or day 150 where needed per protocol. The study protocol included a “protocol-specified” list of definitions of complications related to PBD or surgical treatment from a previous RCT using plastic stents.^5^

#### Secondary Endpoints

The secondary endpoints included: (1) technical success of ERCP and SEMS insertion and associated complications; (2) the rate of surgery in either group, and complications following surgery; we further categorized surgery-related complications using the Clavien–Dindo classification;^28^^,^^29^ (3) all-cause mortality within 120 days. In the subgroup of patients with baseline serum bilirubin > 14.6 mg/dL (> 250 μmol/L), we compared rates of postoperative complications in either group.

### Statistical Analysis

#### Sample Size Calculations

In a pooled analysis of eight studies,^30^^–^^37^ the rate of complications of early surgery without PBD was 24.2% (95% confidence interval (CI) 13.2–37.2%). We used the upper limit of the 95% CI, i.e., 37.2%, from this analysis for sample size calculations. With an exact test with one-sided 5% with type 1 error (noninferiority margin of 15%), 264 subjects would provide 80% power to reject the null hypothesis that the SAE rate in the PBD group was inferior to that in the early surgery group. Allowing for 10% attrition, we intended to enroll 294 patients.

#### Sequence Generation, Implementation, and Blinding

A biostatistician generated a randomization sequence in which patients were randomized in blocks of 4 in a 1:1 ratio, stratified by site using a centralized online database system. Participants, and personnel giving the interventions, assessing outcomes or analyzing the data were not masked to group assignments.

#### Statistical Methods

To assess the primary endpoint, we calculated the one-sided 95% confidence interval (CI) to the between-group difference using an exact CI. We would accept noninferiority if the upper bound of the 95% CI was less than 15% or a *P* value below 0.05. For discrete outcomes, a 95% exact CI of the difference between groups was calculated. Kaplan-Meier methods were used to estimate time to occurrences of SAEs and overall survival between groups. The primary endpoint power calculation, upper CI, and *P* value were calculated using StatXact version 12; all other analyses were performed using SAS version 9.4.

## Results

### Patients: Numbers Randomized and Analyzed

**Fig. 1:**
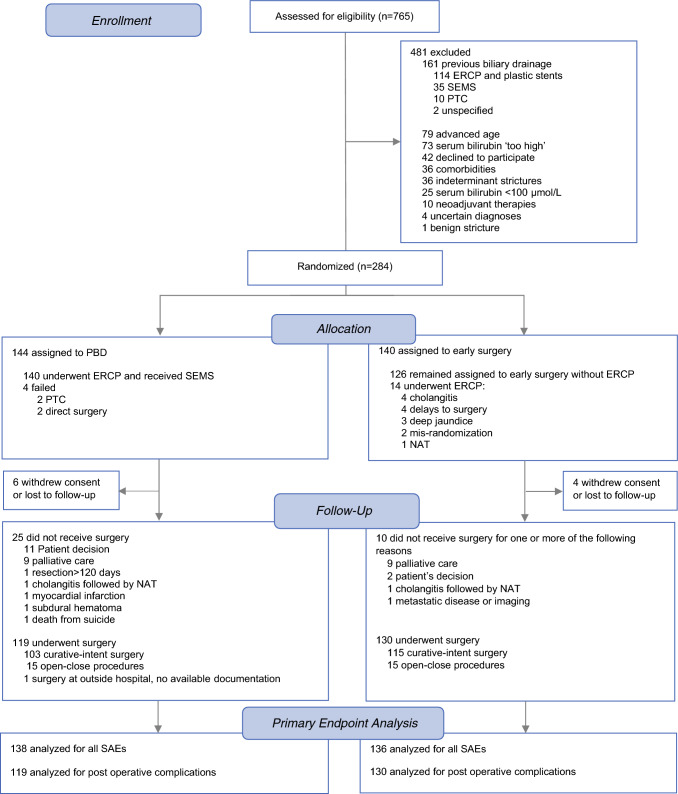
Patient flow diagram for modified intention to treat primary endpoint analysis

Between 11 February 2013 and 12 August 2021, 284 patients were enrolled and randomized; 144 to PBD and 140 to early surgery (Fig. 1). Six and four patients from the PBD and early surgery group, respectively, were lost to follow-up or withdrew consent; 138 and 136 patients entered a modified intention-to-treat analysis for the primary endpoint.

### Baseline Characteristics

The mean age of the entire cohort was 63.4 years, and the mean Karnofsky score was 90.5. The mean bilirubin at baseline was 14.0 and 12.9 mg/dL in the PBD and early surgery group, respectively. At baseline, 53 (36.8%) and 43 (30.9%) had serum bilirubin > 14.6 mg/dL. The tumor size was 2.8 cm and 2.6 cm in either group. The tumors were located at the head of pancreas (70.8% versus 61.4%), periampullary (20.8% versus 24.3%), and the distal common bile duct (8.3% versus 11.4%). Few tumors were located at the uncinate process (1.4%), isthmus (0.7%), duodenum (0.7%), or were multifocal (0.7%) (Table 1). The tumors were staged from 1A to 3 based on the American Joint Committee on Cancer Stage (Table 1). The histological subtypes of these tumors are listed in Supplementary Table 1.

**Table 1:** Baseline characteristics of the patients and their tumors

Characteristic	Preoperative biliary drainage (*N *= 144)	Early surgery (*N *= 140)
Mean age (SD), years	61.9 (11.5)	64.9 (11.7)
Male, *n* (%)	80 (55.6)	81 (57.9)
Mean height (SD), cm	163.3 (9.1)	161.8 (8.7)
Mean weight (SD), kg	65.0 (12.8)	63.2 (12.7)
BMI (SD), kg/m^2^	24.3 (3.9)	24.1 (3.9)
Mean Karnofsky score (SD)	91.3 (7.3)	89.6 (9.1)
Mean total serum bilirubin (SD), mg/dL	14.0 (6.3)*	12.9 (5.0)*
Bilirubin > 14.6 mg/dL, *n* (%)*	53 (36.8)	43 (30.9% of 139)
Mean tumor size on imaging (SD), cm	2.8 (3.4)	2.6 (2.3)^†^
*Tumor location on imaging*, *n* (%)		
Head	102 (70.8)	86 (61.4)
Periampullary	30 (20.8)	34 (24.3)
Distal common bile duct	12 (8.3)	16 (11.4)
Uncinate process	0	2 (1.4)
Isthmus	0	1 (0.7)
Duodenum	0	1 (0.7)
Multifocal	0	1 (0.7)
*Tumor type*, *n* (%)^‡^		
Periampullary	32 (48.5)	32 (59.3)
Exocrine pancreas	29 (43.9)	20 (37.0)
Extrahepatic bile ducts	3 (4.5)	3 (3.7)
Pancreatic endocrine	2 (3.0)	0
*AJCC tumor stage*, *n* (%)		
0	0	0
1A	17 (11.8)	17 (12.1)
1B	26 (18.1)	29 (20.7)
2A	12 (8.3)	15 (10.7)
2B	28 (19.4)	21 (15.0)
3	2 (1.4)	2 (1.4)
4	0	0
Unknown	59 (41.0)	56 (40.0)

AJCC, American Joint Committee on Cancer; BMI, body mass index

^*^Six patients (three in each group) had baseline bilirubin levels < 5.8 mg/dL, with values of 1.6 (with alkaline phosphatase of 1387 IU/L), 3.9, 4.1, 4.2, 4.3, and 5.01 mg/dL. For the dichotomous serum bilirubin threshold, 14.6 mg/dL = 250 μmol/L

^†^From 135 patients

^‡^From 120 patients: 66 preoperative biliary drainage, 54 early surgery

### Primary Endpoint: All SAEs

**Fig. 2:**
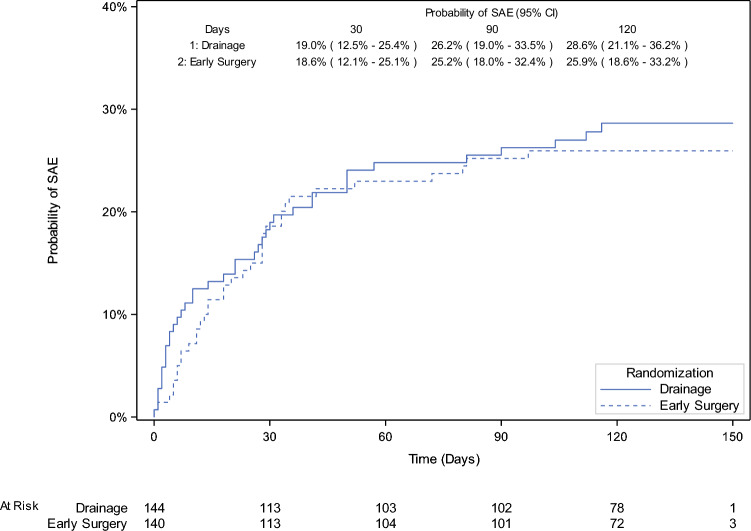
Kaplan–Meier estimate of the cumulative incidence of any serious adverse event (SAE) to 120 days post-randomization

The proportion of patients with at least one adjudicated SAE during the study period was 29.0% (40/138) in the PBD and 26.5% (36/136) in the early surgery group (between-group difference, 2.5%; one-sided upper limit of 95% CI, 11.7%; *P *= 0.011 for noninferiority) (Table 2; Supplementary Table 2). The upper confidence limit for the difference was within the noninferiority margin of 15%. The Kaplan–Meier analysis showed that the 120-day cumulative incidence of any SAE was not significantly different between groups, (hazard ratio (HR) 1.1 (95% CI 0.7–1.8) (Fig. 2).

**Table 2:** Outcomes after preoperative biliary drainage or early surgery

	Preoperative biliary drainage (*N *= 144)	Early surgery (*N *= 140)	Between-group difference, percentage points
*Primary endpoint*			
Proportion of patients with at least one SAE, *n*/*N* (%)	40/138 (29.0)	36/136 (26.5)	2.5; upper one-sided 95% CL, 11.7%; *P* = 0.011*
Proportion of patients with at least one protocol-specified SAE	32/138 (23.2)	35/136 (25.0)	− 1.8 (− 12.2 to 8.6)^†^
Before surgery	21/138 (15.2)	7/136 (5.1)	10.1 (2.3 to 17.9)^†^
Operative and postoperative	23/119 (19.3)	30/130 (23.1)	− 3.8 (− 14.0 to 6.7)^†^
*Secondary endpoints*			
Rate of surgery, *n*/*N* (%)	103/118 (87.3)^‡^	115/130 (88.5)^‡^	− 1.2 (− 9.8 to 7.3)^‡^
120-Day all-cause mortality, *n*/*N* (%)	11/144 (7.6)	11/140 (7.9)	− 0.3 (− 7.0 to 6.4)^†^
Before surgery	7/144 (4.9)	1/140 (0.7)	4.2 (0.3–9.2)^†^
After surgery	4/119 (3.4)^‡^	10/130 (7.7)^‡^	− 4.3 (− 10.7 to 1.7)^†^
After curative resection	1/119 (0.8)^‡^	7/130 (5.4)^‡^	− 4.5 (− 10.0 to 0.02)^†^
After failed resection	3/119 (2.5)^‡^	3/130 (2.3)^‡^	0.2 (− 4.4 to 5.2)^†^
*Clavien–Dindo classification of surgical complications* (*n*/*N*) (%)			
II	12/103 (11.7)	13/115 (11.3)	
IIIa	6/103 (5.8)	5/115 (4.3)	
IIIb	2/103 (1.9)	1/115 (0.9)	
Iva	3/103 (2.9)	10/115 (8.7)	
V	1/103 (1.0)	7/115 (6.1)	

Data are *n*/*N* (%), upper one-sided 95% confidence limit (CL) for the primary endpoint, or % (95% confidence interval) for other endpoints

^*^Intention-to-treat analysis.* P* < 0.05 suggests that preoperative biliary drainage using SEMS is noninferior to early surgery

^†^Exact 95% confidence intervals. Includes one patient who had both an intraoperative and a postoperative SAE

^‡^Excludes 25 patients in the drainage group and 10 patients in the early surgery group who did not undergo attempted curative-intent surgery, plus 1 patient in the drainage group who had missing data for this outcome

^§^In the early surgery group, cholangitis was not related to PBD

### Technical Success of ERCP with SEMS Insertion and Associated Complications and Biliary Reinterventions

In the PBD group, 144 patients underwent ERCP. Stent placement was technically successful in 140 patients (97.2%, 97 fully covered and 43 uncovered SEMS). There were four failed ERCPs; two underwent percutaneous biliary drainage, and two received surgery without preoperative drainage. Further ERCPs were required in nine instances among seven patients for the following indications: cholangitis (two patients), cholangitis once and hemorrhage twice in one patient, migration (one), persistent jaundice (one), pancreatitis (one), and stent occlusion by a bile duct stone (one).

In the early surgery group, 14 patients had ERCP or other biliary interventions for the management of cholangitis (4 patients), prolonged waiting time to surgery (4), deep jaundice (3), error in randomization (enveloped assignments were used instead of the online database system) (2), and chemotherapy for locally advanced tumor (1) (Supplementary Table 3). One of the patients with cholangitis underwent a percutaneous transhepatic drain after a failed ERCP because of failed access to the papilla with malignant duodenal obstruction.

### Rate of Surgery and Curative-Intent Resections

In the PBD group, 25 patients did not receive surgery because of patients’ decisions and comorbid illnesses (11 patients, 44.0% of 25), transition to palliative care (9, 36.0% of 25), resection after 120 days post-randomization (1, 4.0% of 25), cholangitis followed by palliative chemotherapy (1), myocardial infarction (1), subdural hematoma (1), and suicide (1) (Supplementary Table 4).

In the early surgery group, ten patients did not undergo surgery because of one or more of the following reasons: patients opted for palliative care (nine), cholangitis followed by chemotherapy (one, 10.0%), and metastatic para-aortic lymph nodes on a further CT scan (one) (Supplementary Table 4).

In the PBD group and early surgery group, 119 of 144 (82.6%) and 130 of 140 (92.9%), respectively, underwent surgery at a median (range) of 19 (4–86) days and 4 (0–69) days from randomization.

Of 119 patients who underwent surgery in the PBD group, 103 (86.5%) received a pancreaticoduodenectomy (65 Whipple’s procedures, 33 pylorus-preserving pancreaticoduodenectomy (PPPD), 1 subtotal stomach-preserving PD, and 4 total pancreatectomies) (Supplementary Table 4). Among these 103 patients, 34 (33%) received venous resection and reconstruction and 5 (4.9%) received arterial reconstruction. Fifteen patients had open–close procedures in which tumors were found inoperable, and one patient had surgery at another hospital without available documentation.

In the early surgery group, 115 of 130 (88.5%) underwent curative resections (72 Whipple’s procedures, 36 PPPD, 1 subtotal stomach-preserving PD, and 6 total pancreatectomies) (Supplementary Table 4). Among these 115 patients, 41 (35.6%) and 3 (2.6%) received venous reconstruction and arterial reconstruction, respectively. Fifteen patients were found to be inoperable in open–close procedures.

### Complications After Surgery

The rate of all postoperative SAEs was similar between the PBD and early surgery group (Supplementary Table 2), namely in 23 of 119 (19.3%) and 30 of 130 patients (23.1%), respectively. After curative intent resections, numbers of pancreatic anastomosis leak (five versus four) and leaks with hepatico-, gastro-, or duodenojejunostomies (two versus four) were similar. There were numerically fewer hemorrhagic complications in the PBD group (3 versus 10). Relaparotomy was required in one and four patients in the two groups, respectively. Among patients who were operable, a lower proportion of patients in the PBD group had Clavien–Dindo classifications IVa (2.9% versus 8.7%, respectively) or V (1.0% versus 6.1%, respectively) compared with patients who had early surgery (Table 2).

### Deaths within 120 Days

**Fig. 3:**
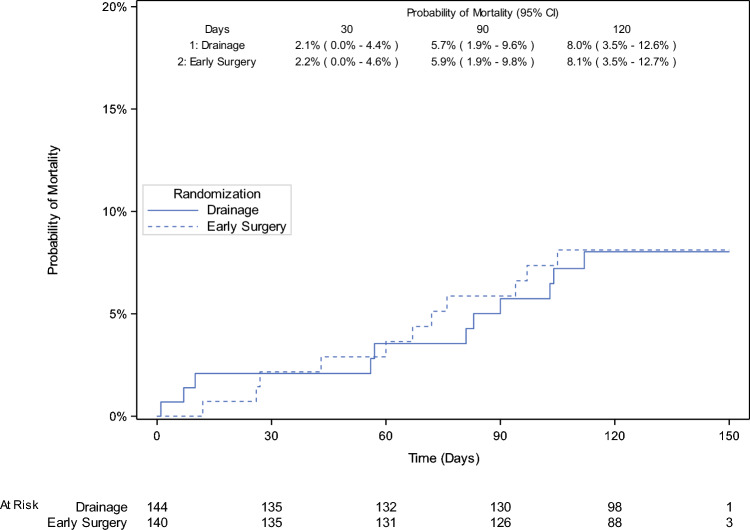
Kaplan–Meier estimate of the cumulative incidence of death to 150 days post-randomization

During follow-up, 7.6% (11/144) in the PBD group and 7.9% (11/140) in the early surgery group died (Table 2; Fig. 3). There were 8 preoperative deaths (7 versus 1), and 14 postoperative deaths (4 versus 10). Deaths after a curative-intent resection occurred in 1 of 119 (0.8%) and 7 of 130 (5.4%) in the PBD and early surgery group, respectively. The causes of death are listed in Supplementary Table 2.

### Pancreaticoduodenectomy Complications in Patients with Baseline Serum Bilirubin > 14.6 mg/dL

The number of patients with baseline serum bilirubin > 14.6 mg/dL was 53 in the PBD group and 43 in the early surgery group, of whom 41 (77.5% of 53) versus 39 (90.7% of 43) underwent surgery and 32 versus 35 had curative intent resections, respectively. The rate of surgery-related complications was 7/41 (17.1%) in the PBD group and 11/39 (28.2%) in the early surgery group.

### Per-Protocol Analysis

Compared with the modified ITT analysis, the per-protocol analysis of the primary endpoint showed similar results, i.e., a 4.8% difference in proportion of patients with at least one SAE (29.2% [40 /137] in PBD group versus 24.4% [32/131] in early surgery group; upper one-sided 95% CL, 13.8%; *P * = 0.030). The per-protocol analysis of 120-day mortality showed 7.7% (11/142) in the PBD group versus 6.0% (8/134) in the early surgery group.

## Discussion

This multicenter, randomized noninferiority trial demonstrated that the rate of complications or death for preoperative biliary drainage using SEMS was noninferior to early surgery in patients with planned curative-intent surgery to treat pancreatic or periampullary cancer. Our findings suggest that PBD using SEMS would not negatively impact patients’ outcomes and is an option for the relief of biliary obstruction before surgery.

The current RCT represents the only trial to date that compares PBD using SEMS versus early surgery without preoperative drainage. We designed the current RCT as a noninferiority trial with the hypothesis that preoperative drainage using a SEMS would not adversely affect patients’ surgical and perioperative complications. One argues that preoperative drainage adds costs of a procedure and a SEMS at marginally increased risks to patients, and that preoperative drainage in most cases is not indicated. We acknowledge that, in our RCT, the majority of patients do not require preoperative drainage. A better design would be to focus on a subgroup of patients deeply icteric. In clinical practice, patients are often drained before referrals because patients often request time to consider neoadjuvant treatments, undergo medical workups to surgery, are symptomatic from jaundice, or may have logistic difficulties in scheduling surgery. In such cases, we argue that a SEMS should be used.

Although the comparison is indirect, there are important differences between the Dutch^5^ and current RCTs other than the use of plastic versus a metal stent. First, the Dutch trial did not enroll patients with baseline serum bilirubin > 14.6 g/dL. These patients are potentially most likely to benefit from PBD. A third of our patients were deeply icteric with serum bilirubin > 14.6 g/dL. Second, our success rate of ERCP and stent insertion was 97.4%. In the Dutch trial, academic and community centers participated in ERCP procedures, with adequate drainage achieved on the first attempt in 75% of patients. The use of SEMS in our trial was associated with fewer complications (13.8% versus 47%). Our high success rate with ERCP and metal stenting and fewer complications represent the standard of academic centers. Third, although the rate of curative intent resections in the two trials was similar (86.5–88.6% in the trial compared with the overall 83% in the Dutch trial), surgical outcomes were better in our RCT. The numbers of patients with surgery-related complications were lower than those of the Dutch trial (19–23% versus 37–47%, drained or undrained). Our postoperative and overall mortality at day 120 was also lower (7.6–7.9% versus 13–15%).

ERCP complications remain a concern, though we found a lower rate of complications using SEMS compared with the reported rate associated with plastic stents.^5^ Although the 120-day mortality rates were similar (7.6% versus 7.9%, respectively), there were higher preoperative rates of mortality (4.9% versus 0.7%) and SAEs (15.2% versus 5.1%) in the PBD group compared with early surgery. This was likely associated with the longer median time from randomization to CIS for the PBD group (19.0 versus 4.0 days, respectively). The extended preoperative period for patients who receive a metal stent allows more time to consider alternative treatment options and further workup. Ultimately, fewer patients in the PBD group had surgery (82.6% versus 92.9%), with the most common reasons for no surgery being patient decision in the PBD group and transition to palliative therapy in the early surgery group. The increasing use of neoadjuvant therapy will mean more use of ERCP and metal stenting. Among patients who went to surgery, the proportion of open–close procedures^38^ in which inoperable tumors were found or failed resection was not different between the PBD group (13.4% [16/119]) compared with the early surgery group (11.5% [15/130]).

Are our results generalizable? Our study was performed in centers with expertise in ERCP. Our success rate with metal stenting was consistent with those achieved in academic centers. Furthermore, our postoperative mortality following PBD and curative intent resection was 0.8%. These results may not be easily reproduced in many centers. The 15% noninferiority margin in this trial was wide for pragmatic reasons, i.e., a narrower margin would require a substantially larger, unfeasible sample size (the enrollment period for the current sample size spanned over 9 years). Academic centers often receive referrals of patients already drained. In our screening cohort, 21% of 765 screened had received biliary drainage (Fig. 1). Our surgeons were reluctant to randomize 73 (9.5%) patients with serum bilirubin considered “too high.” Upfront surgery was a dominant strategy in patients with resectable pancreatic cancer. From the National Cancer Database in the USA, pancreatic resections were performed in 26,563 patients between 2004 and 2013; of these, 90% received upfront surgery.^39^ The relatively high proportion of vascular resection and reconstruction suggests that some borderline resectable patients were included in the study, but the number is unknown because those data were not collected. Over the course of our trial, neoadjuvant treatment has evolved to be a more common practice.^40^ The recently (2022) published ESPAC5 trial was the first to show survival benefits with neoadjuvant treatments in patients with borderline resectable pancreatic cancer.^41^ The relief of jaundice with ERCP-inserted SEMS is clearly indicated in such patients.^9^^,^^42^

## Conclusions

This RCT suggests that, in patients with periampullary cancers and signs or symptoms of biliary obstruction while awaiting surgery, the safety of SEMS is noninferior to early surgery. Future studies should aim to identify an optimal bilirubin threshold or subgroup for whom preoperative drainage is beneficial.

## Supplementary Information

Below is the link to the electronic supplementary material.

Supplementary file1 (DOCX 58 KB)

Supplementary file2 (PDF 1165 KB)

## Data Availability

The data, analytic methods, and study materials for this study may be made available to other researchers in accordance with the Boston Scientific Data Sharing Policy (http://www.bostonscientific.com/en-US/data-sharing-requests.html).
