# Supplementary material for: Preoperative Biliary Drainage with Metal Stent Versus Early Surgery in Patients with Pancreatic Cancer: A Randomized Clinical Trial
Source: Ann Surg Oncol. 2026 Apr 8;33(7):6801–11. doi: 10.1245/s10434-026-19546-9 (PMC13242453; doi:10.1245/s10434-026-19546-9)
Supplement: Supplementary file 1 — Supplementary file1 (DOCX 58 KB) [file 10434_2026_19546_MOESM1_ESM.docx]

**Supplement**

#### Supplement to: Costamagna G, Reddy DN, Chia N-h, et al. Preoperative Biliary Drainage with Metal Stent vs Early Surgery in Patients with Pancreatic Cancer[: A Randomized Clinical Trial](https://www.thelancet.com/journals/lancet/article/PIIS0140-6736(22)02076-1/fulltext)

**Supplement**

#### **Preoperative Biliary Drainage with Metal Stent vs Early Surgery in Patients with Pancreatic Cancer**[**: A Randomized Clinical Trial**](https://www.thelancet.com/journals/lancet/article/PIIS0140-6736(22)02076-1/fulltext)

**Supplement Table of Contents**

**Page Number**

Supplementary Table 1. Baseline Tumor Type in Limited Samples 3

Supplementary Table 2. Serious Adverse Events During Study Follow-Up 4

Supplementary Table 3. Additional Biliary Interventions 7

Supplementary Table 4. Curative-Intent Surgery Details 8

**Supplementary Table 1. Baseline Tumor Type in Limited Samples**

| **Tumor Characteristic** | **Preoperative Biliary Drainage** | **Early Surgery** |
| --- | --- | --- |
| **Tumor Type: Exocrine pancreas** | 43.9% (29/66) | 37.0% (20/54) |
| Ductal adenocarcinoma | 89.7% (26/29) | 90.0% (18/20) |
| Mucinous noncystic carcinoma | 23.1% (6/26) | 16.7% (3/18) |
| Adenosquamous carcinoma | 7.7% (2/26) | 22.2% (4/18) |
| Undifferentiated anaplastic carcinoma | 46.2% (12/26) | 5.6% (1/18) |
| Undifferentiated osteoclast carcinoma | 0.0% (0/26) | 5.6% (1/18) |
| Mixed ductal-endocrine carcinoma | 0.0% (0/26) | 5.6% (1/18) |
| High-grade neuroendocrine carcinoma | 3.4% (1/29) | 0.0% (0/20) |
| Small cell neuroendocrine carcinoma | 100.0% (1/1) | 0.0% (0/0) |
| Intraductal papillary-mucinous carcinoma | 0.0% (0/29) | 5.0% (1/20) |
| Ductal cells with high-grade dysplasia | 0.0% (0/29) | 5.0% (1/20) |
| **Tumor Type: Periampullary** | 48.5% (32/66) | 59.3% (32/54) |
| Adenocarcinoma (not otherwise characterized) | 84.4% (27/32) | 93.8% (30/32) |
| Adenocarcinoma pancreaticobiliary type | 6.3% (2/32) | 3.1% (1/32) |
| Papillary adenocarcinoma | 3.1% (1/32) | 0.0% (0/32) |
| Adenocarcinoma intestinal type | 0.0% (0/32) | 3.1% (1/32) |
| High-grade neuroendocrine carcinoma | 3.1% (1/32) | 0.0% (0/32) |
| Large cell neuroendocrine carcinoma | 100.0% (1/1) | 0.0% (0/0) |
| Carcinoma (not otherwise specified) | 3.1% (1/32) | 0.0% (0/32) |
| **Tumor Type: Extrahepatic bile ducts** | 4.5% (3/66) | 3.7% (2/54) |
| Adenocarcinoma | 100.0% (3/3) | 50.0% (1/2) |
| Indefinite for dysplasia | 0.0% (0/3) | 50.0% (1/2) |
| **Tumor Type: Pancreatic endocrine** | 3.0% (2/66) | 0.0% (0/54) |
| Non-secretory | 100.0% (2/2) | 0.0% (0/0) |

***** Documented tissue diagnoses from 66 samples in the drainage group and 54 samples in the no-drainage group

**Supplementary Table 2. Patients with Serious Adverse Events During Study Follow-Up**

|  | **Preoperative Biliary**  **Drainage n (%)** | **Early Surgery  n (%)** |
| --- | --- | --- |
| **Any SAE** | 40/138 (29.0) | 36/136 (26.5) |
| **Preoperative SAE** | 21 (15.2) | 7 (5.1) |
| **Protocol-specified** | 19 (13.8) | 6 (4.4) |
| Cholangitis* | 4 (2.9) | 5 (3.7) |
| Pancreatitis^†^ | 7 (5.1) | - |
| Hemorrhage after ERCP | 3 (2.2) | - |
| Persistent jaundice/biliary obstruction | 1 (0.7) | 1 (0.7) |
| Fever (undefined) | 1 (0.7) | - |
| Fatal myocardial infarction | 1 (0.7) | - |
| Fatal gastrointestinal bleed | 1 (0.7) | - |
| Fatal stroke | 1 (0.7) | - |
| Fatal subdural hematoma | 1 (0.7) | - |
| Fatal progression of cancer | 2 (1.4) | 1 (0.7) |
| Suicide | 1 (0.7) | - |
| **Non-protocol-specified** | 4 (2.9) | 1 (0.7) |
| Left leg swelling (undefined) | 1 (0.7) | - |
| Respiratory infection | 1 (0.7) | - |
| Septic arthritis | 1 (0.7) | - |
| Myocardial infarction | - | 1 (0.7) |
| Infection (Clostridium difficile) | 1 (0.7) | - |
| **Intraoperative SAE** | 1/119 (0.8) | - |
| Myocardial infarction | 1 (0.8) | - |
| **Postoperative SAE** | 23/119 (19.3) | 30/130 (23.1) |
| **Protocol-specified** | 17 (14.3) | 28 (21.5) |
| Pancreatojejunostomy or  pancreaticogastrostomy leak | 5 (4.2) | 4 (3.1) |
| Delayed gastric emptying | 2 (1.7) | 2 (1.5) |
| Biliary leakage | 2 (1.7) | 2 (1.5) |
| Gastrojejunostomy or  duodenojejunostomy leakage | - | 1 (0.8) |
| Fatal duodenojejunostomy leakage | - | 1 (0.8) |
| Intraabdominal abscess | 4 (3.4) | 8 (6.2) |
| Hemorrhage after a curative resection | 3 (2.5) | 7 (5.4) |
| Fatal hemorrhage after a curative resection | - | 3 (2.3) |
| Wound infection | 2 (1.7) | 2 (1.5) |
| Portal vein /hepatic artery thrombosis | 1 (0.8) | 1 (0.8) |
| Cholangitis* | - | 1 (0.8) |
| Pneumonia | - | 1 (0.8) |
| Fatal progression of cancer | 3 (2.5) | 2 (1.5) |
| Fatal cardiopulmonary arrest | 1 (0.8) | - |
| Fatal massive hematemesis associated  with cancer progression | - | 1 (0.8) |
| Fatal refractory shock | - | 1 (0.8) |
| Fatal sepsis‡ | - | 2 (1.5) |
| **Non-protocol-specified** | 9 (7.6) | 8 (6.2) |
| Sepsis§ | 2 (1.7) | 2 (1.5) |
| Fever¶ | 3 (2.5) | - |
| Diabetes/hypoglycemia | 1 (0.8) | 2 (1.5) |
| Hypovolemiaǁ | 1 (0.8) | - |
| Ascites | 1 (0.8) | 1 (0.8) |
| Small bowel obstruction | 1 (0.8) | 1 (0.8) |
| Delirium | - | 1 (0.8) |
| Failure to thrive | - | 1 (0.8) |
| Pain | - | 1 (0.8) |
| Pyelonephritis | - | 1 (0.8) |
| Respiratory failure | - | 1 (0.8) |
| Right hemiplegia | 1 (0.8) | - |
| Stroke | - | 1 (0.8) |
| **Re-laparotomy** | 1/138 (0.7) | 4/136 (2.9) |

The study protocol included a “protocol-specified” list of definitions of complications related to PBD or surgical treatment from a previous RCT using plastic stents (van der Gaag NA, et al. N Engl J Med 2010;362:129-37).

* Among the patients who had cholangitis (4 in PBD group, 6 in early surgery group), the median time from randomization to first documentation of cholangitis was 8.5 (range 0-75) days. Three of these patients were reported to have preoperative antibiotics.

†  The 7 patients with pancreatitis (all in the PBD group) had a median time from randomization to surgery of 30 (range 8-51) days. Six had Clavien-Dindo grade II, and one had grade IIIa. All of the pancreatitis cases resolved within 12 days.

‡ Fatal sepsis after pancreatic cancer resection (2)

§ Sepsis after E. coli (2), unknown (2)

¶ Fever after pancreatic cancer resection (2) or concurrent with biliary leakage (1)

ǁ Hypovolemia of unknown cause

|  | **Preoperative Biliary**  **Drainage n (%)** | **Early Surgery  n (%)** |
| --- | --- | --- |
| **All-cause mortality** | 11/144 (7.6) | 11/144 (7.9) |
| **Death prior to surgical attempt** | 7 (4.9) | 1 (0.7) |
| Fatal progression of cancer | 2 (1.4) | 1 (0.7) |
| Myocardial infarction | 1 (0.7) | - |
| Stroke | 1 (0.7) | - |
| Suicide | 1 (0.7) | - |
| Chronic subdural hematoma | 1 (0.7) | - |
| Gastrointestinal bleeding | 1 (0.7) | - |
| **Death after surgery** | 4/119 (3.4) | 10/130 (7.7) |
| **After curative resection** | 1 (0.8) | 7 (5.4) |
| Refractory shock (unspecified) | - | 1 (0.8) |
| Hemorrhage after curative intent surgery | - | 3 (2.3) |
| Sudden cardiopulmonary arrest | 1 (0.8) | - |
| Duodenojejunostomy leakage | - | 1 (0.8) |
| Sepsis | - | 2 (1.5) |
| **After failed resection** | 3 (2.5) | 3 (2.3) |
| Fatal progression of cancer | 3 (2.5) | 2 (1.5) |
| Massive hematemesis associated with cancer  progression | - | 1 (0.8) |

SAE serious adverse event ERCP endoscopic retrograde cholangiopancreatography

PJ pancreatico-jejunostomy PG pancreatico-gastrostomy PV portal vein

n (percent) for numbers of patients. Rows are not mutually exclusive, i.e., each patient had one or more of the listed SAEs. Denominators are intention-to-treat except for intraoperative and postoperative values where the denominators reflect the numbers of patients who had attempted surgery in each arm. Of the attempted surgeries, 103 in the drainage arm and 115 in the early surgery arm were successful.

**Supplementary Table 3. Additional Biliary Interventions**

| **Group** | **Participant Number** | **Days from Randomization to Intervention** | **Type of Intervention** | **Concurrent Health Condition**  **or Event** |
| --- | --- | --- | --- | --- |
| **Drainage** | 1* | 6 | Plastic stent placed within metal stent | Cholangitis |
| **Drainage** | 2* | 8 | Plastic stent placed within metal stent | Cholangitis |
| **Drainage** | 3* | 7 | Plastic stent placed within metal stent | Persistent jaundice |
| **Drainage** | 4* | 0 | Study metal stent removed | Pancreatitis |
| **Drainage** | 5 | 56 | Study metal stent placed | Complete distal migration |
| **Drainage** | 6 | 7 | Study metal stent exchanged, stone removal | Stent occlusion with stones |
| **Drainage** | 7 | 3 | Drainage catheter replaced, clots removed | Hemorrhage after ERCP |
| **Drainage** |  | 4 | Study metal stent exchanged | Hemorrhage after ERCP |
| **Drainage** |  | 76 | Study metal stent placed within metal stent | Cholangitis |
| **Drainage** | 8 | 86 | Second/failed attempt of curative-intent surgery | Tumor inoperable |
|  |  |  |  |  |
| **No drainage** | 1* | 6 | Study metal stent placed | Cholangitis |
| **No drainage** | 2 | 11 | Study metal stent placed | Cholangitis |
| **No drainage** | 3 | 5 | Study metal stent placed | Deep jaundice^†^ |
| **No drainage** | 4 | 7 | Study metal stent placed | Deep jaundice^†^ |
| **No drainage** | 5 | 7 | Study metal stent placed | Tumor unresectable, biliary drainage and chemotherapy as treatment |
| **No drainage** | 6* | 0 | Study metal stent placed | Surgery delayed |
| **No drainage** | 7* | 0 | Study metal stent placed | Surgery delayed |
| **No drainage** | 8 | 4 | Study metal stent placed | Surgery delayed |
| **No drainage** | 9 | 7 | Study metal stent placed | Surgery delayed |
| **No drainage** | 10 | 14 | Unknown type of stent placed at another hospital | Cholangitis |
| **No drainage** | 11* | 0 | Failed ERCP due to duodenal neoplastic infiltration | Cholangitis |
| **No drainage** |  | 1 | Transpapillary percutaneous drain | Failed ERCP |
| **No drainage** | 12* | 7 | Endoscopic nasobiliary drainage | Deep jaundice^†^ |

Color shading denotes one patient with multiple biliary interventions after baseline

*Had successful tumor resection † Deep jaundice defined as total serum bilirubin > 10 mg/dL (Dolejs S, et al. J Gastrointest Surg. 2017 Apr;21(4):647-656.)

**Supplementary Table 4. Curative-Intent Surgery Details**

|  | **Preoperative Biliary Drainage (N=144)** | **Early Surgery  (N=140)** |
| --- | --- | --- |
| **Patients who had CIS** | 82.6% (119/144) | 92.9% (130/140) |
| **Median days from randomization to CIS (range) (n)** | 19.0 (4.0,86.0) (118*) | 4.0 (0.0–69.0) (130) |
| **Blood transfusion** | 16.2% (19/117) | 14.7% (19/129) |
| **Median duration of CIS (hours)** | 8.8 (0.0–12.8) | 6.0 (1.4–13.7) |
| **Tumor resected** | 87.3% (103/118) | 88.5% (115/130) |
| Reason for unresectability (one or more) |  |  |
| Locoregional ingrowth/vascular invasion | 60.0% (9/15) | 46.7% (7/15) |
| Liver metastases | 26.7% (4/15) | 13.3% (2/15) |
| Distant metastases | 0.0% (0/15) | 6.7% (1/15) |
| Peritoneal metastases | 6.7% (1/15) | 26.7% (4/15) |
| Metastasis to lymph node(s) | 6.7% (1/15) | 13.3% (2/15) |
| Benign tumor | 6.7% (1/15) | 0 |
| **Type of resections performed** |  |  |
| Whipple | 63.1% (65/103) | 62.6% (72/115) |
| **Pylorus-Preserving Pancreaticoduodenectomy** | 32.0% (33/103) | 31.3% (36/115) |
| Total pancreatectomy | 3.9% (4/103) | 5.2% (6/115) |
| Subtotal Stomach-Preserving Pancreaticoduodenectomy | 1.0% (1/103) | 0.9% (1/115) |
| **Pancreatic remnant present** | 95.1% (98/103) | 93.9% (108/115) |
| Pancreatic anastomosis performed | 90.8% (89/98) | 92.6% (100/108) |
| Pancreaticojejunostomy (PJ) technique | 91.0% (81/89) | 98.0% (98/100) |
| Pancreaticogastrostomy (PG) technique | 9.0% (8/89) | 2.0% (2/100) |
| **Patients with no CIS** | 17.4% (25/144) | 7.1% (10/140) |
| **Reasons** (one or more per patient) |  |  |
| Patient decision | 44.0% (11/25) | 20.0% (2/10) |
| Transition to PAL | 36.0% (9/25) | 90.0% (9/10) |
| Reason for Transition |  |  |
| Progression of disease | 88.9% (8/9) | 55.6% (5/9) |
| Decline in physiological status | 11.1% (1/9) | 44.4% (4/9) |
| CIS scheduled after 120 days post randomization | 4.0% (1/25) | 0.0% (0/10) |

CIS Curative-Intent Surgery

*Procedural data missing for one additional patient in the biliary drainage group who had CIS
