# Supplementary material for: Preoperative Biliary Drainage with Metal Stent Versus Early Surgery in Patients with Pancreatic Cancer: A Randomized Clinical Trial
Source: Ann Surg Oncol. 2026 Apr 8;33(7):6801–11. doi: 10.1245/s10434-026-19546-9 (PMC13242453; doi:10.1245/s10434-026-19546-9)
Supplement: Supplementary file 2 — Supplementary file2 (PDF 1165 KB) [file 10434_2026_19546_MOESM2_ESM.pdf]

Protocol

This trial protocol has been provided by the authors to give readers additional information about their work.

Protocol for: Costamagna G, Reddy DN, Chia N-h, et al. Preoperative Biliary Drainage with Metal Stent vs Early Surgery in Patients with Pancreatic Cancer: A Randomized Clinical Trial

This supplement contains the following items:

1. Original protocol, final protocol, summary of changes

2. Original statistical analysis plan, final statistical analysis plan including summary of changes

All documents are from Preoperative Biliary Drainage RCT Clinical Protocol E7059.

The original protocol is version AC, which was the first version submitted to the ethics committees.

The final protocol is version AD, which was approved by the ethics committees after submission of amendments.

Summary of changes from the original to final protocol are provided as a separate page.

The original statistical analysis plan is SAP version AA.

The final statistical analysis plan is SAP version B.

Summary of changes from the original to final statistical analysis plan is at the beginning of SAP version B.

Table of Contents

Original protocol	5
Final protocol	49
Summary of changes to the protocol since the original version	93
Original statistical analysis plan	94
Final statistical analysis plan including changes since the original version	109

Original Protocol

Randomized Multi-Center Study Comparing No Drainage to Preoperative Biliary Drainage Using Metal Stents in Patients with Resectable Pancreatic or Periampullary Cancer

Preoperative Biliary Drainage RCT

CLINICAL PROTOCOL

E7059

Sponsored By

Boston Scientific Corporation
100 Boston Scientific Way
Marlborough, MA 01752-1234
USA

This protocol contains confidential information for use by the Investigators and their designated representatives participating in this clinical investigation. It should be held confidential and maintained in a secure location.

Do not copy or distribute without written permission from Boston Scientific Corporation.

Contact Information

Role	Contact
**Clinical Contact**	Lina Ginnetti<br>Clinical Project Manager<br>Boston Scientific Corporation<br>100 Boston Scientific Way<br>Marlborough, MA 01752-1234<br>USA
**Coordinating Principal Investigators**	Professor Guido Costamagna, MD<br>Università Cattolica del Sacro Cuore<br>Policlinico A. Gemelli<br>U.O. di Endoscopia Digestiva Chirurgica<br>Largo A. Gemelli, 8<br>00168 Rome, Italy<br><br>Professor James Lau, MD<br>Prince of Wales Hospital<br>30-32 Ngan Shing Street<br>Shatin<br>New Territories, Hong Kong

Original Release: May 16, 2012

Current Version: October 16, 2012

Do not copy or distribute without written permission.

Investigator's Signature Page

STUDY TITLE: Randomized Multi-Center Study Comparing No Drainage to Preoperative Biliary Drainage Using Metal Stents in Patients with Resectable Pancreatic or Periampullary Cancer

STUDY CENTER: ____

(Print name of study center)

PROTOCOL VERSION: AC

We, the undersigned, have read and understand the protocol specified above and agree on its content. We agree to perform and conduct the study as described in the protocol. In addition, when applicable, we agree to enlist sub-investigators who also agree to perform and conduct the study as described in the protocol.

____ ________________________

Principal Investigator Date

Print name:

____ ________________________

Co-Principal Investigator (if applicable) Date

Print name:

Protocol Synopsis

**Full Title**	**Randomized Multi-Center Study Comparing No Drainage to Preoperative Biliary Drainage Using Metal Stents in Patients with Resectable Pancreatic or Periampullary Cancer**
**Short Title**	Preoperative Biliary Drainage RCT
**Objective(s)**	To demonstrate that preoperative biliary drainage using self-expanding metal stents (SEMS) improves overall surgical outcomes in patients undergoing pancreaticoduodenectomy for treatment of pancreatic or periampullary cancer.
**Test Device**	Devices:<br>WallFlex Biliary RX Fully Covered stent<br>Note: WallFlex Biliary RX Uncovered stents may be used in in some cases as outlined in the protocol<br>Cleared Indication:<br>The WallFlex Biliary stents are indicated for use in the palliative treatment of biliary strictures produced by malignant neoplasms; the WallFlex FC stent is also indicated for the treatment of benign biliary strictures per CE Mark.
**Study Design**	• Prospective, multi-center, randomized, post-market (on label)<br>• Patients will be block randomized at baseline in a 1:1 ratio between **Group 1:** No Pre-Operative Biliary Drainage and **Group 2:** Pre-Operative Biliary Drainage with a SEMS.
**Planned Number of Subjects**	294 total number of subjects:<br>• Group 1: 147<br>• Group 2: 147
**Planned Number of Centers / Countries**	7-12 sites<br><br>Countries: Australia, Belgium, China, France, Germany, India, Italy, Japan (this list of countries may change)
**Primary Endpoint**	Serious pre-operative, operative and post-operative adverse events to 120 days post randomization or to 30 days post-surgery, whichever comes last
**Secondary Endpoints**	1. Adverse events: rate, severity, seriousness, relatedness to stent or endoscopic or surgical procedure, impact on time of surgery, length of hospitalization and ICU stay

	2. Time to surgery<br>3. Curative Intent Surgery details pertaining to intraoperative assessment of resectability, surgical resection and reconstruction techniques<br>4. Intraoperative blood loss and blood transfusions, duration of surgery<br>5. Biliary obstructive symptoms assessment (Week 2, surgery/transition to palliation, 30 day post-operative follow up and 120 day follow up)<br>6. Improvement of LFT levels as relative to baseline (Week 2, surgery/transition to palliation, 30 day post-operative follow up and 120 day follow up)<br>7. Stent placement success: ability to deploy the stent in satisfactory position across the stricture (Group 2)<br>8. Stent removal success: successful SEMS removal, either *en bloc* at time of surgery or endoscopically prior to surgery without stent removal related SAEs<br>9. Number, type, reason and timing of biliary re-interventions<br>10. Number and duration of hospital and ICU admissions
**Follow-up Schedule and Assessments**	• **Screening:** Count all consecutive patients seen at investigational site presenting with biliary obstructive symptoms and suspicion of pancreatic cancer, distal common bile duct cholangiocarcinoma or peri-ampullary cancer. Of those, a subset will be invited to participate in trial: Informed Consent (enrollment) and Eligibility Criteria Assessment<br>• **Baseline Visit (Day 0):** Demographics, Medical History, Tumor Diagnosis, Staging and Characteristics, Assessment of Biliary Obstructive Symptoms, Laboratory Tests, Randomization<br>• **Stent Placement Procedure Visit (Group 2 only, Day 0):** Stent Details, Procedure Details, Adverse Events (AEs)<br>• **Pre-Operative Visit (Week 2):** Assessment of Biliary Obstructive Symptoms, Laboratory Tests, AEs<br>***Group 1 (No Pre-Operative Biliary Drainage):*** Patients will undergo resection per institutional standard of practice. Time to surgery is not to exceed 4 weeks unless there is a need to delay planned surgery due to a complication, biliary obstructive symptoms, decline in patient physiologic status, or other factors that preclude surgery.<br>***Group 2 (Pre-Operative Biliary Drainage with a SEMS):*** Patients will undergo resection after resolution of jaundice is achieved (defined as Bilirubin below 100μmol per liter), or at 4 weeks, whichever comes first.

	• **Biliary Reintervention Visit (as needed):** Timing, Reason for Biliary Reintervention, Type of Biliary Reintervention, AEs<br>Group 1 patients who fail their treatment algorithm by requiring biliary drainage prior to surgery will receive a study SEMS.<br>Group 2 patients who require re-stenting or placement of a stent for a new stricture, will receive a study SEMS.<br>• **Curative Intent Surgery:** Assessment of Biliary Obstructive Symptoms, Laboratory Tests, Operative Details, Stent Removal, Blood Loss, Intra- and Post-Operative Transfusion, Post-Operative Course, Specimen Pathology, AEs<br>• **Transition to Palliative Management Visit (as needed):** Assessment of Biliary Obstructive Symptoms, Laboratory Tests, AEs<br>• **Post-Operative Follow up Visit (30 days Post-Surgery, if applicable):** Assessment of Biliary Obstructive Symptoms, Laboratory Tests, AEs<br>• **Long Term Follow-up Visit (120 days--150 days post randomization):** Assessment of Biliary Obstructive Symptoms, Laboratory Tests, AEs<br>Patients who undergo surgery as planned will be evaluated at 30 days post-surgery and will be followed up to 120 days after randomization.<br>Patients with delay in planned surgery may require follow up longer than 120 days to allow for a 30 day post-surgery follow up visit but follow up is not to exceed 150 days post-randomization.<br>Patients with a delay in planned surgery whose rescheduled surgery is unable to occur within 120 days will be followed up to 120 days after randomization.<br>Patients who do not undergo surgery as planned due to conversion to palliative management or patient choice will be followed up to 120 days after randomization. If patient's course of treatment requires placement of a biliary stent, stenting will be done per standard of care at each institution. If a metal stent is required, a study WallFlex Biliary FC stent will be placed.
**Key Inclusion Criteria**	1. Age 18 or older<br>2. Willing and able to comply with the study procedures and provide written informed consent to participate in the study<br>3. Diagnosis of probable pancreatic cancer, distal common bile duct (CBD) cholangiocarcinoma and other periampullary cancers (histology not required)<br>4. Biliary obstructive symptoms or signs

	5. Bilirubin level at/above 100 μmol per liter (5.8 mg/dL)<br>6. Distal biliary obstruction consistent with pancreatic cancer, distal CBD cholangiocarcinoma or other periampullary malignancy<br>7. Location of distal biliary obstruction is such that it would allow the proximal end of a stent to be positioned at least 2 cm from the hilum<br>8. Patients deemed as resectable by pancreatic protocol CT or MRI<br>9. Surgical candidate per pancreatobiliary surgeon after multi-disciplinary discussion<br>10. Surgery intent within 4 weeks<br>11. Endoscopic and surgical treatment to be provided by same team
**Key Exclusion Criteria**	1. Biliary strictures caused by confirmed benign tumors<br>2. Biliary strictures caused by malignancies other than pancreatic, cancer, distal CBD cholangiocarcinoma and other periampullary cancers<br>3. Surgically altered biliary tract anatomy, not including prior cholecystectomy<br>4. Neoadjuvant chemotherapy for current malignancy<br>5. Palliative indication due to reasons other than surgical candidate status<br>6. Previous biliary drainage by ERCP/PTC<br>7. Patients for whom endoscopic techniques are contraindicated<br>8. Participation in another investigational trial within 90 days<br>9. Pregnancy
**Primary Statistical Hypothesis**	A literature search of preoperative biliary drainage with self-expanding metal stents in patients with pancreatic or periampullary cancer yielded eight articles with 305 patients.[1-8]<br>A meta-analysis of the probability for pre-operative, operative and peri-operative complications was performed. A rate of 24.2% [95% CI: 13.2%, 37.2%] was calculated using the eight articles.<br><br>Statistical testing will be performed to determine if the rate of complications for the *Pre-Operative Biliary Drainage with SEMS* group is non-inferior to the *No Pre-Operative Biliary Drainage* group. The null hypothesis is that the complication rate is inferior in the *Pre-Operative Biliary Drainage with SEMS* versus the *No Pre-Operative Biliary Drainage* group:<br><br>$Ho: \pi_{test} - \pi_{control} \geq \Delta$ (Inferior)

	$Ha: \pi_{test} - \pi_{control} < \Delta$ (Non-inferior)<br><br>where π_{test} and $\pi_{control}$ are the probabilities of having pre-operative, operative and peri-operative complications in the *Pre-Operative Biliary Drainage with SEMS* arm and *No Pre-Operative Biliary Drainage* arm respectively, and Δ is defined as the non-inferiority margin.<br><br>The sample size was calculated for a one-sided 0.050 exact Farrington-Manning test using StatXact 9®. If the P value from the exact Farrington-Manning test is <0.05 then the *Pre-Operative Biliary Drainage with SEMS* group will be considered non-inferior to the *No Pre-Operative Biliary Drainage* group. The expected probability of complications in the *Pre-Operative Biliary Drainage with SEMS* arm and *No Pre-Operative Biliary Drainage* arm is 37.2%, which was taken from the upper limit of the 95% CI from the meta-analysis described above and from the only available Level 1 study comparing no drainage to preoperative stenting in which the complication rate was reported to be 39% in the no drainage arm.[9] The non-inferiority margin (Δ) is 15%. Given these assumptions and a one-sided 5% significance level, 2 x 132 = 264 subjects will provide 80% power to reject the null hypothesis, that the *Pre-Operative Biliary Drainage with SEMS* group is inferior to the *No Pre-Operative Biliary Drainage* group.<br><br>To compensate for possible loss of subjects after enrollment and complete assessment of inclusion/exclusion criteria, an additional 10% of subjects will be enrolled, for a total of 2 x 147 = 294 subjects.

TABLE OF CONTENTS

1. INTRODUCTION 17

1.1. **Pancreatic and Periampullary cancer** 17

1.2. **Treatment of Pancreatic/Periampullary Cancer** 17

1.3. **Biliary Drainage in Patients with Pancreatic/Periampullary Cancer** 18

2. PRIMARY OBJECTIVE 18

3. DESIGN 18

3.1. **Scale, Duration and Investigators** 18

3.2. **Treatment Assignment** 19

4. ENDPOINTS 21

4.1. **Primary Endpoint** 21

4.2. **Secondary Endpoints** 21

5. SUBJECT SELECTION 21

5.1. **Study Population and Eligibility** 21

5.2. **Inclusion Criteria** 21

5.3. **Exclusion Criteria** 22

6. STUDY DEVICES 22

7. STUDY VISITS 24

7.1. **Visit Schedule** 24

7.2. **Screening** 26

7.3. **Baseline Visit (Day 0)—Office Visit** 26

7.4. **Stent Placement Procedure Visit (Group 2 subjects only)—Office Visit (may occur during Baseline visit)** 26

7.5. **Pre-Operative Visit (Week 2)—Telephone or Office Visit** 27

7.6. **Biliary Reintervention Visit (Group 1 or 2) —as needed** 27

7.7. **Curative Intent Surgery—Office Visit (within 4 weeks, or delayed surgery by 120 days post-randomization)** 27

7.8. **Transition to Palliative Management Visit (if applicable)—Telephone or Office Visit** 28

7.9. **Post-Operative Follow-up Visit (30 days Post-Surgery, if applicable)—Telephone or Office Visit** 28

7.10. **Long Term Follow-up Visit (120 days—150 days post randomization)—Telephone or Office Visit** 29

7.11. **Study Completion** 29

8. STATISTICAL CONSIDERATIONS 29

8.1. **Hypothesis** 29

8.2. **Sample Size** 30

8.3. **Analysis Populations** 30

8.3.1. Enrolled Cohort 30

8.3.2. Intent-to-Treat Cohort 30

8.3.3. Per-Protocol Cohort 30

8.4. **Data Analyses** 31

8.4.1. Baseline Data 31

8.4.2. Post-Procedure Endpoints 31

8.4.3. Subgroup Analyses 31

8.4.4. Justification of Pooling 31

8.4.5. Multivariable Analyses 31

8.4.6. Changes to Planned Analyses 32

9. POTENTIAL RISKS AND BENEFITS 32

9.1. **Anticipated Adverse Device Effects with Use of WallFlex Biliary Stent** 32

9.2. **Anticipated Adverse Events in Patients Without Pre-Operative Biliary Drainage** 33

9.3. **Anticipated Surgical Adverse Events** 33

9.4. **Risk Minimization Actions** 33

9.5. **Anticipated Benefits** 34

9.6. **Risk to Benefit Rationale** 34

10. SAFETY REPORTING 34

10.1. **Definitions and Classification** 34

10.2. **Relationship to Study Device(s)** 36

10.3. **Investigator Reporting Requirements** 36

10.3.1. Serious Adverse Events 36

10.3.2. Adverse Events 36

10.3.3. Device Failures, Malfunctions, and Product Nonconformities 37

10.4. **Boston Scientific Device Deficiencies** 37

10.5. **Reporting to Regulatory Authorities / IRBs / ECs / Investigators** 37

11. BIBLIOGRAPHY **37**

12. APPENDICES: SPONSOR REQUIRED PROTOCOL SECTIONS **39**

12.1. Data Management **39**
12.1.1. Data Collection, Processing, and Review 39
12.1.2. Data Retention 40

12.2. Amendments **40**

12.3. Deviations **40**

12.4. Device/Equipment Accountability **41**

12.5. Compliance **41**
12.5.1. Statement of Compliance 41
12.5.2. Investigator Responsibilities 41
12.5.3. Delegation of Responsibility 43

12.6. Institutional Review Board/ Ethics Committee **43**

12.7. Sponsor Responsibilities **43**

12.8. Insurance **44**

12.9. Monitoring **44**

12.10. Informed Consent **44**

12.11. Committees **45**
12.11.1. Safety Monitoring Process 45

12.12. Suspension or Termination **46**
12.12.1. Premature Termination of the Study 46
12.12.2. Criteria for Premature Termination of the Study 46
12.12.3. Termination of Study Participation by the Investigator or Withdrawal of IRB/ EC Approval 46
12.12.4. Requirements for Documentation and Subject Follow-up 46
12.12.5. Criteria for Suspending/Terminating a Study Center 47

12.13. Publication Policy **47**

12.14. Definitions of complication criteria (per van der Gaag article): **47**

1. Introduction

1.1. *Pancreatic and Periampullary cancer*

Pancreatic cancer is the fourth leading cause of cancer death in the US and worldwide with 217,000 new cases/year and 213,000 deaths worldwide.[10, 11] Relative 1-year and 5-year survival rates are 26% and 6% respectively.[11]

Pancreatic tumors are usually asymptomatic until the disease is advanced. Presenting symptoms may include weight loss, jaundice, pain, nausea, dyspepsia and depression.[12] Diagnosis and staging is usually achieved with a pancreas protocol CT, a triphasic cross-sectional imaging with thin slices using multidetector CT[13-16]which allows for an assessment of the vascular invasion of the tumor, a major factor in determining resectability. In patients where CT is not possible or contraindicated, MRI with contrast can be used to diagnose and stage pancreatic cancer.[17] Pancreas protocol MRI has been emerging as an equivalent alternative to CT.[18] Endoscopic ultrasound (EUS) can be used complementary to CT to provide additional vascular invasion information or to evaluate periampullary masses.[19, 20] Although histologic diagnosis is not necessary before surgical treatment, a biopsy can be taken using fine-needle aspiration with either EUS-guidance (preferred) or CT-guidance.[18] Diagnostic staging laparoscopy is also utilized, and is mostly valuable when there is a need to rule out sub-radiologic metastases (especially for body and tail lesions).[21, 22]

Based on results from imaging studies described above, patients with pancreatic cancer can be divided into four prognostic subgroups (Stages I-IV) to allow for choice of proper treatment course. These groups are based on local tumor resectability status and presence or absence of distant disease as defined by the tumor-node-metastasis (TNM) staging system of the American Joint Commission on Cancer (AJCC).[23] Patients in whom the primary tumor can be removed are classified as localized resectable (Stages I and II), and patients whose tumors are unresectable are classified as locally advanced (Stage III) and metastatic (Stage IV).[24]

Criteria for resectability vary but usually include the absence of distant metastases and no tumor involvement of major arteries; in cases of venous invasion of the tumor, there must be a suitable segment of portal vein (above) and superior mesenteric vein (below) the site of venous involvement to allow for venous reconstruction.[25]

1.2. *Treatment of Pancreatic/Periampullary Cancer*

The only potential curative treatment option for patients with resectable pancreatic/periampullary cancers is curative-intent-surgery (CIS) by pancreaticoduodenectomy (Whipple procedure)[26] with a long-term survival rate of 20% and a median survival of 12-20 months.[24] Only 15-20% of patients presenting with pancreatic cancer have potentially resectable tumors.[27]

Most pancreatic cancers arise in the head of the pancreas and often cause bile duct obstruction resulting in jaundice, while body and tail lesions are insidious in their development and usually far advanced when detected. Therefore most patients who are candidates for CIS have tumors in the head of the pancreas.[28] The most common type of

pancreatic cancer is adenocarcinoma, originating from the pancreatic duct cells[24] and accounting for over 90% of pancreatic malignancies.[12]

1.3. *Biliary Drainage in Patients with Pancreatic/Periampullary Cancer*

Biliary obstruction associated with pancreatic/periampullary cancer occurs in up to 70% of patients.[29] Hyperbilirubinemia resulting from biliary obstruction adversely affects liver, cardiovascular and renal function,[26] and the subsequent cholestasis which is thought to impair immune response and clotting[1] may negatively impact the outcome of CIS. Preoperative biliary drainage potentially reverses these factors and could result in improved outcome of pancreaticoduodenectomy.[26]

Preoperative biliary decompression has traditionally been achieved with plastic stents, however, these stents have been associated with high complication rates and relatively low success rates in preoperative management of biliary obstruction.[1-3, 9, 30]

In recent publications, SEMS have been shown to be superior to plastic stents for preoperative biliary drainage in pancreatic cancer[1] because of longer patency achieved by a greater stent diameter,[31] fewer ERCP procedures, and fewer episodes of cholangitis/cholestasis due to stent occlusion while awaiting surgery (15% using SEMS vs 93% using plastic stents).[2]

2. Primary Objective

The primary objective is to demonstrate that preoperative biliary drainage using SEMS improves overall surgical outcomes in patients undergoing pancreaticoduodenectomy for treatment of pancreatic or periampullary cancer.

3. Design

3.1. *Scale, Duration and Investigators*

This is a post-market (on label), prospective, multi-center, randomized, non-blinded study.

Patients will be followed for 120 days after randomization. Only patients who experience a delay in their planned surgery due to factors such as but not limited to complications, biliary obstructive symptoms, patient physiologic status, etc. may be followed up to 150 days to allow for a 30 days post-surgery follow up visit.

There will be 7-12 participating centers which will include countries in Europe and Asia with anticipated enrollment of 294 patients, 147 per group.

At each investigational center, there will be one principal investigator (PI) and at least one Co-PI. At a minimum, there will be one gastroenterologic endoscopist and one pancreaticobiliary surgeon among the investigators. Although more than two investigators per center are allowed, it is encouraged that the number be kept small.

3.2. *Treatment Assignment*

Patients will be randomized at baseline in equal proportions of 1:1 ratio between Group 1 and Group 2 as follows:

- **Group 1**: No pre-operative biliary drainage (patients will not receive a stent)
- **Group 2**: Pre-operative biliary drainage with a SEMS

Block randomization through an online database system will be used. Randomization will be stratified by study center.

Figure 1 below provides an overview of various patient management scenarios in the form of a study flow chart.

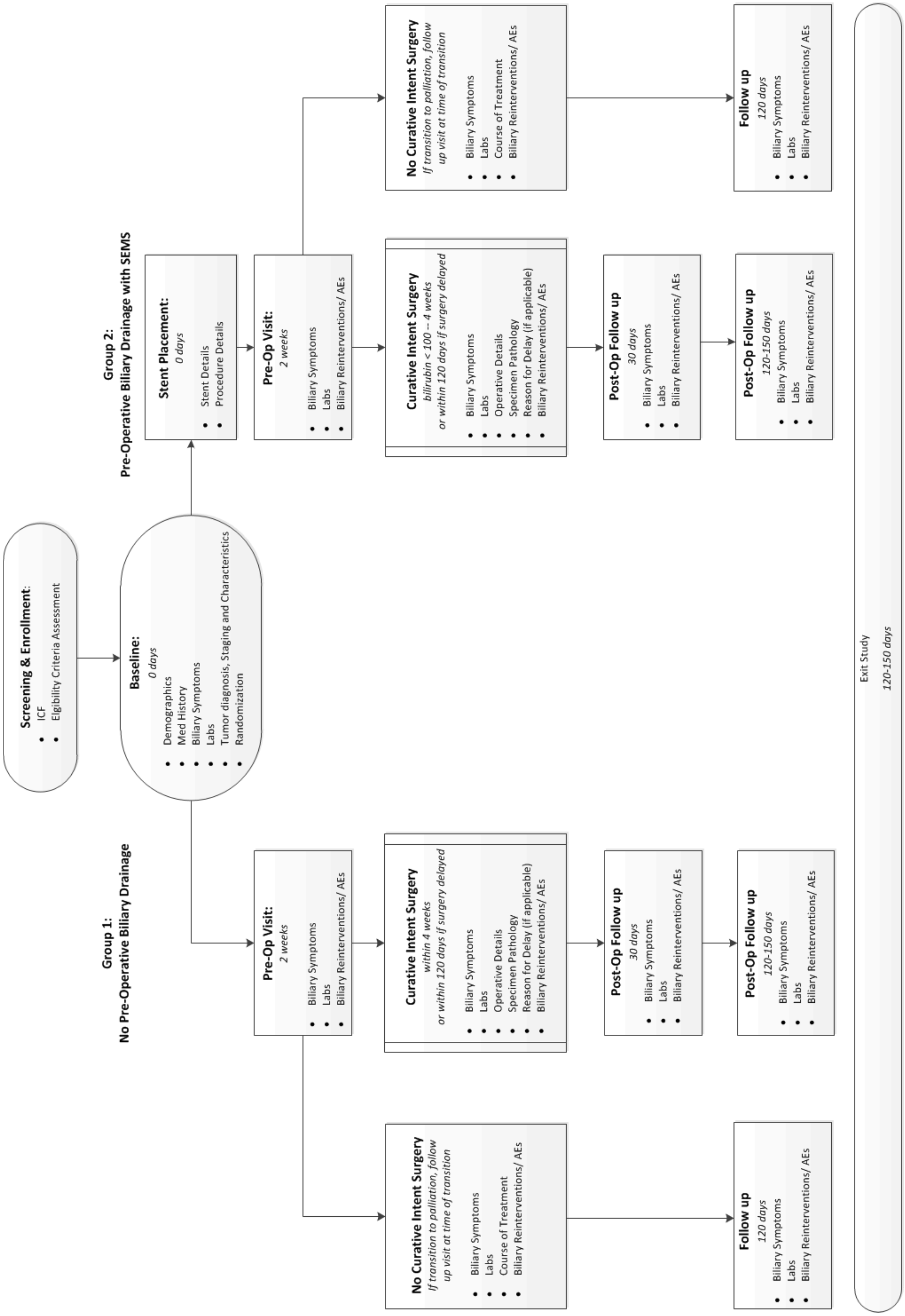
Screening & Enrollment:
ICF
Elgibility Criteria Assessment
Baseline:
0 days
Demographics
Med History
Biliary Symptoms
Labs
Tumor diagnosis, Staging and Characteristics
Randomization
Group 1:
No Pre-Operative Biliary Drainage
Pre-Op Visit:
2 weeks
Biliary Symptoms
Labs
Biliary Reinterventions/ AEs
Curative Intent Surgery
within 4 weeks
or within 120 days if surgery delayed
Biliary Symptoms
Labs
Operative Details
Specimen Pathology
Reason for Delay (if applicable)
Biliary Reinterventions/ AEs
Post-Op Follow up
30 days
Biliary Symptoms
Labs
Biliary Reinterventions/ AEs
Post-Op Follow up
120-150 days
Biliary Symptoms
Labs
Biliary Reinterventions/ AEs
No Curative Intent Surgery
If transition to palliation, follow up visit at time of transition
Biliary Symptoms
Labs
Course of Treatment
Biliary Reinterventions/ AEs
Follow up
120 days
Biliary Symptoms
Labs
Biliary Reinterventions/ AEs
Group 2:
Pre-Operative Biliary Drainage with SEMS
Stent Placement:
0 days
Stent Details
Procedure Details
Pre-Op Visit:
2 weeks
Biliary Symptoms
Labs
Biliary Reinterventions/ AEs
Curative Intent Surgery
bilirubin < 100 – 4 weeks
or within 120 days if surgery delayed
Biliary Symptoms
Labs
Operative Details
Specimen Pathology
Reason for Delay (if applicable)
Biliary Reinterventions/ AEs
Post-Op Follow up
30 days
Biliary Symptoms
Labs
Biliary Reinterventions/ AEs
Post-Op Follow up
120-150 days
Biliary Symptoms
Labs
Biliary Reinterventions/ AEs
No Curative Intent Surgery
If transition to palliation, follow up visit at time of transition
Biliary Symptoms
Labs
Course of Treatment
Biliary Reinterventions/ AEs
Follow up
120 days
Biliary Symptoms
Labs
Biliary Reinterventions/ AEs
Exit Study
120-150 days

4. Endpoints

4.1. *Primary Endpoint*

Serious pre-operative, operative and post-operative adverse events to 120 days post randomization or to 30 days post-surgery, whichever comes last

4.2. *Secondary Endpoints*

1. Adverse events: rate, severity, seriousness, relatedness to stent or endoscopic or surgical procedure, impact on time of surgery, length of hospitalization and ICU stay
2. Time to surgery
3. Curative Intent Surgery details pertaining to intraoperative assessment of resectability, surgical resection and reconstruction techniques
4. Intraoperative blood loss and blood transfusions, duration of surgery
5. Biliary obstructive symptoms assessment (Week 2, surgery/transition to palliation, 30 day post-operative follow up and 120 day follow up)
6. Improvement of LFT levels as relative to baseline (Week 2, surgery/transition to palliation, 30 day post-operative follow up and 120 day follow up)
7. Stent placement success: ability to deploy the stent in satisfactory position across the stricture (Group 2)
8. Stent removal success: successful SEMS removal, either *en bloc* at time of surgery or endoscopically prior to surgery without stent removal related SAEs
9. Number, type, reason and timing of biliary re-interventions
10. Number and duration of hospital and ICU admissions

5. Subject Selection

5.1. *Study Population and Eligibility*

All consecutive patients seen at an investigational site presenting with biliary obstructive symptoms with suspicion of pancreatic cancer, periampullary cancer or distal CBD cholangiocarcinoma will be counted. Of those, a subset will be invited to participate in trial and is considered enrolled after signing the study specific Informed Consent Form (ICF). A screening log will be maintained to document the assessment of eligibility criteria provided in Section 5.2 and Section 5.3.

5.2. *Inclusion Criteria*

Subjects who meet all of the criteria listed below may be given consideration for inclusion in this clinical investigation, provided no exclusion criterion (see Section 5.3) is met.

1. Age 18 or older
2. Willing and able to comply with the study procedures and provide written informed consent to participate in the study
3. Diagnosis of probable pancreatic cancer, distal common bile duct (CBD) cholangiocarcinoma and other periampullary cancers (histology not required)
4. Biliary obstructive symptoms or signs
5. Bilirubin level at/above 100μmol per liter (5.8 mg/dL)
6. Distal biliary obstruction consistent with pancreatic cancer, distal CBD cholangiocarcinoma or other periampullary malignancy
7. Location of distal biliary obstruction is such that it would allow the proximal end of a stent to be positioned at least 2cm from the hilum
8. Patients deemed as resectable by pancreatic protocol CT or MRI
9. Surgical candidate per pancreatobiliary surgeon after multi-disciplinary discussion
10. Surgery intent within 4 weeks
11. Endoscopic and surgical treatment to be provided by same team

5.3. *Exclusion Criteria*

Subjects who meet any one of the following criteria will be excluded from this clinical study.

1. Biliary strictures caused by confirmed benign tumors
2. Biliary strictures caused by malignancies other than pancreatic cancer, distal CBD cholangiocarcinoma and other periampullary cancers
3. Surgically altered biliary tract anatomy, not including prior cholecystectomy
4. Neoadjuvant chemotherapy for current malignancy
5. Palliative indication due to reasons other than surgical candidate status
6. Previous biliary drainage by ERCP/PTC
7. Patients for whom endoscopic techniques are contraindicated
8. Participation in another investigational trial within 90 days
9. Pregnancy

6. Study Devices

The WallFlex Biliary RX Fully Covered stent will be the default choice for treatment of patients in Group 2. A WallFlex Biliary RX Uncovered stent may be used at the joint discretion of the endoscopic and surgical investigators for documented reasons related to the ductal anatomy, namely stricture involving the cystic duct confluence, low cystic duct confluence, or other anatomic considerations to be specified.

The WallFlex Biliary RX Fully Covered and the WallFlex Biliary RX Uncovered Stent Systems are indicated for use in the palliative treatment of biliary strictures produced by malignant neoplasms and the WallFlex Biliary RX Fully Covered Stent System is also indicated for treatment of benign biliary strictures, per CE Mark. For a detailed description of the WallFlex Biliary Stent Systems, please reference the Directions for Use (DFU) included in each device package.

Investigators should use the WallFlex Biliary RX Fully Covered and Uncovered Stent Systems in accordance with the DFUs.

Study devices are labeled on the box and inner pouch and contain information including but not limited to: device name and dimensions, lot number, expiration date, name of legal manufacturer, and investigational use statement. Device labeling will be provided in local language(s) as per national regulations.

Study devices will be available in the following dimensions:

WallFlex Biliary RX Stent	Diameter	Length	Delivery System Diameter	Guidewire Diameter
**Uncovered**	8 mm<br>10 mm	40, 60, 80<br>40, 60, 80	8 Fr	.035”
**Fully Covered**	8 mm<br>10 mm	60, 80mm<br>40, 60, 80mm	8.5 Fr	.035”

Stent placement should be such that the proximal end of the stent is minimum 2 cm from the hilum. Performing a biliary or pancreatic sphincterotomy or enlarging a prior sphincterotomy will be done at the discretion of the endoscopist.

In case of a failed stent placement due to a device event, a new attempt to place a stent will be made. If a stent placement is not possible due to non-device related reasons (such as inability to cannulate the CBD or reach the papilla, extensive tumor growth at site of papilla, etc.), interventional radiologic (IR) access is allowed and, where possible, should be associated with placement of a study stent over a transhepatically inserted guide-wire in a "rendez-vous" procedure. Percutaneous transhepatic cholangiograpy (PTC) and a subsequent internal drainage with a stent placement may be done at the same time (one-stage procedure), or PTC with external drainage may be performed 2 -3 days before stent insertion (two-stage procedure) per standard of practice. If access to the biliary tree or endoscopic placement of the study stent through a “rendez-vous” procedure fails and patient requires percutaneous transhepatic biliary drainage (PTBD), then the patient will exit the study.

7. Study Visits

7.1. *Visit Schedule*

The schedule of observations and assessments to take place during the study is outlined in **Table 1** below.

Table 1. Study Event Schedule

Study Visit	Screening	Baseline/ Stent Placement Procedure	Pre-Operative Visit Week 2†	Biliary Reintervention (if applicable)	Curative Intent Surgery	Transition to Palliative Treatment Visit (if applicable)	30 Days Post-Op Follow up Visit (if applicable)	Long Term Follow up Visit 120 Days*
ICF	X							
Eligibility Criteria Assessment	X							
Demographics		X						
Medical History		X						
Assessment of Biliary Obstructive Symptoms		X	X		X	X	X	X
Laboratory Tests		X	X		X	X	X	X
Tumor Diagnosis, Staging & Characteristics		X						
Randomization		X						
Stent Details		X		X (if applicable)				
Procedure Details		X		X (if applicable)				
Operative Details					X			
Specimen Pathology					X			
Stent Removal				X (if applicable)	X (if applicable)			
Adverse Events	X (as applicable)							
Protocol Deviations	X (as applicable)							

* Visit may occur up to 150 days post-randomization; † Visit may be conducted in the office or via telephone with liver function tests drawn and sent from local clinic/hospital.

7.2. *Screening*

- Informed Consent
- Eligibility Criteria Assessment

No study-specific testing will be conducted until the subject has signed the study specific informed consent form (ICF).

Informed consent must be obtained for all subjects who are potential study candidates. Subjects will be asked to sign the ICF before any study-specific tests or procedures are performed. The ICF is study-specific and must be approved by the Institutional Review Board (IRB)/Ethics Committee (EC) at each center. Study personnel should explain that even if a subject agrees to participate in the study and signs the study specific ICF, the screening process may demonstrate that the subject is not a suitable candidate for the study.

7.3. *Baseline Visit (Day 0)—Office Visit*

- Demographics
- Medical History
- Tumor Diagnosis, Staging and Characteristics
    - Diagnostic modality
    - Tumor stage
    - Location and size of mass
- Assessment of Biliary Obstructive Symptoms
    - Right Upper Quadrant Pain
    - Fever/Chills
    - Jaundice
    - Itching
    - Dark urine
    - Pale stools
    - Nausea/Vomiting
- Laboratory Tests
    - Serum albumin level
    - Alkaline phosphatase
    - Total Bilirubin
    - Gamma GT
    - SGOT (AST—optional) and SGPT (ALT)
    - Normal Ranges

- Randomization into Group 1 or Group 2

7.4. *Stent Placement Procedure Visit (Group 2 subjects only)—Office Visit (may occur during Baseline visit)*

- Stent Details (including stent choice)
- Procedure Details (including sphincterotomy information)
- Adverse Events (AEs)

7.5. *Pre-Operative Visit (Week 2)—Telephone or Office Visit*

- Assessment of Biliary Obstructive Symptoms
    - Right Upper Quadrant Pain
    - Fever/Chills
    - Jaundice
    - Itching
    - Dark urine
    - Pale stools
    - Nausea/Vomiting
- Laboratory Tests
    - Serum albumin level
    - Alkaline phosphatase
    - Total Bilirubin
    - Gamma GT
    - SGOT (AST—optional) and SGPT (ALT)
- AEs

7.6. *Biliary Reintervention Visit (Group 1 or 2) —as needed*

- Timing
- Reason for Biliary Reintervention
- Type of Biliary Reintervention (including SEMS placement or removal)
- Adverse Events (AEs)

7.7. *Curative Intent Surgery—Office Visit (within 4 weeks, or delayed surgery by 120 days post-randomization)*

- Assessment of Biliary Obstructive Symptoms
    - Right Upper Quadrant Pain
    - Fever/Chills
    - Jaundice
    - Itching
    - Dark urine
    - Pale stools
    - Nausea/Vomiting
- Laboratory Tests
    - Serum albumin level
    - Alkaline phosphatase
    - Total Bilirubin
    - Gamma GT
    - SGOT (AST—optional) and SGPT (ALT)
- Operative details:
    - Exploration
    - Resection (including SEMS removal)
    - Reconstruction

- Blood Loss
- Intra and Post-Operative Transfusion
- Post-Operative Course
- Specimen Pathology (including margin status) per World Health Organization (WHO) classification system
- AEs

7.8. *Transition to Palliative Management Visit (if applicable)—Telephone or Office Visit*

- Assessment of Biliary Obstructive Symptoms
  - Right Upper Quadrant Pain
  - Fever/Chills
  - Jaundice
  - Itching
  - Dark urine
  - Pale stools
  - Nausea/Vomiting
- Laboratory Tests
  - Serum albumin level
  - Alkaline phosphatase
  - Total Bilirubin
  - Gamma GT
  - SGOT (AST—optional) and SGPT (ALT)
- AEs

7.9. *Post-Operative Follow-up Visit (30 days Post-Surgery, if applicable)—Telephone or Office Visit*

- Assessment of Biliary Obstructive Symptoms
  - Right Upper Quadrant Pain
  - Fever/Chills
  - Jaundice
  - Itching
  - Dark urine
  - Pale stools
  - Nausea/Vomiting
- Laboratory Tests
  - Serum albumin level
  - Alkaline phosphatase
  - Total Bilirubin
  - Gamma GT
  - SGOT (AST—optional) and SGPT (ALT)
- AEs

7.10. *Long Term Follow-up Visit (120 days—150 days post randomization)—Telephone or Office Visit*

- Assessment of Biliary Obstructive Symptoms
  - Right Upper Quadrant Pain
  - Fever/Chills
  - Jaundice
  - Itching
  - Dark urine
  - Pale stools
  - Nausea/Vomiting
- Laboratory Tests
  - Serum albumin level
  - Alkaline phosphatase
  - Total Bilirubin
  - Gamma GT
  - SGOT (AST—optional) and SGPT (ALT)
- AEs

7.11. *Study Completion*

Each subject will be followed for 120 days from the time of randomization. In cases where patients experience a delay of planned surgery, the long term follow up can be extended up to 150 days post randomization to allow for completion of a Follow-up Visit at 30 days after surgery.

A subject will be considered lost to follow-up if the subject remains unresponsive to communication after three documented attempts by study staff.

8. Statistical Considerations

8.1. *Hypothesis*

Statistical testing will be performed to determine if the rate of complications for the Pre-Operative Biliary Drainage with SEMS group is non-inferior to the No Pre-Operative Biliary Drainage group. The null hypothesis is that the complication rate is inferior in the Pre-Operative Biliary Drainage with SEMS versus the No Pre-Operative Biliary Drainage group:

Ho: $\pi_{test} - \pi_{control} \geq \Delta$ (Inferior)

Ha: $\pi_{test} - \pi_{control} < \Delta$ (Non-inferior)

where π_{test} and $\pi_{control}$ are the probabilities of having pre-operative, operative and peri-operative complications in the Pre-Operative Biliary Drainage with SEMS arm and No Pre-Operative Biliary Drainage arm respectively, and Δ is defined as the non-inferiority margin.

8.2. *Sample Size*

A literature search of preoperative biliary drainage with self-expanding metal stents in patients with pancreatic or periampullary cancer yielded 8 articles with 305 patients.[21-24, 27-31]

The following meta-analysis of the probability for pre-operative, operative and post-operative complications was done:

- A rate of 24.2% [95% CI: 13.2%, 37.2%] was calculated using the 8 articles.

The sample size was calculated for a one-sided 0.050 exact Farrington-Manning test using StatXact 9®. If the P value from the exact Farrington-Manning test is <0.05 then the *Pre-Operative Biliary Drainage with SEMS* group will be considered non-inferior to the *No Pre-Operative Biliary Drainage* group. The expected probability of complications in the *Pre-Operative Biliary Drainage with SEMS* arm and *No Pre-Operative Biliary Drainage* arm is 37.2%, which was taken from the upper limit of the 95% CI from the meta-analysis described above and from the only available Level 1 study comparing no drainage to preoperative stenting in which the complication rate was reported to be 39% in the no drainage arm.[9] The non-inferiority margin (Δ) is 15%. Given these assumptions and a one-sided 5% significance level, 2 x 132 = 264 subjects will provide 80% power to reject the null hypothesis, that the *Pre-Operative Biliary Drainage with SEMS* group is inferior to the *No Pre-Operative Biliary Drainage* group.

To compensate for possible loss of subjects after enrollment and complete assessment of inclusion/exclusion criteria, an additional 10% of subjects will be enrolled, for a total of 2 x 147 = 294 subjects.

8.3. *Analysis Populations*

8.3.1. Enrolled Cohort

A subject is considered “enrolled” after signing the study-specific ICF. Subjects who sign the ICF but subsequently do not meet one or more of the eligibility criteria provided in Section 5.2 and Section 5.3 will be considered screen failures and excluded from the study.

8.3.2. Intent-to-Treat Cohort

This cohort consists of those “enrolled” subjects who meet all inclusion/exclusion criteria and are subsequently randomized.

8.3.3. Per-Protocol Cohort

The per-protocol cohort is a subset of the ITT subjects who are treated per protocol after randomization (Group 1 no stent, and Group 2 received a study stent) and no major protocol deviations (ICH E9 definitions).

8.4. *Data Analyses*

All statistical analyses will be done using The SAS System software, version 8 or higher (Copyright © 2000 SAS Institute Inc., SAS Campus Drive, Cary, North Carolina 27513, USA. All rights reserved).
The distribution of prognostic factors between patients with and without data will be examined. Statistical models that account for censored data will be employed in appropriate circumstances, e.g. for time-to-event outcomes. Sensitivity analyses will be conducted to assess the impact of missing data on the interpretation of the results, e.g. a tipping point analysis.

8.4.1. Baseline Data

Baseline data will be summarized. Subject demographics, clinical history, risk factors, LFTs, tumor diagnosis, and obstruction symptoms will be summarized using descriptive statistics (e.g., mean, standard deviation, n, minimum, maximum) for continuous variables and frequency statistics for discrete variables.

8.4.2. Post-Procedure Endpoints

Post-procedure information will be collected at regularly scheduled follow-up examinations as detailed in the clinical study event schedule and will be summarized using descriptive statistics for continuous variables (e.g., mean, standard deviation, n, minimum, maximum) and frequency tables or proportions for discrete variables.

8.4.3. Subgroup Analyses

The subgroup analyses will include tabulating the primary endpoint and select secondary endpoints by gender.

8.4.4. Justification of Pooling

The analyses will be presented using pooled data across institutions. An analysis of the poolability will be made using logistic regression for binary outcomes, analysis of covariance for continuous outcomes, or proportional hazards regression for time-to-event outcomes, to assess differences between study institutions and to justify pooling data across institutions.

8.4.5. Multivariable Analyses

Univariate and multivariate analyses will be performed to assess possible predictors of the primary endpoint. Possible predictors may include any but not limited to demographic/baseline data and medical history data. Factors from the univariate model with $p \leq 0.20$ will also be modeled multivariately using a stepwise procedure in a generalized linear model or Cox Proportional Hazards model. The significance thresholds for entry and exit into the model will be set to $p<0.10$.

8.4.6. Changes to Planned Analyses

Any changes to the planned statistical analyses made prior to performing the analyses will be documented in a Statistical Analysis Plan approved prior to performing the analyses.

9. Potential Risks and Benefits

9.1. *Anticipated Adverse Device Effects with Use of WallFlex Biliary Stent*

As per the commercial DFU included with the study devices, the potential complications associated with the use of the WallFlex Biliary FC and UC Stent may include, but are not limited to:

- Pain
- Bleeding
- Fever
- Nausea
- Vomiting
- Infection
- Inflammation
- Recurrent obstructive jaundice
- Stent occlusion
- Tumor overgrowth around ends of stent
- Tumor ingrowth through the stent
- Mucosal hyperplasia
- Cholangitis
- Cholecystitis*
- Pancreatitis
- Bile duct ulceration
- Perforation of duodenum or bile duct
- Stent migration
- Death (other than that due to normal disease progression)
- Stent misplacement
- Perforation of the gallbladder due to the stent covering the cystic duct*
  *Note: In a small clinical trial of this device, two out of four (50%) subjects who had a stent placed across the cystic duct developed cholecystitis. One of these subjects suffered a perforated gallbladder due to the stent covering the cystic duct, requiring a drain to be placed.

As per the commercial DFU included with the study devices, potential complications associated with stent removal during an ERCP procedure include, but are not limited to:

- Pain
- Bleeding
- Fever
- Nausea

- Vomiting
- Infection
- Inflammation
- Recurrent obstructive jaundice
- Mucosal hyperplasia
- Cholangitis
- Cholecystitis
- Pancreatitis
- Ulceration of duodenum or bile duct
- Perforation of duodenum or bile duct
- Death (other than that due to normal disease progression)
- Impaction to the common bile duct wall

9.2. *Anticipated Adverse Events in Patients Without Pre-Operative Biliary Drainage*

- Severe jaundice
- Cholangitis

9.3. *Anticipated Surgical Adverse Events*

- Pancreaticojejunostomy leakage
- Delayed gastric emptying
- Biliary leakage
- Gastro/-duodenojejunostomy leakage
- Intra-abdominal abscess formation
- Wound infection
- Portal Vein Thrombosis
- Cholangitis
- Hemorrhage
- (Emergency) (re)laparotomy
- Pneumonia
- Myocardial infarction
- Mortality

9.4. *Risk Minimization Actions*

Additional risks may exist. Risks can be minimized through compliance with this protocol, performing procedures in the appropriate hospital environment, adherence to subject selection criteria, close monitoring of the subject's physiologic status during research procedures and/or follow-ups and by promptly supplying BSC with all pertinent information required by this protocol. The latter may facilitate inter-center communications regarding serious AEs.

9.5. *Anticipated Benefits*

Subjects may not receive any benefit from participating in this study but this study may provide a future benefit to medical science and other patients. To date there is no broadly accepted standard of practice pertaining to pre-operative drainage of patients as defined by the inclusion and exclusion criteria of the study.

9.6. *Risk to Benefit Rationale*

Based on prior BSC's clinical studies and collected reports in literature to-date, the risk-to-benefit ratio is within reason for forseeable risks. However, literature reports do not always capture all side effects. Observation and follow up of subjects is required as outlined in the protocol.

10. Safety Reporting

10.1. *Definitions and Classification*

Adverse event definitions are provided in Table 10.1-1.

Table 10.1-1: Adverse Event Definitions

Term	Definition
Adverse Event (AE)<br><br>*Ref: ISO 14155-2011*	Any untoward medical occurrence, unintended disease or injury, or untoward clinical signs (including abnormal lab findings) in subjects, users or other persons, whether or not related to the investigational medical device. This includes events related to:<br>• The investigational medical device or comparator<br>• The procedures involved (study-required)<br>For users/other persons, this definition is restricted to events related to the investigational device
Adverse Device Effect (ADE)<br><br>*Ref: ISO 14155-2011*	Adverse event related to the use of an investigational medical device:<br>• This includes adverse events resulting from insufficient or inadequate instructions for the use, deployment, implantation, installation or operation, or any malfunction of the investigational medical device.<br>• This includes any event resulting from use error or from intentional misuse of the investigational medical device.
Serious Adverse Event (SAE)<br><br>*Ref: ISO 14155-2011*	An adverse event that:<br>• Led to death<br>• Led to a serious deterioration in the health of the subject that either resulted in:

Table 10.1-1: Adverse Event Definitions

Term	Definition
	○ a life-threatening illness or injury, or<br>○ a permanent impairment of a body structure or a body function, or<br>○ in-patient hospitalization or prolonged hospitalization (of an existing hospitalization), or<br>○ medical or surgical intervention to prevent life-threatening illness or injury or permanent impairment to a body structure or a body function<br>• Led to fetal distress, fetal death, or a congenital abnormality or birth defect.<br>**Note**: Planned hospitalization for a pre-existing condition, or a procedure required by the protocol, without serious deterioration in health, is not considered a serious adverse event.<br>**Note**: For SAE reporting requirements see the information below for SADE.
Serious Adverse Device Effect (SADE)<br><br>*Ref: ISO 14155-2011*	An adverse device effect that has resulted in any of the consequences characteristic of a serious adverse event.<br>**Note**: All SAEs that could have led to a SADE if suitable action had not been taken or if circumstances had been less fortunate shall be reported as required by the local IRB/EC, national regulations, or the protocol. If applicable, see MEDDEV 2.7/3 12/2010 for reporting timeline requirements.
Device Deficiency<br><br>*Ref: ISO 14155-2011*<br><br>*Ref: MEDDEV 2.7/3 12/2010*	A device deficiency is any inadequacy of a medical device with respect to its identity, quality, durability, reliability, safety or performance.<br>**Note**: Device deficiencies include malfunctions, use errors, and inadequate labeling.<br>**Note**: All device deficiencies that could have led to a SADE if suitable action had not been taken or if circumstances had been less fortunate shall be reported as required by the local IRB/EC, national regulations, or the protocol. If applicable, see MEDDEV 2.7/3 12/2010 for reporting timeline requirements.

Abbreviations: EC=Ethics Committee; IRB=Institutional Review Board

Underlying diseases are not reported as AEs unless there is an increase in severity or frequency during the course of the investigation. Death should not be recorded as an AE, but should only be reflected as an outcome of a specific SAE (see Table 10.1-1 for AE definitions).

Any AE experienced by the study subject after informed consent, whether during or subsequent to the procedure, must be recorded in the eCRF.

Refer to Section 9 for the known risks associated with the study device(s).

Adverse events that occur during the course of the study will be evaluated against a list of pre-specified complication criteria as defined in Section 12.14. Determination of whether an AE meets the definition of a pre-specified complication will be made by study physicians.

10.2. *Relationship to Study Device(s)*

The Investigator must assess the relationship of the AE to the study device as related or unrelated. See criteria in Table 10.2-2:

Table 10.2-2: Criteria for Assessing Relationship of Study Device to Adverse Event

**Classification**	**Description**
**Unrelated**	The adverse event is determined to be due to a concurrent illness or effect of another device/drug and is not related to the investigational product.
**Related**	• The adverse event is determined to be potentially related to the investigational product, and an alternative etiology is equally or less likely compared to the potential relationship to investigational product.<br>• There is a strong relationship to investigational product, or recurs on re-challenge, and another etiology is unlikely.<br>• There is no other reasonable medical explanation for the event.

10.3. *Investigator Reporting Requirements*

Investigators will be required to report all SAEs and ADEs.

10.3.1. Serious Adverse Events

These events should be reported to the Global Safety Office and/or Project Manager **within 2 business days** of first becoming aware of the event. Events should be documented in the eCRF and all relevant source documentation for the event should be provided to the Global Safety Office, as applicable.

10.3.2. Adverse Events

Device-related events should be reported to the Global Safety Office and/or Project Manager **within 10 business days** of first becoming aware of the event. Unrelated AEs will not be collected.

10.3.3. Device Failures, Malfunctions, and Product Nonconformities

These events should be reported to Project Manager and/or Global Safety Office **within 1 business day** of first becoming aware of the event. Events should be documented in the eCRF.

10.4. *Boston Scientific Device Deficiencies*

All device deficiencies (including but not limited to failures, malfunctions, use errors, product nonconformities, and labeling errors) will be documented and reported to BSC. If possible, the device(s) should be returned to BSC for analysis. Instructions for returning the investigational device(s) will be provided. If it is not possible to return the device, the investigator should document why the device was not returned and the final disposition of the device. Device failures and malfunctions should also be documented in the subject's medical record.

Device deficiencies, failures, malfunctions, and product nonconformities are not to be reported as adverse events. However, if there is an adverse event that results from a device failure or malfunction, that specific event would be recorded on the appropriate eCRF.

10.5. *Reporting to Regulatory Authorities / IRBs / ECs / Investigators*

BSC is responsible for reporting adverse event information to all participating investigators and regulatory authorities, as applicable.

The Principal Investigator is responsible for informing the IRB/EC and regulatory authorities of SAEs as required by local procedure.

11. Bibliography

1. Decker C, Christein JD, Phadnis MA, Wilcox CM, Varadarajulu S. Biliary metal stents are superior to plastic stents for preoperative biliary decompression in pancreatic cancer. Surg Endosc. 2011 Jul;25(7):2364-7. Epub 2011 Mar 4.
2. Wasan SM, Ross WA, Staerkel GA, Lee GH. Use of Expandable Metallic Biliary Stents in Resectable Pancreatic Cancer. Am J Gastroenterol. 2005;100:2056–2061.
3. Mullen JT, Lee JH, Gomez HF, Ross WA, et al. Pancreaticoduodenectomy After Placement of Endobiliary Metal Stents. J Gastrointest Surg 2005;9:1094–1105.
4. Sanders MK, El Hajj II, Slivka A, et al Placement of Self-Expanding Metal Biliary Stents (SEMS) in Patients with Resectable Pancreatic Cancer [Abstract]. Gastrointest Endosc 2009; 69(5)
5. Kahaleh M, Brock A, Conaway MR, et al. Covered self−expandable metal stents in pancreatic malignancy regardless of resectability: a new concept validated by a decision analysis. Endoscopy 2007 Apr;39(4):319-24
6. Pop GH, Richter JA, Sauer B, et al. Bridge to surgery using partially covered self-expandable metal stents (PCMS) in malignant biliary stricture: an acceptable paradigm? Surg Endosc 2011 Feb;25(2):613-8

7. Lawrence C , Howell DA, Conklin DE, et al. Delayed pancreaticoduodenectomy for cancer patients with prior ERCP-placed, nonforeshortening, self-expanding metal stents: a positive outcome, Gastrointest Endosc 2006 May;63 (6): 804-807
8. Singal AK, Ross WA, Guturu P, et al. Self-Expanding Metal Stents for Biliary Drainage in Patients with Resectable Pancreatic Cancer: Single-Center Experience with 79 Cases. Dig Dis Sci 2011 Jul 13. [Epub ahead of print]
9. van der Gaag NA, Rauws EAJ, van Eijck CHJ, et al. Preoperative Biliary Drainage for Cancer of the Head of the Pancreas. N Engl J Med 2010;362:129-37.
10. Hariharan D, Saied A, Kocher HM. Analysis of mortality rates for pancreatic cancer across the world. HPB (Oxford). 2008; 10(1): 58–62.
11. American Cancer Society. Cancer Facts and Figures 2012. Atlanta: American Cancer Society; 2012.
12. NCCN Clinical Guidelines in Oncology Pancreatic Adenocarcinoma Version 2.2011, NCCN.org
13. Callery MP, Chang KJ, Fishman EK, Talamonti MS, William Traverso L, Linehan DC. Pretreatment assessment of resectable and borderline resectable pancreatic cancer: expert consensus statement. Ann Surg Oncol. 2009 Jul;16(7):1727-33. Epub 2009 Apr 24.
14. Wong JC, Lu DS. Staging of pancreatic adenocarcinoma by imaging studies. Clin Gastroenterol Hepatol. 2008 Dec;6(12):1301-8. Epub 2008 Sep 27. Review.
15. House MG, Yeo CJ, Cameron JL, Campbell KA, Schulick RD, Leach SD, Hruban RH, Horton KM, Fishman EK, Lillemoe KD. Predicting resectability of periampullary cancer with three-dimensional computed tomography. J Gastrointest Surg. 2004 Mar-Apr;8(3):280-8.
16. Olivié D, Lepanto L, Billiard JS, Audet P, Lavallée JM. Predicting resectability of pancreatic head cancer with multi-detector CT. Surgical and pathologic correlation. JOP. 2007 Nov 9;8(6):753-8.
17. Schima W, Ba-Ssalamah A, Goetzinger P, Scharitzer M, Koelblinger C. State-of-the-art magnetic resonance imaging of pancreatic cancer. Top Magn Reson Imaging. 2007 Dec;18(6):421-9. Review.
18. NCCN Clinical Guidelines in Oncology Pancreatic Adenocarcinoma Version 2.2012, NCCN.org
19. Rösch T, Braig C, Gain T, Feuerbach S, Siewert JR, Schusdziarra V, Classen M. Staging of pancreatic and ampullary carcinoma by endoscopic ultrasonography. Comparison with conventional sonography, computed tomography, and angiography. Gastroenterology. 1992 Jan;102(1):188-99.
20. Varadarajulu S, Wallace MB. Applications of endoscopic ultrasonography in pancreatic cancer. Cancer Control. 2004 Jan-Feb;11(1):15-22. Review.

21. Ahmed SI, Bochkarev V, Oleynikov D, Sasson AR. Patients with pancreatic adenocarcinoma benefit from staging laparoscopy. J Laparoendosc Adv Surg Tech A. 2006 Oct;16(5):458-63.
22. Warshaw AL, Gu ZY, Wittenberg J, Waltman AC. Preoperative staging and assessment of resectability of pancreatic cancer. Arch Surg. 1990 Feb;125(2):230-3.
23. Katz MH, Hwang R, Fleming JB, Evans DB. Tumor-node-metastasis staging of pancreatic adenocarcinoma. CA Cancer J Clin. 2008 Mar-Apr;58(2):111-25. Epub 2008 Feb 13. Review.
24. Edge SB, Byrd DR, Compton CC, et al., eds. Exocrine and endocrine pancreas. AJCC Cancer Staging Manual. 7th ed. New York, NY: Springer, 2010, pp 241-9.
25. Tseng JF, Tamm EP, Lee JE, et al. Venous resection in pancreatic cancer surgery. Best Pract Res Clin Gastroenterol. 2006;20(2):349–364.
26. Bonin EA, Baron TH. Preoperative biliary stents in pancreatic cancer. J Hepatobiliary Pancreat Sci. 2011 Sep;18(5):621-9. Review.
27. Li D, Xie K, Wolff R, Abbruzzese JL. Pancreatic cancer. The Lancet 2004; 363 (9414):1049-1057.
28. Gumbs AA, Rodriguez Rivera AM, Milone L, Hoffman JP. Laparoscopic pancreatoduodenectomy: a review of 285 published cases. Ann Surg Oncol. 2011 May;18(5):1335-41. Epub 2011 Jan 5. Review.
29. Hidalgo M. Pancreatic cancer. N Engl J Med. 2010;362:1605–1617.
30. Boulay BR, Gardner TB, Gordon SR. Occlusion rate and complications of plastic biliary stent placement in patients undergoing neoadjuvant chemoradiotherapy for pancreatic cancer with malignant biliary obstruction. J Clin Gastroenterol 2010 Jul;44(6):452-5.
31. Moss AC, Morris E, Leyden J, MacMathuna P. Malignant distal biliary obstruction: a systematic review and meta-analysis of endoscopic and surgical bypass results. Cancer Treat Rev. 2007 Apr;33(2):213-21. Epub 2006 Dec 8. Review.

12. Appendices: Sponsor Required Protocol Sections

12.1. *Data Management*

12.1.1. Data Collection, Processing, and Review

Subject data will be recorded in a limited access secure electronic data capture (EDC) system.

The clinical database will reside on a production server. All changes made to the clinical data will be captured in an electronic audit trail and available for review by Boston Scientific Corporation (BSC) or its representative. The associated software and database have been designed to meet regulatory compliance for deployment as part of a validated system compliant with laws and regulations applicable to the conduct of clinical studies pertaining to the use of electronic records and signatures. Database backups are performed regularly.

The Investigator provides his/her electronic signature on the appropriate electronic case report forms (eCRFs) in compliance with local regulations. A written signature on printouts of the eCRFs must also be provided if required by local regulation. Changes to data previously submitted to the sponsor require a new electronic signature by the Investigator acknowledging and approving the changes.

Visual and/or electronic data review will be performed to identify possible data discrepancies. Manual and/or automatic queries will be created in the EDC system and will be issued to the site for appropriate response. Site staff will be responsible for resolving all queries in the database.

12.1.2. Data Retention

The Investigator will maintain, at the investigative site, in original format all essential study documents and source documentation that support the data collected on the study subjects in compliance with ICH/GCP guidelines. Documents must be retained for at least 2 years after the last approval of a marketing application or until at least 2 years have elapsed since the formal discontinuation of the clinical investigation of the product. These documents will be retained for a longer period of time by agreement with BSC or in compliance with other local regulations. It is BSC's responsibility to inform the Investigator when these documents no longer need to be maintained. The Investigator will take measures to ensure that these essential documents are not accidentally damaged or destroyed. If for any reason the Investigator withdraws responsibility for maintaining these essential documents, custody must be transferred to an individual who will assume responsibility and BSC must receive written notification of this custodial change.

12.2. *Amendments*

If a protocol revision is necessary which affects the rights, safety or welfare of the subject or scientific integrity of the data, an amendment is required. Appropriate approvals (e.g., IRB/EC/FDA/CA) of the revised protocol must be obtained prior to implementation.

12.3. *Deviations*

An Investigator must not make any changes or deviate from this protocol, except to protect the life and physical well-being of a subject in an emergency. An investigator shall notify the sponsor and the reviewing IRB/EC of any deviation from the investigational plan to protect the life or physical well-being of a subject in an emergency, and those deviations which affect the scientific integrity of the clinical investigation. Such notice shall be given as soon as possible, but no later than 5 working days after the emergency occurred, or per prevailing local requirements, if sooner than 5 working days.

All deviations from the investigational plan, with the reason for the deviation and the date of occurrence, must be documented and reported to the sponsor using the Protocol Deviation EDC CRF. Sites may also be required to report deviations to the IRB/EC, per local guidelines and government regulations.

Deviations will be reviewed and evaluated on an ongoing basis and, as necessary, appropriate corrective and preventive actions (including notification, center re-training, or discontinuation) will be put into place by the sponsor.

12.4. *Device/Equipment Accountability*

There are no investigational devices used in this study. The WallFlex Biliary Fully Covered and Uncovered Stent Systems are available for commercial use in the geographic areas in which this clinical study is taking place; therefore, there is no requirement for device accountability for the purposes of this study. Device lot information must be maintained in the subject's medical record and recorded on the appropriate case report form.

Any individual country/region requirements that depart from the aforementioned will be implemented on a case-by-case basis.

12.5. *Compliance*

12.5.1. Statement of Compliance

This study will be conducted in accordance with relevant sections of the International Standard (ISO) 14155: Clinical Investigation of Medical devices for Human Subjects – Good Clinical Practice, the relevant parts of the ICH Guidelines for Good Clinical Practices, ethical principles that have their origins in the Declaration of Helsinki, and pertinent individual country laws and regulations. The study will not begin until the required approval/favorable opinion from the EC and/or regulatory authority has been obtained, if appropriate. Any additional requirements imposed by the EC or regulatory authority shall be followed, if appropriate.

12.5.2. Investigator Responsibilities

The Principal Investigator of an investigational center is responsible for ensuring that the study is conducted in accordance with the Clinical Study Agreement, the investigational plan/protocol, ISO 14155, ethical principles that have their origins in the Declaration of Helsinki, any conditions of approval imposed by the reviewing IRB/EC, and prevailing local and/or country laws and/or regulations, whichever affords the greater protection to the subject.

The Principal Investigator's responsibilities include, but are not limited to, the following.

- Prior to beginning the study, sign the Investigator Agreement and Protocol Signature page documenting his/her agreement to conduct the study in accordance with the protocol.
- Provide his/her qualifications and experience to assume responsibility for the proper conduct of the study and that of key members of the center team through up-to-date curriculum vitae or other relevant documentation and disclose potential conflicts of interest, including financial, that may interfere with the conduct of the clinical study or interpretation of results.

- Make no changes in or deviate from this protocol, except to protect the life and physical well-being of a subject in an emergency; document and explain any deviation from the approved protocol that occurred during the course of the clinical investigation.
- Create and maintain source documents throughout the clinical study and ensure their availability with direct access during monitoring visits or audits; ensure that all clinical-investigation-related records are retained per requirements.
- Ensure the accuracy, completeness, legibility, and timeliness of the data reported to the sponsor in the CRFs and in all required reports.
- Record, report, and assess (seriousness and relationship to the device/procedure) every adverse event and observed device deficiency.
- Report to BSC, per the protocol requirements, all SAEs and device deficiencies that could have led to a SADE.
- Report to the IRB/EC and regulatory authorities any SAEs and device deficiencies that could have led to a SADE, if required by the national regulations or this protocol or by the IRB/EC, and supply BSC with any additional requested information related to the safety reporting of a particular event.
- Maintain the device accountability records and control of the device, ensuring that the investigational device is used only by authorized/designated users and in accordance with this protocol and instructions/directions for use.
- Allow the sponsor to perform monitoring and auditing activities, and be accessible to the monitor and respond to questions during monitoring visits.
- Allow and support regulatory authorities and the IRB/EC when performing auditing activities.
- Ensure that informed consent is obtained in accordance with this protocol and local IRB/EC requirements.
- Provide adequate medical care to a subject during and after a subject's participation in a clinical study in the case of adverse events, as described in the Informed Consent Form (ICF).
- Inform the subject of the nature and possible cause of any adverse events experienced.
- As applicable, provide the subject with necessary instructions on proper use, handling, storage, and return of the investigational device when it is used/operated by the subject.
- Inform the subject of any new significant findings occurring during the clinical investigation, including the need for additional medical care that may be required.
- Provide the subject with well-defined procedures for possible emergency situations related to the clinical study, and make the necessary arrangements for emergency treatment, including decoding procedures for blinded/masked clinical investigations, as needed.
- Ensure that clinical medical records are clearly marked to indicate that the subject is enrolled in this clinical study.

- Ensure that, if appropriate, subjects enrolled in the clinical investigation are provided with some means of showing their participation in the clinical investigation, together with identification and compliance information for concomitant treatment measures (contact address and telephone numbers shall be provided).
- Inform, with the subject's approval or when required by national regulations, the subject's personal physician about the subject's participation in the clinical investigation.
- Make all reasonable efforts to ascertain the reason(s) for a subject's premature withdrawal from clinical investigation while fully respecting the subject's rights.
- Ensure that an adequate investigation site team and facilities exist and are maintained and documented during the clinical investigation.
- Ensure that maintenance and calibration of the equipment relevant for the assessment of the clinical investigation is appropriately performed and documented, where applicable.

12.5.3. Delegation of Responsibility

When specific tasks are delegated by an investigator, included but not limited to conducting the informed consent process, the investigator is responsible for providing appropriate training and adequate supervision of those to whom tasks are delegated. The investigator is accountable for regulatory violations resulting from failure to adequately supervise the conduct of the clinical study.

12.6. *Institutional Review Board/ Ethics Committee*

Prior to gaining Approval-to-Enroll status, the investigational center will provide to the sponsor documentation verifying that their IRB/EC is registered or that registration has been submitted to the appropriate agency, as applicable according to national/regulatory requirements.

A copy of the written IRB/EC and/or competent authority approval of the protocol (or permission to conduct the study) and Informed Consent Form, must be received by the sponsor before recruitment of subjects into the study and shipment of investigational product/equipment. Prior approval must also be obtained for other materials related to subject recruitment or which will be provided to the subject.

Annual IRB/EC approval and renewals will be obtained throughout the duration of the study as required by local/country or IRB/EC requirements. Copies of the Investigator's reports and the IRB/EC continuance of approval must be provided to the sponsor.

12.7. *Sponsor Responsibilities*

All information and data sent to BSC concerning subjects or their participation in this study will be considered confidential by BSC. Only authorized BSC personnel or a BSC representative will have access to these confidential records. Authorized regulatory personnel have the right to inspect and copy all records pertinent to this study. Study data collected during this study may be used by BSC for the purposes of this study, publication, and to

support future research and/or other business purposes. All data used in the analysis and reporting of this study will be without identifiable reference to specific subject name.

Boston Scientific will keep subjects' health information confidential in accordance with all applicable laws and regulations. Boston Scientific may use subjects' health information to conduct this research, as well as for additional purposes, such as overseeing and improving the performance of its device, new medical research and proposals for developing new medical products or procedures, and other business purposes. Information received during the study will not be used to market to subjects; subject names will not be placed on any mailing lists or sold to anyone for marketing purposes.

12.8. *Insurance*

Where required by local/country regulation, proof and type of insurance coverage, by BSC for subjects in the study will be obtained.

12.9. *Monitoring*

Monitoring will be performed during the study to assess continued compliance with the protocol and applicable regulations. In addition, the monitor verifies that study records are adequately maintained, that data are reported in a satisfactory manner with respect to timeliness, adequacy, and accuracy and that the Investigator continues to have sufficient staff and facilities to conduct the study safely and effectively. The Investigator/institution guarantees direct access to original source documents by BSC personnel, their designees and appropriate regulatory authorities.

The study may also be subject to a quality assurance audit by BSC or its designees, as well as inspection by appropriate regulatory authorities. It is important that the Investigator and relevant study personnel are available during on-site monitoring visits or audits and that sufficient time is devoted to the process.

12.10. *Informed Consent*

Subject participation in this clinical study is voluntary. Informed Consent is required from all subjects or their legally authorized representative. The Investigator is responsible for ensuring that Informed Consent is obtained prior to the use of any investigational devices, study-required procedures and/or testing, or data collection.

The obtaining and documentation of Informed Consent must be in accordance with the principles of the Declaration of Helsinki, ISO 14155, any applicable national regulations, and local Ethics Committee and/or Regulatory authority body, as applicable. The ICF must be approved by the center's IRB/EC, or central IRB, if applicable.

Boston Scientific will provide a study-specific template of the ICF to investigators participating in this study. The ICF template may be modified to meet the requirements of the investigative center's IRB/EC. Any modification requires approval from BSC prior to use of the form. The ICF must be in a language understandable to the subject and if needed, BSC will assist the center in obtaining a written consent translation. Translated consent forms must also have IRB/EC approval prior to their use. Privacy language shall be included in the body of the form or as a separate form as applicable.

The process of obtaining Informed Consent shall:

- be conducted by the Principal Investigator or designee authorized to conduct the process,
- include a description of all aspects of the clinical study that are relevant to the subject's decision to participate throughout the clinical study,
- avoid any coercion of or undue influence of subjects to participate,
- not waive or appear to waive subject's legal rights,
- use native language that is non-technical and understandable to the subject or his/her legal representative,
- provide ample time for the subject to consider participation and ask questions if necessary,
- ensure important new information is provided to new and existing subjects throughout the clinical study.

The ICF shall always be signed and personally dated by the subject or legal representative and by the investigator or an authorized designee responsible for conducting the informed consent process. If a legal representative signs, the subject shall be asked to provide informed consent for continued participation as soon as his/her medical condition allows. The original signed ICF will be retained by the center and a copy of the signed and dated document and any other written information must be given to the person signing the form.

Failure to obtain subject consent will be reported by BSC to the applicable regulatory body according to their requirements (e.g., FDA requirement is within 5 working days of learning of such an event). Any violations of the informed consent process must be reported as deviations to the sponsor and local regulatory authorities (e.g. IRB/EC), as appropriate.

If new information becomes available that can significantly affect a subject's future health and medical care, that information shall be provided to the affected subject(s) in written form via a revised ICF or, in some situations, enrolled subjects may be requested to sign and date an addendum to the ICF. In addition to new significant information during the course of a study, other situations may necessitate revision of the ICF, such as if there are amendments to the protocol, a change in Principal Investigator, administrative changes, or following annual review by the IRB/EC. The new version of the ICF must be approved by the IRB/EC. Boston Scientific approval is required if changes to the revised ICF are requested by the center's IRB/EC. The IRB/EC will determine the subject population to be re-consented.

12.11. *Committees*

12.11.1. Safety Monitoring Process

An independent data review (IDR) board comprised of physician experts in surgery and gastroenterological endoscopy not participating in the clinical study and with no affiliation with BSC, will provide external oversight and review for potential safety concerns.

During the course of the clinical study, the IDR board will periodically review aggregate accumulating safety data to monitor for the incidence of major adverse events and other trends.

Any IDR recommendations for clinical study modification or termination because of concerns over subject safety or issues relating to data monitoring or quality control will be submitted in writing to BSC for consideration.

12.12. *Suspension or Termination*

12.12.1. Premature Termination of the Study

Boston Scientific Corporation reserves the right to terminate the study at any stage but intends to exercise this right only for valid scientific or administrative reasons and reasons related to protection of subjects. Investigators, associated IRBs/ECs, and regulatory authorities, as applicable, will be notified in writing in the event of study termination.

12.12.2. Criteria for Premature Termination of the Study

Possible reasons for premature study termination include, but are not limited to, the following.

- The occurrence of unanticipated adverse device effects that present a significant or unreasonable risk to subjects enrolled in the study.
- An enrollment rate far below expectation that prejudices the conclusion of the study.
- A decision on the part of Boston Scientific to suspend or discontinue development of the device.

12.12.3. Termination of Study Participation by the Investigator or Withdrawal of IRB/ EC Approval

Any investigator, or IRB/ EC in the Preoperative Biliary Drainage RCT Study may discontinue participation in the study or withdrawal approval of the study, respectively, with suitable written notice to Boston Scientific. Investigators, associated IRBs/ECs, and regulatory authorities, as applicable, will be notified in writing in the event of these occurrences.

12.12.4. Requirements for Documentation and Subject Follow-up

In the event of premature study termination a written statement as to why the premature termination has occurred will be provided to all participating centers by Boston Scientific. The IRB/EC and regulatory authorities, as applicable, will be notified. Detailed information on how enrolled subjects will be managed thereafter will be provided.

In the event an IRB or EC terminates participation in the study, participating investigators, associated IRBs/ECs, and regulatory authorities, as applicable, will be notified in writing. Detailed information on how enrolled subjects will be managed thereafter will be provided by Boston Scientific.

In the event an investigator terminates participation in the study, study responsibility will be transferred to a co-investigator, if possible. In the event there are no opportunities to transfer

investigator responsibility; detailed information on how enrolled subjects will be managed thereafter will be provided by Boston Scientific.

The investigator must return all documents and investigational product to Boston Scientific, unless this action would jeopardize the rights, safety, or welfare of the subjects.

12.12.5. Criteria for Suspending/Terminating a Study Center

Boston Scientific Corporation reserves the right to stop the inclusion of subjects at a study center at any time after the study initiation visit if no subjects have been enrolled for a period beyond 2 months after center initiation, or if the center has multiple or severe protocol violations/noncompliance without justification and/or fails to follow remedial actions.

In the event of termination of investigator participation, all study devices and testing equipment, as applicable, will be returned to BSC unless this action would jeopardize the rights, safety or well-being of the subjects. The IRB/EC and regulatory authorities, as applicable, should be notified. All subjects enrolled in the study at the center will continue to be followed per protocol. The Principal Investigator at the center must make provision for these follow-up visits unless BSC notifies the investigational center otherwise.

12.13. *Publication Policy*

In accordance with the Corporate Policy on the Conduct of Human Subject Research, BSC requires disclosure of its involvement as a sponsor or financial supporter in any publication or presentation relating to a BSC study or its results. In accordance with the Corporate Policy for the Conduct of Human Subject Research, BSC will submit study results for publication (regardless of study outcome) following the conclusion or termination of the study. Boston Scientific Corporation adheres to the Contributorship Criteria set forth in the Uniform Requirements of the International Committee of Medical Journal Editors (ICMJE; http://www.icmje.org). In order to ensure the public disclosure of study results in a timely manner, while maintaining an unbiased presentation of study outcomes, BSC personnel may assist authors and investigators in publication preparation provided the following guidelines are followed.

- All authorship and contributorship requirements as described above must be followed.
- BSC involvement in the publication preparation and the BSC Publication Policy should be discussed with the Coordinating Principal Investigator(s) and/or Executive/Steering Committee at the onset of the project.
- The First and Senior authors are the primary drivers of decisions regarding publication content, review, approval, and submission.

12.14. *Definitions of complication criteria (per van der Gaag article):*

<u>Specific PBD (ERCP, PTC) related:</u>

- o **Acute pancreatitis** Abdominal pain and a serum concentration of pancreatic enzymes (amylase or lipase) three or more times the upper limit of normal, that required more than one night of hospitalization

- **Acute cholecystitis** No suggestive clinical or radiographic signs of acute cholecystitis before the procedure and if emergency cholecystectomy is subsequently required
- **Perforation** Retroperitoneal or bowel-wall perforation documented by any radiographic technique or direct visual evidence
- **Stent Occlusion** Recurring obstructive jaundice with necessary stent replacement

Specific surgery related:

- **Pancreaticojejunostomy leakage** Drain output of any measurable volume of fluid on or after postoperative day 3 with an amylase content greater than 3 times the serum amylase activity, graded according to clinical course (ISGPS grade A, B, C), or direct visual evidence of defect at anastomosis
- **Delayed gastric emptying** Gastric stasis requiring nasogastric intubation for 10 days or more, or the inability to tolerate a regular (solid) diet on or before the fourteenth postoperative day, not due to sequelae of intra-abdominal complications (i.e. abscess, anastomotic leakage)
- **Biliary leakage** Bilirubin in abdominal drain or dehiscence found at laparotomy
- **Gastro/-duodenojejunostomy leakage** Conclusive radiographic or direct visual evidence of a defect of the anastomosis
- **Intra-abdominal abscess formation** Intra-abdominal fluid collection with positive cultures identified by ultrasonography or computed tomography, associated with persistent fever and elevations of white blood cells
- **Wound infection** Requiring intervention otherwise considered as minor complication
- **Portal Vein Thrombosis** Conclusive radiologic evidence of thrombosis

Following either procedure:

- **Cholangitis** Elevation in temperature more than 38°C, thought to have a biliary cause, without concomitant evidence of acute cholecystitis, requiring intervention
- **Hemorrhage** Bleeding after the index procedure requiring transfusion of ≥4 units of packed cells within a 24-hour period, or leading to relaparotomy/intervention
- **(Emergency) (re)laparotomy** Any (other) reason following either preoperative biliary drainage or another surgical procedure
- **Pneumonia** Pulmonary infection with radiological confirmation and requiring antibiotic treatment
- **Mortality** In-hospital death, due to protocol complications or any cause, including progression of disease, within the study period

Final protocol

Randomized Multi-Center Study Comparing No Drainage to Preoperative Biliary Drainage Using Metal Stents in Patients with Resectable Pancreatic or Periampullary Cancer

Preoperative Biliary Drainage RCT

CLINICAL PROTOCOL

E7059

Sponsored By

Boston Scientific Corporation
100 Boston Scientific Way
Marlborough, MA 01752-1234
USA

This protocol contains confidential information for use by the Investigators and their designated representatives participating in this clinical investigation. It should be held confidential and maintained in a secure location.

Do not copy or distribute without written permission from Boston Scientific Corporation.

Contact Information

Role	Contact
**Clinical Contact**	Lina Ginnetti<br>Clinical Project Manager<br>Boston Scientific Corporation<br>100 Boston Scientific Way<br>Marlborough, MA 01752-1234<br>USA
**Coordinating Principal Investigators**	Professor Guido Costamagna, MD<br>Università Cattolica del Sacro Cuore<br>Policlinico A. Gemelli<br>U.O. di Endoscopia Digestiva Chirurgica<br>Largo A. Gemelli, 8<br>00168 Rome, Italy<br><br>Professor James Lau, MD<br>Prince of Wales Hospital<br>30-32 Ngan Shing Street<br>Shatin<br>New Territories, Hong Kong

Original Release: May 16, 2012

Current Version: October 20, 2014

Do not copy or distribute without written permission.

Investigator's Signature Page

STUDY TITLE: Randomized Multi-Center Study Comparing No Drainage to Preoperative Biliary Drainage Using Metal Stents in Patients with Resectable Pancreatic or Periampullary Cancer

STUDY CENTER: ____

(Print name of study center)

PROTOCOL VERSION: AD

We, the undersigned, have read and understand the protocol specified above and agree on its content. We agree to perform and conduct the study as described in the protocol. In addition, when applicable, we agree to enlist sub-investigators who also agree to perform and conduct the study as described in the protocol.

____ ______________________

Principal Investigator Date

Print name:

____ ______________________

Co-Principal Investigator (if applicable) Date

Print name:

Protocol Synopsis

**Full Title**	**Randomized Multi-Center Study Comparing No Drainage to Preoperative Biliary Drainage Using Metal Stents in Patients with Resectable Pancreatic or Periampullary Cancer**
**Short Title**	Preoperative Biliary Drainage RCT
**Objective(s)**	To demonstrate that preoperative biliary drainage using self-expanding metal stents (SEMS) does not negatively impact overall surgical outcomes in patients undergoing pancreaticoduodenectomy for treatment of pancreatic or periampullary cancer.
**Test Device**	Devices:<br>WallFlex Biliary RX Fully Covered stent<br>Note: WallFlex Biliary RX Uncovered stents may be used in some cases as outlined in the protocol<br>Cleared Indications:<br>The WallFlex Biliary stents are indicated for use in the palliative treatment of biliary strictures produced by malignant neoplasms and relief of malignant biliary obstruction prior to surgery<br>Note: The WallFlex Biliary FC stent is also indicated for the treatment of benign biliary strictures per CE Mark or other local regulations
**Study Design**	• Prospective, multi-center, randomized, post-market (on label)<br>• Patients will be block randomized at baseline in a 1:1 ratio between **Group 1:** No Pre-Operative Biliary Drainage and **Group 2:** Pre-Operative Biliary Drainage with a SEMS.
**Planned Number of Subjects**	294 total number of subjects:<br>• Group 1: 147<br>• Group 2: 147
**Planned Number of Centers**	Up to 20 sites world-wide
**Primary Endpoint**	Serious pre-operative, operative and post-operative adverse events to 120 days post randomization or to 30 days post-surgery, whichever comes last
**Secondary Endpoints**	1. Adverse events: rate, severity, seriousness, relatedness to stent or endoscopic or surgical procedure, impact on time of surgery,

	length of hospitalization and ICU stay<br>2. Time to surgery<br>3. Curative Intent Surgery details pertaining to intraoperative assessment of resectability, surgical resection and reconstruction techniques<br>4. Intraoperative blood loss and blood transfusions, duration of surgery<br>5. Biliary obstructive symptoms assessment (Week 2, surgery/transition to palliation, 30 day post-operative follow up and 120 day follow up)<br>6. Improvement of LFT levels as relative to baseline (Week 2, surgery/transition to palliation, 30 day post-operative follow up and 120 day follow up)<br>7. Stent placement success: ability to deploy the stent in satisfactory position across the stricture (Group 2)<br>8. Stent removal success: successful SEMS removal, either *en bloc* at time of surgery or endoscopically prior to surgery without stent removal related SAEs<br>9. Number, type, reason and timing of biliary re-interventions<br>10. Number and duration of hospital and ICU admissions
**Follow-up Schedule and Assessments**	• **Screening:** Count all consecutive patients seen at investigational site presenting with biliary obstructive symptoms and suspicion of pancreatic cancer, distal common bile duct cholangiocarcinoma or peri-ampullary cancer. Of those, a subset will be invited to participate in trial: Informed Consent (enrollment) and Eligibility Criteria Assessment<br>• **Baseline Visit (Day 0):** Demographics, Medical History, Tumor Diagnosis, Staging and Characteristics, Assessment of Biliary Obstructive Symptoms, Laboratory Tests, Randomization<br>• **Stent Placement Procedure Visit (Group 2 only, Day 0):** Stent Details, Procedure Details, Adverse Events (AEs)<br>• **Pre-Operative Visit (Week 2):** Assessment of Biliary Obstructive Symptoms, Laboratory Tests, AEs<br>***Group 1 (No Pre-Operative Biliary Drainage):*** Patients will undergo resection per institutional standard of practice. Time to surgery is not to exceed 4 weeks unless there is a need to delay planned surgery due to a complication, biliary obstructive symptoms, decline in patient physiologic status, or other factors that preclude surgery.<br>***Group 2 (Pre-Operative Biliary Drainage with a SEMS):*** Patients will undergo resection after resolution of jaundice is achieved (defined as Bilirubin below 100μmol per liter), or at 4 weeks,

	whichever comes first.<br>• **Biliary Reintervention Visit (as needed):** Timing, Reason for Biliary Reintervention, Type of Biliary Reintervention, AEs<br>Group 1 patients who fail their treatment algorithm by requiring biliary drainage prior to surgery will receive a study SEMS.<br>Group 2 patients who require re-stenting or placement of a stent for a new stricture, will receive a study SEMS.<br>• **Curative Intent Surgery:** Assessment of Biliary Obstructive Symptoms, Laboratory Tests, Operative Details, Stent Removal, Blood Loss, Intra- and Post-Operative Transfusion, Post-Operative Course, Specimen Pathology, AEs<br>• **Transition to Palliative Management Visit (as needed):** Assessment of Biliary Obstructive Symptoms, Laboratory Tests, AEs<br>• **Post-Operative Follow up Visit (30 days Post-Surgery, if applicable):** Assessment of Biliary Obstructive Symptoms, Laboratory Tests, AEs<br>• **Long Term Follow-up Visit (120 days--150 days post randomization):** Assessment of Biliary Obstructive Symptoms, Laboratory Tests, AEs<br>Patients who undergo surgery as planned will be evaluated at 30 days post-surgery and will be followed up to 120 days after randomization.<br>Patients with delay in planned surgery may require follow up longer than 120 days to allow for a 30 day post-surgery follow up visit but follow up is not to exceed 150 days post-randomization.<br>Patients with a delay in planned surgery whose rescheduled surgery is unable to occur within 120 days will be followed up to 120 days after randomization.<br>Patients who do not undergo surgery as planned due to conversion to palliative management or patient choice will be followed up to 120 days after randomization. If patient's course of treatment requires placement of a biliary stent, stenting will be done per standard of care at each institution. If a metal stent is required, a study WallFlex Biliary FC stent will be placed.
**Key Inclusion Criteria**	1. Age 18 or older<br>2. Willing and able to comply with the study procedures and provide written informed consent to participate in the study<br>3. Diagnosis of probable pancreatic cancer, distal common bile duct (CBD) cholangiocarcinoma and other periampullary cancers (histology not required)<br>4. Biliary obstructive symptoms or signs

	5. Bilirubin level at/above 100 μmol per liter (5.8 mg/dL)<br>6. Distal biliary obstruction consistent with pancreatic cancer, distal CBD cholangiocarcinoma or other periampullary malignancy<br>7. Location of distal biliary obstruction is such that it would allow the proximal end of a stent to be positioned at least 2 cm from the hilum<br>8. Patients deemed as resectable by pancreatic protocol CT or MRI<br>9. Surgical candidate per pancreatobiliary surgeon after multi-disciplinary discussion<br>10. Surgery intent within 4 weeks<br>11. Endoscopic and surgical treatment to be provided by same team
**Key Exclusion Criteria**	1. Biliary strictures caused by confirmed benign tumors<br>2. Biliary strictures caused by malignancies other than pancreatic, cancer, distal CBD cholangiocarcinoma and other periampullary cancers<br>3. Surgically altered biliary tract anatomy, not including prior cholecystectomy<br>4. Neoadjuvant chemotherapy for current malignancy<br>5. Palliative indication due to reasons other than surgical candidate status<br>6. Previous biliary drainage by ERCP/PTC<br>7. Patients for whom endoscopic techniques are contraindicated<br>8. Participation in another investigational trial within 90 days<br>9. Pregnancy
**Primary Statistical Hypothesis**	A literature search of preoperative biliary drainage with self-expanding metal stents in patients with pancreatic or periampullary cancer yielded eight articles with 305 patients.[1-8]<br>A meta-analysis of the probability for pre-operative, operative and peri-operative complications was performed. A rate of 24.2% [95% CI: 13.2%, 37.2%] was calculated using the eight articles.<br><br>Statistical testing will be performed to determine if the rate of complications for the *Pre-Operative Biliary Drainage with SEMS* group is non-inferior to the *No Pre-Operative Biliary Drainage* group. The null hypothesis is that the complication rate is inferior in the *Pre-Operative Biliary Drainage with SEMS* versus the *No Pre-Operative Biliary Drainage* group:<br><br>$Ho: \pi_{test} - \pi_{control} \geq \Delta$ (Inferior)

	$Ha: \pi_{test} - \pi_{control} < \Delta$ (Non-inferior)<br><br>where π_{test} and $\pi_{control}$ are the probabilities of having pre-operative, operative and peri-operative complications in the *Pre-Operative Biliary Drainage with SEMS* arm and *No Pre-Operative Biliary Drainage* arm respectively, and Δ is defined as the non-inferiority margin.<br><br>The sample size was calculated for a one-sided 0.050 exact Farrington-Manning test using StatXact 9®. If the P value from the exact Farrington-Manning test is <0.05 then the *Pre-Operative Biliary Drainage with SEMS* group will be considered non-inferior to the *No Pre-Operative Biliary Drainage* group. The expected probability of complications in the *Pre-Operative Biliary Drainage with SEMS* arm and *No Pre-Operative Biliary Drainage* arm is 37.2%, which was taken from the upper limit of the 95% CI from the meta-analysis described above and from the only available Level 1 study comparing no drainage to preoperative stenting in which the complication rate was reported to be 39% in the no drainage arm.[9] The non-inferiority margin (Δ) is 15%. Given these assumptions and a one-sided 5% significance level, 2 x 132 = 264 subjects will provide 80% power to reject the null hypothesis, that the *Pre-Operative Biliary Drainage with SEMS* group is inferior to the *No Pre-Operative Biliary Drainage* group.<br><br>To compensate for possible loss of subjects after enrollment and complete assessment of inclusion/exclusion criteria, an additional 10% of subjects will be enrolled, for a total of 2 x 147 = 294 subjects.

TABLE OF CONTENTS

1. INTRODUCTION 61
1.1. Pancreatic and Periampullary cancer 61
1.2. Treatment of Pancreatic/Periampullary Cancer 61
1.3. Biliary Drainage in Patients with Pancreatic/Periampullary Cancer 62
2. PRIMARY OBJECTIVE 62
3. DESIGN 62
3.1. Scale, Duration and Investigators 62
3.2. Treatment Assignment 63
4. ENDPOINTS 65
4.1. Primary Endpoint 65
4.2. Secondary Endpoints 65
5. SUBJECT SELECTION 65
5.1. Study Population and Eligibility 65
5.2. Inclusion Criteria 65
5.3. Exclusion Criteria 66
6. STUDY DEVICES 66
7. STUDY VISITS 68
7.1. Visit Schedule 68
7.2. Screening 70
7.3. Baseline Visit (Day 0)—Office Visit 70
7.4. Stent Placement Procedure Visit (Group 2 subjects only)—Office Visit (may occur during Baseline visit) 70
7.5. Pre-Operative Visit (Week 2 +/- 3 days)—Telephone or Office Visit 71
7.6. Biliary Reintervention Visit (Group 1 or 2) —as needed 71
7.7. Curative Intent Surgery—Office Visit (within 4 weeks, or delayed surgery by 120 days post-randomization) 71
7.8. Transition to Palliative Management Visit (if applicable)—Telephone or Office Visit 72
7.9. Post-Operative Follow-up Visit (30 days Post-Surgery +/- 7 days, if applicable)—Telephone or Office Visit 72

7.10. Long Term Follow-up Visit (120 days—150 days post randomization +/- 14 days)—Telephone or Office Visit....73

7.11. Study Completion....73

8. STATISTICAL CONSIDERATIONS....73

8.1. Hypothesis....73

8.2. Sample Size....74

8.3. Analysis Populations....74

8.3.1. Enrolled Cohort....74

8.3.2. Intent-to-Treat Cohort....74

8.3.3. Per-Protocol Cohort....74

8.4. Data Analyses....75

8.4.1. Baseline Data....75

8.4.2. Post-Procedure Endpoints....75

8.4.3. Subgroup Analyses....75

8.4.4. Justification of Pooling....75

8.4.5. Multivariable Analyses....75

8.4.6. Changes to Planned Analyses....76

9. POTENTIAL RISKS AND BENEFITS....76

9.1. Anticipated Adverse Device Effects with Use of WallFlex Biliary Stent....76

9.2. Anticipated Adverse Events in Patients Without Pre-Operative Biliary Drainage....77

9.3. Anticipated Surgical Adverse Events....77

9.4. Risk Minimization Actions....77

9.5. Anticipated Benefits....78

9.6. Risk to Benefit Rationale....78

10. SAFETY REPORTING....78

10.1. Definitions and Classification....78

10.2. Relationship to Study Device(s)....80

10.3. Investigator Reporting Requirements....80

10.3.1. Serious Adverse Events....80

10.3.2. Adverse Events....80

10.3.3. Device Failures, Malfunctions, and Product Nonconformities....81

10.4. Boston Scientific Device Deficiencies....81

10.5. Reporting to Regulatory Authorities / IRBs / ECs / Investigators....81

11. BIBLIOGRAPHY **81**

12. APPENDICES: SPONSOR REQUIRED PROTOCOL SECTIONS **83**

12.1. Data Management **83**

12.1.1. Data Collection, Processing, and Review 83

12.1.2. Data Retention 84

12.2. Amendments **84**

12.3. Deviations **84**

12.4. Device/Equipment Accountability **84**

12.5. Compliance **85**

12.5.1. Statement of Compliance 85

12.5.2. Investigator Responsibilities 85

12.5.3. Delegation of Responsibility 87

12.6. Institutional Review Board/ Ethics Committee **87**

12.7. Sponsor Responsibilities **87**

12.8. Insurance **88**

12.9. Monitoring **88**

12.10. Informed Consent **88**

12.11. Committees **89**

12.11.1. Safety Monitoring Process 89

12.12. Suspension or Termination **90**

12.12.1. Premature Termination of the Study 90

12.12.2. Criteria for Premature Termination of the Study 90

12.12.3. Termination of Study Participation by the Investigator or Withdrawal of IRB/ EC Approval 90

12.12.4. Requirements for Documentation and Subject Follow-up 90

12.12.5. Criteria for Suspending/Terminating a Study Center 91

12.13. Publication Policy **91**

12.14. Definitions of complication criteria (per van der Gaag article): **91**

1. Introduction

1.1. *Pancreatic and Periampullary cancer*

Pancreatic cancer is the fourth leading cause of cancer death in the US and worldwide with 217,000 new cases/year and 213,000 deaths worldwide.[10, 11] Relative 1-year and 5-year survival rates are 26% and 6% respectively.[11]

Pancreatic tumors are usually asymptomatic until the disease is advanced. Presenting symptoms may include weight loss, jaundice, pain, nausea, dyspepsia and depression.[12] Diagnosis and staging is usually achieved with a pancreas protocol CT, a triphasic cross-sectional imaging with thin slices using multidetector CT[13-16]which allows for an assessment of the vascular invasion of the tumor, a major factor in determining resectability. In patients where CT is not possible or contraindicated, MRI with contrast can be used to diagnose and stage pancreatic cancer.[17] Pancreas protocol MRI has been emerging as an equivalent alternative to CT.[18] Endoscopic ultrasound (EUS) can be used complementary to CT to provide additional vascular invasion information or to evaluate periampullary masses.[19, 20] Although histologic diagnosis is not necessary before surgical treatment, a biopsy can be taken using fine-needle aspiration with either EUS-guidance (preferred) or CT-guidance.[18] Diagnostic staging laparoscopy is also utilized, and is mostly valuable when there is a need to rule out sub-radiologic metastases (especially for body and tail lesions).[21, 22]

Based on results from imaging studies described above, patients with pancreatic cancer can be divided into four prognostic subgroups (Stages I-IV) to allow for choice of proper treatment course. These groups are based on local tumor resectability status and presence or absence of distant disease as defined by the tumor-node-metastasis (TNM) staging system of the American Joint Commission on Cancer (AJCC).[23] Patients in whom the primary tumor can be removed are classified as localized resectable (Stages I and II), and patients whose tumors are unresectable are classified as locally advanced (Stage III) and metastatic (Stage IV).[24]

Criteria for resectability vary but usually include the absence of distant metastases and no tumor involvement of major arteries; in cases of venous invasion of the tumor, there must be a suitable segment of portal vein (above) and superior mesenteric vein (below) the site of venous involvement to allow for venous reconstruction.[25]

1.2. *Treatment of Pancreatic/Periampullary Cancer*

The only potential curative treatment option for patients with resectable pancreatic/periampullary cancers is curative-intent-surgery (CIS) by pancreaticoduodenectomy (Whipple procedure)[26] with a long-term survival rate of 20% and a median survival of 12-20 months.[24] Only 15-20% of patients presenting with pancreatic cancer have potentially resectable tumors.[27]

Most pancreatic cancers arise in the head of the pancreas and often cause bile duct obstruction resulting in jaundice, while body and tail lesions are insidious in their development and usually far advanced when detected. Therefore most patients who are candidates for CIS have tumors in the head of the pancreas.[28] The most common type of

pancreatic cancer is adenocarcinoma, originating from the pancreatic duct cells[24] and accounting for over 90% of pancreatic malignancies.[12]

1.3. *Biliary Drainage in Patients with Pancreatic/Periampullary Cancer*

Biliary obstruction associated with pancreatic/periampullary cancer occurs in up to 70% of patients.[29] Hyperbilirubinemia resulting from biliary obstruction adversely affects liver, cardiovascular and renal function,[26] and the subsequent cholestasis which is thought to impair immune response and clotting[1] may negatively impact the outcome of CIS. Preoperative biliary drainage potentially reverses these factors and could result in improved outcome of pancreaticoduodenectomy.[26]

Preoperative biliary decompression has traditionally been achieved with plastic stents, however, these stents have been associated with high complication rates and relatively low success rates in preoperative management of biliary obstruction.[1-3, 9, 30]

In recent publications, SEMS have been shown to be superior to plastic stents for preoperative biliary drainage in pancreatic cancer[1] because of longer patency achieved by a greater stent diameter,[31] fewer ERCP procedures, and fewer episodes of cholangitis/cholestasis due to stent occlusion while awaiting surgery (15% using SEMS vs 93% using plastic stents).[2]

2. Primary Objective

The primary objective is to demonstrate that preoperative biliary drainage using SEMS does not negatively impact overall surgical outcomes in patients undergoing pancreaticoduodenectomy for treatment of pancreatic or periampullary cancer.

3. Design

3.1. *Scale, Duration and Investigators*

This is a post-market (on label), prospective, multi-center, randomized, non-blinded study.

Patients will be followed for 120 days after randomization. Only patients who experience a delay in their planned surgery due to factors such as but not limited to complications, biliary obstructive symptoms, patient physiologic status, etc. may be followed up to 150 days to allow for a 30 days post-surgery follow up visit.

There will be up to 20 participating centers world-wide with anticipated enrollment of 294 patients, 147 per group.

At each investigational center, there will be one principal investigator (PI) and at least one Co-PI. At a minimum, there will be one gastroenterologic endoscopist and one pancreaticobiliary surgeon among the investigators. Although more than two investigators per center are allowed, it is encouraged that the number be kept small.

3.2. *Treatment Assignment*

Patients will be randomized at baseline in equal proportions of 1:1 ratio between Group 1 and Group 2 as follows:

- **Group 1**: No pre-operative biliary drainage (patients will not receive a stent)
- **Group 2**: Pre-operative biliary drainage with a SEMS

Block randomization through an online database system will be used. Randomization will be stratified by study center.

Figure 1 below provides an overview of various patient management scenarios in the form of a study flow chart.

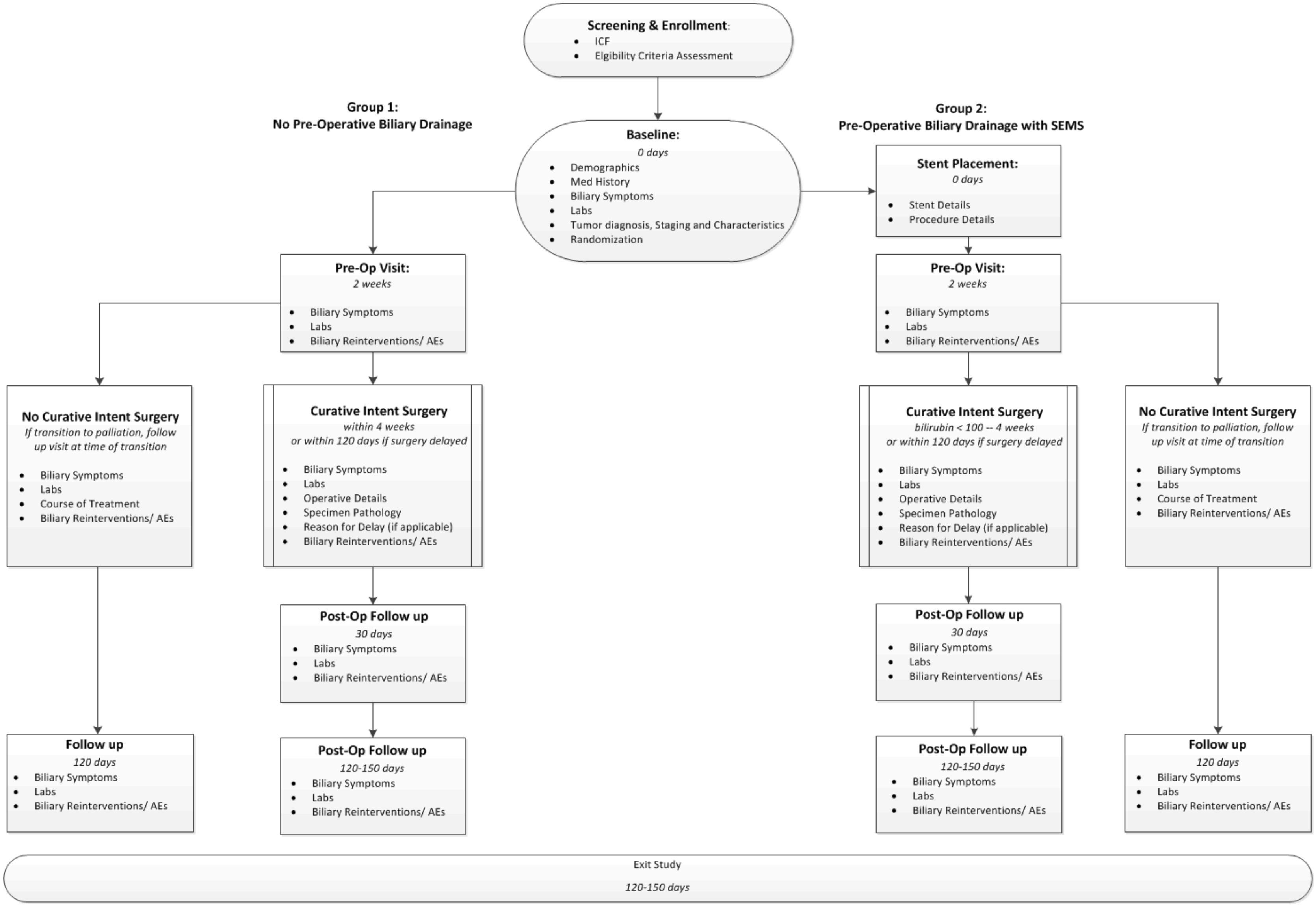
Screening & Enrollment:
ICF
Elgibility Criteria Assessment
Group 1:
No Pre-Operative Biliary Drainage
Group 2:
Pre-Operative Biliary Drainage with SEMS
Baseline:
0 days
Demographics
Med History
Biliary Symptoms
Labs
Tumor diagnosis, Staging and Characteristics
Randomization
Stent Placement:
0 days
Stent Details
Procedure Details
Pre-Op Visit:
2 weeks
Biliary Symptoms
Labs
Biliary Reinterventions/ AEs
Pre-Op Visit:
2 weeks
Biliary Symptoms
Labs
Biliary Reinterventions/ AEs
No Curative Intent Surgery
If transition to palliation, follow up visit at time of transition
Biliary Symptoms
Labs
Course of Treatment
Biliary Reinterventions/ AEs
Curative Intent Surgery
within 4 weeks
or within 120 days if surgery delayed
Biliary Symptoms
Labs
Operative Details
Specimen Pathology
Reason for Delay (if applicable)
Biliary Reinterventions/ AEs
Curative Intent Surgery
bilirubin < 100 -- 4 weeks
or within 120 days if surgery delayed
Biliary Symptoms
Labs
Operative Details
Specimen Pathology
Reason for Delay (if applicable)
Biliary Reinterventions/ AEs
No Curative Intent Surgery
If transition to palliation, follow up visit at time of transition
Biliary Symptoms
Labs
Course of Treatment
Biliary Reinterventions/ AEs
Post-Op Follow up
30 days
Biliary Symptoms
Labs
Biliary Reinterventions/ AEs
Post-Op Follow up
30 days
Biliary Symptoms
Labs
Biliary Reinterventions/ AEs
Follow up
120 days
Biliary Symptoms
Labs
Biliary Reinterventions/ AEs
Post-Op Follow up
120-150 days
Biliary Symptoms
Labs
Biliary Reinterventions/ AEs
Post-Op Follow up
120-150 days
Biliary Symptoms
Labs
Biliary Reinterventions/ AEs
Follow up
120 days
Biliary Symptoms
Labs
Biliary Reinterventions/ AEs
Exit Study
120-150 days

4. Endpoints

4.1. *Primary Endpoint*

Serious pre-operative, operative and post-operative adverse events to 120 days post randomization or to 30 days post-surgery, whichever comes last

4.2. *Secondary Endpoints*

1. Adverse events: rate, severity, seriousness, relatedness to stent or endoscopic or surgical procedure, impact on time of surgery, length of hospitalization and ICU stay
2. Time to surgery
3. Curative Intent Surgery details pertaining to intraoperative assessment of resectability, surgical resection and reconstruction techniques
4. Intraoperative blood loss and blood transfusions, duration of surgery
5. Biliary obstructive symptoms assessment (Week 2, surgery/transition to palliation, 30 day post-operative follow up and 120 day follow up)
6. Improvement of LFT levels as relative to baseline (Week 2, surgery/transition to palliation, 30 day post-operative follow up and 120 day follow up)
7. Stent placement success: ability to deploy the stent in satisfactory position across the stricture (Group 2)
8. Stent removal success: successful SEMS removal, either *en bloc* at time of surgery or endoscopically prior to surgery without stent removal related SAEs
9. Number, type, reason and timing of biliary re-interventions
10. Number and duration of hospital and ICU admissions

5. Subject Selection

5.1. *Study Population and Eligibility*

All consecutive patients seen at an investigational site presenting with biliary obstructive symptoms with suspicion of pancreatic cancer, periampullary cancer or distal CBD cholangiocarcinoma will be counted. Of those, a subset will be invited to participate in trial and is considered enrolled after signing the study specific Informed Consent Form (ICF). A screening log will be maintained to document the assessment of eligibility criteria provided in Section 5.2 and Section 5.3.

5.2. *Inclusion Criteria*

Subjects who meet all of the criteria listed below may be given consideration for inclusion in this clinical investigation, provided no exclusion criterion (see Section 5.3) is met.

1. Age 18 or older
2. Willing and able to comply with the study procedures and provide written informed consent to participate in the study
3. Diagnosis of probable pancreatic cancer, distal common bile duct (CBD) cholangiocarcinoma and other periampullary cancers (histology not required)
4. Biliary obstructive symptoms or signs
5. Bilirubin level at/above 100μmol per liter (5.8 mg/dL)
6. Distal biliary obstruction consistent with pancreatic cancer, distal CBD cholangiocarcinoma or other periampullary malignancy
7. Location of distal biliary obstruction is such that it would allow the proximal end of a stent to be positioned at least 2cm from the hilum
8. Patients deemed as resectable by pancreatic protocol CT or MRI
9. Surgical candidate per pancreatobiliary surgeon after multi-disciplinary discussion
10. Surgery intent within 4 weeks
11. Endoscopic and surgical treatment to be provided by same team

5.3. *Exclusion Criteria*

Subjects who meet any one of the following criteria will be excluded from this clinical study.

1. Biliary strictures caused by confirmed benign tumors
2. Biliary strictures caused by malignancies other than pancreatic cancer, distal CBD cholangiocarcinoma and other periampullary cancers
3. Surgically altered biliary tract anatomy, not including prior cholecystectomy
4. Neoadjuvant chemotherapy for current malignancy
5. Palliative indication due to reasons other than surgical candidate status
6. Previous biliary drainage by ERCP/PTC
7. Patients for whom endoscopic techniques are contraindicated
8. Participation in another investigational trial within 90 days
9. Pregnancy

6. Study Devices

The WallFlex Biliary RX Fully Covered stent will be the default choice for treatment of patients in Group 2. A WallFlex Biliary RX Uncovered stent may be used at the joint discretion of the endoscopic and surgical investigators for documented reasons related to the ductal anatomy, namely stricture involving the cystic duct confluence, low cystic duct confluence, or other anatomic considerations to be specified.

The WallFlex Biliary RX Fully Covered and the WallFlex Biliary RX Uncovered Stent Systems are indicated for use in the palliative treatment of biliary strictures produced by malignant neoplasms and relief of malignant biliary obstruction prior to surgery; the WallFlex Biliary RX Fully Covered Stent System is also indicated for treatment of benign biliary strictures, per CE Mark or other local regulations. For a detailed description of the WallFlex Biliary Stent Systems, please reference the Directions for Use (DFU) included in each device package.

Investigators should use the WallFlex Biliary RX Fully Covered and Uncovered Stent Systems in accordance with the DFUs.

Study devices are labeled on the box and inner pouch and contain information including but not limited to: device name and dimensions, lot number, expiration date, name of legal manufacturer, and investigational use statement. Device labeling will be provided in local language(s) as per national regulations.

Study devices will be available in the following dimensions:

WallFlex Biliary RX Stent	Diameter	Length	Delivery System Diameter	Guidewire Diameter
**Uncovered**	8 mm<br><br>10 mm	40, 60, 80<br><br>40, 60, 80	8 Fr	.035”
**Fully Covered**	8 mm<br><br>10 mm	60, 80mm<br><br>40, 60, 80mm	8.5 Fr	.035”

Stent placement should be such that the proximal end of the stent is minimum 2 cm from the hilum. Performing a biliary or pancreatic sphincterotomy or enlarging a prior sphincterotomy will be done at the discretion of the endoscopist.

In case of a failed stent placement due to a device event, a new attempt to place a stent will be made. If a stent placement is not possible due to non-device related reasons (such as inability to cannulate the CBD or reach the papilla, extensive tumor growth at site of papilla, etc.), interventional radiologic (IR) access is allowed and, where possible, should be associated with placement of a study stent over a transhepatically inserted guide-wire in a "rendez-vous" procedure. Percutaneous transhepatic cholangiograpy (PTC) and a subsequent internal drainage with a stent placement may be done at the same time (one-stage procedure), or PTC with external drainage may be performed 2 -3 days before stent insertion (two-stage procedure) per standard of practice. If access to the biliary tree or endoscopic placement of the study stent through a “rendez-vous” procedure fails and patient requires percutaneous transhepatic biliary drainage (PTBD), then the patient will exit the study.

7. Study Visits

7.1. *Visit Schedule*

The schedule of observations and assessments to take place during the study is outlined in **Table 1** below.

Table 2. Study Event Schedule

Study Visit	Screening	Baseline/ Stent Placement Procedure	Pre-Operative Visit Week 2†	Biliary Reintervention (if applicable)	Curative Intent Surgery	Transition to Palliative Treatment Visit (if applicable)	30 Days Post-Op Follow up Visit (if applicable)	Long Term Follow up Visit 120 Days*
Window	N/A	N/A	±3 d	N/A	N/A	N/A	±7 d	±14 d
ICF	X							
Eligibility Criteria Assessment	X							
Demographics		X						
Medical History		X						
Assessment of Biliary Obstructive Symptoms		X	X		X	X	X	X
Laboratory Tests		X	X		X	X	X	X
Tumor Diagnosis, Staging & Characteristics		X						
Randomization		X						
Stent Details		X		X (if applicable)				
Procedure Details		X		X (if applicable)				
Operative Details					X			
Specimen Pathology					X			
Stent Removal				X (if applicable)	X (if applicable)			
Adverse Events	X (as applicable)							
Protocol Deviations	X (as applicable)							

* Visit may occur up to 150 days post-randomization; † Visit may be conducted in the office or via telephone with liver function tests drawn and sent from local clinic/hospital.

7.2. *Screening*

- Informed Consent
- Eligibility Criteria Assessment

No study-specific testing will be conducted until the subject has signed the study specific informed consent form (ICF).

Informed consent must be obtained for all subjects who are potential study candidates. Subjects will be asked to sign the ICF before any study-specific tests or procedures are performed. The ICF is study-specific and must be approved by the Institutional Review Board (IRB)/Ethics Committee (EC) at each center. Study personnel should explain that even if a subject agrees to participate in the study and signs the study specific ICF, the screening process may demonstrate that the subject is not a suitable candidate for the study.

7.3. *Baseline Visit (Day 0)—Office Visit*

- Demographics
- Medical History
- Tumor Diagnosis, Staging and Characteristics
    - Diagnostic modality
    - Tumor stage
    - Location and size of mass
- Assessment of Biliary Obstructive Symptoms
    - Right Upper Quadrant Pain
    - Fever/Chills
    - Jaundice
    - Itching
    - Dark urine
    - Pale stools
    - Nausea/Vomiting
- Laboratory Tests
    - Serum albumin level
    - Alkaline phosphatase
    - Total Bilirubin
    - Gamma GT
    - SGOT (AST—optional) and SGPT (ALT)
    - Normal Ranges

- Randomization into Group 1 or Group 2

7.4. *Stent Placement Procedure Visit (Group 2 subjects only)—Office Visit (may occur during Baseline visit)*

- Stent Details (including stent choice)
- Procedure Details (including sphincterotomy information)
- Adverse Events (AEs)

7.5. *Pre-Operative Visit (Week 2 +/- 3 days)—Telephone or Office Visit*

- Assessment of Biliary Obstructive Symptoms
  - Right Upper Quadrant Pain
  - Fever/Chills
  - Jaundice
  - Itching
  - Dark urine
  - Pale stools
  - Nausea/Vomiting
- Laboratory Tests
  - Serum albumin level
  - Alkaline phosphatase
  - Total Bilirubin
  - Gamma GT
  - SGOT (AST—optional) and SGPT (ALT)
- AEs

7.6. *Biliary Reintervention Visit (Group 1 or 2) —as needed*

- Timing
- Reason for Biliary Reintervention
- Type of Biliary Reintervention (including SEMS placement or removal)
- Adverse Events (AEs)

7.7. *Curative Intent Surgery—Office Visit (within 4 weeks, or delayed surgery by 120 days post-randomization)*

- Assessment of Biliary Obstructive Symptoms
  - Right Upper Quadrant Pain
  - Fever/Chills
  - Jaundice
  - Itching
  - Dark urine
  - Pale stools
  - Nausea/Vomiting
- Laboratory Tests
  - Serum albumin level
  - Alkaline phosphatase
  - Total Bilirubin
  - Gamma GT
  - SGOT (AST—optional) and SGPT (ALT)
- Operative details:
  - Exploration
  - Resection (including SEMS removal)
  - Reconstruction

- Blood Loss
- Intra and Post-Operative Transfusion
- Post-Operative Course
- Specimen Pathology (including margin status) per World Health Organization (WHO) classification system
- AEs

7.8. *Transition to Palliative Management Visit (if applicable)—Telephone or Office Visit*

- Assessment of Biliary Obstructive Symptoms
  - Right Upper Quadrant Pain
  - Fever/Chills
  - Jaundice
  - Itching
  - Dark urine
  - Pale stools
  - Nausea/Vomiting
- Laboratory Tests
  - Serum albumin level
  - Alkaline phosphatase
  - Total Bilirubin
  - Gamma GT
  - SGOT (AST—optional) and SGPT (ALT)
- AEs

7.9. *Post-Operative Follow-up Visit (30 days Post-Surgery +/- 7 days, if applicable)—Telephone or Office Visit*

- Assessment of Biliary Obstructive Symptoms
  - Right Upper Quadrant Pain
  - Fever/Chills
  - Jaundice
  - Itching
  - Dark urine
  - Pale stools
  - Nausea/Vomiting
- Laboratory Tests
  - Serum albumin level
  - Alkaline phosphatase
  - Total Bilirubin
  - Gamma GT
  - SGOT (AST—optional) and SGPT (ALT)
- AEs

7.10. *Long Term Follow-up Visit (120 days—150 days post randomization +/- 14 days)—Telephone or Office Visit*

- Assessment of Biliary Obstructive Symptoms
  - Right Upper Quadrant Pain
  - Fever/Chills
  - Jaundice
  - Itching
  - Dark urine
  - Pale stools
  - Nausea/Vomiting
- Laboratory Tests
  - Serum albumin level
  - Alkaline phosphatase
  - Total Bilirubin
  - Gamma GT
  - SGOT (AST—optional) and SGPT (ALT)
- AEs

7.11. *Study Completion*

Each subject will be followed for 120 days from the time of randomization. In cases where patients experience a delay of planned surgery, the long term follow up can be extended up to 150 days post randomization to allow for completion of a Follow-up Visit at 30 days after surgery.

A subject will be considered lost to follow-up if the subject remains unresponsive to communication after three documented attempts by study staff.

8. Statistical Considerations

8.1. *Hypothesis*

Statistical testing will be performed to determine if the rate of complications for the Pre-Operative Biliary Drainage with SEMS group is non-inferior to the No Pre-Operative Biliary Drainage group. The null hypothesis is that the complication rate is inferior in the Pre-Operative Biliary Drainage with SEMS versus the No Pre-Operative Biliary Drainage group:

Ho: $\pi_{test} - \pi_{control} \geq \Delta$ (Inferior)

Ha: $\pi_{test} - \pi_{control} < \Delta$ (Non-inferior)

where π_{test} and $\pi_{control}$ are the probabilities of having pre-operative, operative and peri-operative complications in the Pre-Operative Biliary Drainage with SEMS arm and No Pre-Operative Biliary Drainage arm respectively, and Δ is defined as the non-inferiority margin.

8.2. *Sample Size*

A literature search of preoperative biliary drainage with self-expanding metal stents in patients with pancreatic or periampullary cancer yielded 8 articles with 305 patients.[21-24, 27-31]

The following meta-analysis of the probability for pre-operative, operative and post-operative complications was done:

- A rate of 24.2% [95% CI: 13.2%, 37.2%] was calculated using the 8 articles.

The sample size was calculated for a one-sided 0.050 exact Farrington-Manning test using StatXact 9®. If the P value from the exact Farrington-Manning test is <0.05 then the *Pre-Operative Biliary Drainage with SEMS* group will be considered non-inferior to the *No Pre-Operative Biliary Drainage* group. The expected probability of complications in the *Pre-Operative Biliary Drainage with SEMS* arm and *No Pre-Operative Biliary Drainage* arm is 37.2%, which was taken from the upper limit of the 95% CI from the meta-analysis described above and from the only available Level 1 study comparing no drainage to preoperative stenting in which the complication rate was reported to be 39% in the no drainage arm.[9] The non-inferiority margin (Δ) is 15%. Given these assumptions and a one-sided 5% significance level, 2 x 132 = 264 subjects will provide 80% power to reject the null hypothesis, that the *Pre-Operative Biliary Drainage with SEMS* group is inferior to the *No Pre-Operative Biliary Drainage* group.

To compensate for possible loss of subjects after enrollment and complete assessment of inclusion/exclusion criteria, an additional 10% of subjects will be enrolled, for a total of 2 x 147 = 294 subjects.

8.3. *Analysis Populations*

8.3.1. Enrolled Cohort

A subject is considered “enrolled” after signing the study-specific ICF. Subjects who sign the ICF but subsequently do not meet one or more of the eligibility criteria provided in Section 5.2 and Section 5.3 will be considered screen failures and excluded from the study.

8.3.2. Intent-to-Treat Cohort

This cohort consists of those “enrolled” subjects who meet all inclusion/exclusion criteria and are subsequently randomized.

8.3.3. Per-Protocol Cohort

The per-protocol cohort is a subset of the ITT subjects who are treated per protocol after randomization (Group 1 no stent, and Group 2 received a study stent) and no major protocol deviations (ICH E9 definitions).

8.4. *Data Analyses*

All statistical analyses will be done using The SAS System software, version 8 or higher (Copyright © 2000 SAS Institute Inc., SAS Campus Drive, Cary, North Carolina 27513, USA. All rights reserved).
The distribution of prognostic factors between patients with and without data will be examined. Statistical models that account for censored data will be employed in appropriate circumstances, e.g. for time-to-event outcomes. Sensitivity analyses will be conducted to assess the impact of missing data on the interpretation of the results, e.g. a tipping point analysis.

8.4.1. Baseline Data

Baseline data will be summarized. Subject demographics, clinical history, risk factors, LFTs, tumor diagnosis, and obstruction symptoms will be summarized using descriptive statistics (e.g., mean, standard deviation, n, minimum, maximum) for continuous variables and frequency statistics for discrete variables.

8.4.2. Post-Procedure Endpoints

Post-procedure information will be collected at regularly scheduled follow-up examinations as detailed in the clinical study event schedule and will be summarized using descriptive statistics for continuous variables (e.g., mean, standard deviation, n, minimum, maximum) and frequency tables or proportions for discrete variables.

8.4.3. Subgroup Analyses

The subgroup analyses will include tabulating the primary endpoint and select secondary endpoints by gender.

8.4.4. Justification of Pooling

The analyses will be presented using pooled data across institutions. An analysis of the poolability will be made using logistic regression for binary outcomes, analysis of covariance for continuous outcomes, or proportional hazards regression for time-to-event outcomes, to assess differences between study institutions and to justify pooling data across institutions.

8.4.5. Multivariable Analyses

Univariate and multivariate analyses will be performed to assess possible predictors of the primary endpoint. Possible predictors may include any but not limited to demographic/baseline data and medical history data. Factors from the univariate model with $p \leq 0.20$ will also be modeled multivariately using a stepwise procedure in a generalized linear model or Cox Proportional Hazards model. The significance thresholds for entry and exit into the model will be set to $p<0.10$.

8.4.6. Changes to Planned Analyses

Any changes to the planned statistical analyses made prior to performing the analyses will be documented in a Statistical Analysis Plan approved prior to performing the analyses.

9. Potential Risks and Benefits

9.1. *Anticipated Adverse Device Effects with Use of WallFlex Biliary Stent*

As per the commercial DFU included with the study devices, the potential complications associated with the use of the WallFlex Biliary FC and UC Stent may include, but are not limited to:

- Pain
- Bleeding
- Fever
- Nausea
- Vomiting
- Infection
- Inflammation
- Recurrent obstructive jaundice
- Stent occlusion
- Tumor overgrowth around ends of stent
- Tumor ingrowth through the stent
- Mucosal hyperplasia
- Cholangitis
- Cholecystitis*
- Pancreatitis
- Bile duct or duodenum ulceration
- Perforation of duodenum or bile duct
- Stent migration
- Death (other than that due to normal disease progression)
- Stent misplacement
- Perforation of the gallbladder due to the stent covering the cystic duct*
- Stent fracture
- Hepatic abscess

  *Note: In a small clinical trial of this device, two out of four (50%) subjects who had a stent placed across the cystic duct developed cholecystitis. One of these subjects suffered a perforated gallbladder due to the stent covering the cystic duct, requiring a drain to be placed.

As per the commercial DFU included with the study devices, potential complications associated with stent removal during an ERCP procedure include, but are not limited to:

- Pain

- Bleeding
- Fever
- Nausea
- Vomiting
- Infection
- Inflammation
- Recurrent obstructive jaundice
- Mucosal hyperplasia
- Cholangitis
- Cholecystitis
- Pancreatitis
- Ulceration of duodenum or bile duct
- Perforation of duodenum or bile duct
- Death (other than that due to normal disease progression)
- Impaction to the common bile duct wall

9.2. *Anticipated Adverse Events in Patients Without Pre-Operative Biliary Drainage*

- Severe jaundice
- Cholangitis

9.3. *Anticipated Surgical Adverse Events*

- Pancreaticojejunostomy leakage
- Delayed gastric emptying
- Biliary leakage
- Gastro/-duodenojejunostomy leakage
- Intra-abdominal abscess formation
- Wound infection
- Portal Vein Thrombosis
- Cholangitis
- Hemorrhage
- (Emergency) (re)laparotomy
- Pneumonia
- Myocardial infarction
- Mortality

9.4. *Risk Minimization Actions*

Additional risks may exist. Risks can be minimized through compliance with this protocol, performing procedures in the appropriate hospital environment, adherence to subject selection criteria, close monitoring of the subject's physiologic status during research procedures and/or follow-ups and by promptly supplying BSC with all pertinent information required by this protocol. The latter may facilitate inter-center communications regarding serious AEs.

9.5. *Anticipated Benefits*

Subjects may not receive any benefit from participating in this study but this study may provide a future benefit to medical science and other patients. To date there is no broadly accepted standard of practice pertaining to pre-operative drainage of patients as defined by the inclusion and exclusion criteria of the study.

9.6. *Risk to Benefit Rationale*

Based on prior BSC's clinical studies and collected reports in literature to-date, the risk-to-benefit ratio is within reason for forseeable risks. However, literature reports do not always capture all side effects. Observation and follow up of subjects is required as outlined in the protocol.

10. Safety Reporting

10.1. *Definitions and Classification*

Adverse event definitions are provided in Table 10.1-1.

Table 10.1-1: Adverse Event Definitions

Term	Definition
Adverse Event (AE)<br><br>*Ref: ISO 14155-2011*	Any untoward medical occurrence, unintended disease or injury, or untoward clinical signs (including abnormal lab findings) in subjects, users or other persons, whether or not related to the investigational medical device. This includes events related to:<br>• The investigational medical device or comparator<br>• The procedures involved (study-required)<br>For users/other persons, this definition is restricted to events related to the investigational device
Adverse Device Effect (ADE)<br><br>*Ref: ISO 14155-2011*	Adverse event related to the use of an investigational medical device:<br>• This includes adverse events resulting from insufficient or inadequate instructions for the use, deployment, implantation, installation or operation, or any malfunction of the investigational medical device.<br>• This includes any event resulting from use error or from intentional misuse of the investigational medical device.
Serious Adverse Event (SAE)<br><br>*Ref: ISO 14155-2011*	An adverse event that:<br>• Led to death<br>• Led to a serious deterioration in the health of the subject that either resulted in:

Table 10.1-1: Adverse Event Definitions

Term	Definition
	○ a life-threatening illness or injury, or<br>○ a permanent impairment of a body structure or a body function, or<br>○ in-patient hospitalization or prolonged hospitalization (of an existing hospitalization), or<br>○ medical or surgical intervention to prevent life-threatening illness or injury or permanent impairment to a body structure or a body function<br>• Led to fetal distress, fetal death, or a congenital abnormality or birth defect.<br>**Note**: Planned hospitalization for a pre-existing condition, or a procedure required by the protocol, without serious deterioration in health, is not considered a serious adverse event.<br>**Note**: For SAE reporting requirements see the information below for SADE.
Serious Adverse Device Effect (SADE)<br><br>*Ref: ISO 14155-2011*	An adverse device effect that has resulted in any of the consequences characteristic of a serious adverse event.<br>**Note**: All SAEs that could have led to a SADE if suitable action had not been taken or if circumstances had been less fortunate shall be reported as required by the local IRB/EC, national regulations, or the protocol. If applicable, see MEDDEV 2.7/3 12/2010 for reporting timeline requirements.
Device Deficiency<br><br>*Ref: ISO 14155-2011*<br><br>*Ref: MEDDEV 2.7/3 12/2010*	A device deficiency is any inadequacy of a medical device with respect to its identity, quality, durability, reliability, safety or performance.<br>**Note**: Device deficiencies include malfunctions, use errors, and inadequate labeling.<br>**Note**: All device deficiencies that could have led to a SADE if suitable action had not been taken or if circumstances had been less fortunate shall be reported as required by the local IRB/EC, national regulations, or the protocol. If applicable, see MEDDEV 2.7/3 12/2010 for reporting timeline requirements.

Abbreviations: EC=Ethics Committee; IRB=Institutional Review Board

Underlying diseases are not reported as AEs unless there is an increase in severity or frequency during the course of the investigation. Death should not be recorded as an AE, but should only be reflected as an outcome of a specific SAE (see Table 10.1-1 for AE definitions).

Any AE experienced by the study subject after informed consent, whether during or subsequent to the procedure, must be recorded in the eCRF.

Refer to Section 9 for the known risks associated with the study device(s).

Adverse events that occur during the course of the study will be evaluated against a list of pre-specified complication criteria as defined in Section 12.14. Determination of whether an AE meets the definition of a pre-specified complication will be made by study physicians.

10.2. *Relationship to Study Device(s)*

The Investigator must assess the relationship of the AE to the study device as related or unrelated. See criteria in Table 10.2-2:

Table 10.2-2: Criteria for Assessing Relationship of Study Device to Adverse Event

Classification	Description
**Unrelated**	The adverse event is determined to be due to a concurrent illness or effect of another device/drug and is not related to the investigational product.
**Related**	• The adverse event is determined to be potentially related to the investigational product, and an alternative etiology is equally or less likely compared to the potential relationship to investigational product.<br>• There is a strong relationship to investigational product, or recurs on re-challenge, and another etiology is unlikely.<br>• There is no other reasonable medical explanation for the event.

10.3. *Investigator Reporting Requirements*

Investigators will be required to report all SAEs and ADEs.

10.3.1. Serious Adverse Events

These events should be reported to the Global Safety Office and/or Project Manager **within 2 business days** of first becoming aware of the event. Events should be documented in the eCRF and all relevant source documentation for the event should be provided to the Global Safety Office, as applicable.

10.3.2. Adverse Events

Device-related events should be reported to the Global Safety Office and/or Project Manager **within 10 business days** of first becoming aware of the event. Unrelated AEs will not be collected.

10.3.3. Device Failures, Malfunctions, and Product Nonconformities

These events should be reported to Project Manager and/or Global Safety Office **within 1 business day** of first becoming aware of the event. Events should be documented in the eCRF.

10.4. *Boston Scientific Device Deficiencies*

All device deficiencies (including but not limited to failures, malfunctions, use errors, product nonconformities, and labeling errors) will be documented and reported to BSC. If possible, the device(s) should be returned to BSC for analysis. Instructions for returning the investigational device(s) will be provided. If it is not possible to return the device, the investigator should document why the device was not returned and the final disposition of the device.

Device deficiencies, failures, malfunctions, and product nonconformities are not to be reported as adverse events. However, if there is an adverse event that results from a device failure or malfunction, that specific event would be recorded on the appropriate eCRF.

10.5. *Reporting to Regulatory Authorities / IRBs / ECs / Investigators*

BSC is responsible for reporting adverse event information to all participating investigators and regulatory authorities, as applicable.

The Principal Investigator is responsible for informing the IRB/EC and regulatory authorities of SAEs as required by local procedure.

11. Bibliography

32. Decker C, Christein JD, Phadnis MA, Wilcox CM, Varadarajulu S. Biliary metal stents are superior to plastic stents for preoperative biliary decompression in pancreatic cancer. Surg Endosc. 2011 Jul;25(7):2364-7. Epub 2011 Mar 4.
33. Wasan SM, Ross WA, Staerkel GA, Lee GH. Use of Expandable Metallic Biliary Stents in Resectable Pancreatic Cancer. Am J Gastroenterol. 2005;100:2056–2061.
34. Mullen JT, Lee JH, Gomez HF, Ross WA, et al. Pancreaticoduodenectomy After Placement of Endobiliary Metal Stents. J Gastrointest Surg 2005;9:1094–1105.
35. Sanders MK, El Hajj II, Slivka A, et al Placement of Self-Expanding Metal Biliary Stents (SEMS) in Patients with Resectable Pancreatic Cancer [Abstract]. Gastrointest Endosc 2009; 69(5)
36. Kahaleh M, Brock A, Conaway MR, et al. Covered self‒expandable metal stents in pancreatic malignancy regardless of resectability: a new concept validated by a decision analysis. Endoscopy 2007 Apr;39(4):319-24
37. Pop GH, Richter JA, Sauer B, et al. Bridge to surgery using partially covered self-expandable metal stents (PCMS) in malignant biliary stricture: an acceptable paradigm? Surg Endosc 2011 Feb;25(2):613-8
38. Lawrence C , Howell DA, Conklin DE, et al. Delayed pancreaticoduodenectomy for cancer patients with prior ERCP-placed, nonforeshortening, self-expanding metal stents: a positive outcome, Gastrointest Endosc 2006 May;63 (6): 804-807

39. Singal AK, Ross WA, Guturu P, et al. Self-Expanding Metal Stents for Biliary Drainage in Patients with Resectable Pancreatic Cancer: Single-Center Experience with 79 Cases. Dig Dis Sci 2011 Jul 13. [Epub ahead of print]

40. van der Gaag NA, Rauws EAJ, van Eijck CHJ, et al. Preoperative Biliary Drainage for Cancer of the Head of the Pancreas. N Engl J Med 2010;362:129-37.

41. Hariharan D, Saied A, Kocher HM. Analysis of mortality rates for pancreatic cancer across the world. HPB (Oxford). 2008; 10(1): 58–62.

42. American Cancer Society. Cancer Facts and Figures 2012. Atlanta: American Cancer Society; 2012.

43. NCCN Clinical Guidelines in Oncology Pancreatic Adenocarcinoma Version 2.2011, NCCN.org

44. Callery MP, Chang KJ, Fishman EK, Talamonti MS, William Traverso L, Linehan DC. Pretreatment assessment of resectable and borderline resectable pancreatic cancer: expert consensus statement. Ann Surg Oncol. 2009 Jul;16(7):1727-33. Epub 2009 Apr 24.

45. Wong JC, Lu DS. Staging of pancreatic adenocarcinoma by imaging studies. Clin Gastroenterol Hepatol. 2008 Dec;6(12):1301-8. Epub 2008 Sep 27. Review.

46. House MG, Yeo CJ, Cameron JL, Campbell KA, Schulick RD, Leach SD, Hruban RH, Horton KM, Fishman EK, Lillemoe KD. Predicting resectability of periampullary cancer with three-dimensional computed tomography. J Gastrointest Surg. 2004 Mar-Apr;8(3):280-8.

47. Olivié D, Lepanto L, Billiard JS, Audet P, Lavallée JM. Predicting resectability of pancreatic head cancer with multi-detector CT. Surgical and pathologic correlation. JOP. 2007 Nov 9;8(6):753-8.

48. Schima W, Ba-Ssalamah A, Goetzinger P, Scharitzer M, Koelblinger C. State-of-the-art magnetic resonance imaging of pancreatic cancer. Top Magn Reson Imaging. 2007 Dec;18(6):421-9. Review.

49. NCCN Clinical Guidelines in Oncology Pancreatic Adenocarcinoma Version 2.2012, NCCN.org

50. Rösch T, Braig C, Gain T, Feuerbach S, Siewert JR, Schusdziarra V, Classen M. Staging of pancreatic and ampullary carcinoma by endoscopic ultrasonography. Comparison with conventional sonography, computed tomography, and angiography. Gastroenterology. 1992 Jan;102(1):188-99.

51. Varadarajulu S, Wallace MB. Applications of endoscopic ultrasonography in pancreatic cancer. Cancer Control. 2004 Jan-Feb;11(1):15-22. Review.

52. Ahmed SI, Bochkarev V, Oleynikov D, Sasson AR. Patients with pancreatic adenocarcinoma benefit from staging laparoscopy. J Laparoendosc Adv Surg Tech A. 2006 Oct;16(5):458-63.

53. Warshaw AL, Gu ZY, Wittenberg J, Waltman AC. Preoperative staging and assessment of resectability of pancreatic cancer. Arch Surg. 1990 Feb;125(2):230-3.

54. Katz MH, Hwang R, Fleming JB, Evans DB. Tumor-node-metastasis staging of pancreatic adenocarcinoma. CA Cancer J Clin. 2008 Mar-Apr;58(2):111-25. Epub 2008 Feb 13. Review.
55. Edge SB, Byrd DR, Compton CC, et al., eds. Exocrine and endocrine pancreas. AJCC Cancer Staging Manual. 7th ed. New York, NY: Springer, 2010, pp 241-9.
56. Tseng JF, Tamm EP, Lee JE, et al. Venous resection in pancreatic cancer surgery. Best Pract Res Clin Gastroenterol. 2006;20(2):349–364.
57. Bonin EA, Baron TH. Preoperative biliary stents in pancreatic cancer. J Hepatobiliary Pancreat Sci. 2011 Sep;18(5):621-9. Review.
58. Li D, Xie K, Wolff R, Abbruzzese JL. Pancreatic cancer. The Lancet 2004; 363 (9414):1049-1057.
59. Gumbs AA, Rodriguez Rivera AM, Milone L, Hoffman JP. Laparoscopic pancreatoduodenectomy: a review of 285 published cases. Ann Surg Oncol. 2011 May;18(5):1335-41. Epub 2011 Jan 5. Review.
60. Hidalgo M. Pancreatic cancer. N Engl J Med. 2010;362:1605–1617.
61. Boulay BR, Gardner TB, Gordon SR. Occlusion rate and complications of plastic biliary stent placement in patients undergoing neoadjuvant chemoradiotherapy for pancreatic cancer with malignant biliary obstruction. J Clin Gastroenterol 2010 Jul;44(6):452-5.
62. Moss AC, Morris E, Leyden J, MacMathuna P. Malignant distal biliary obstruction: a systematic review and meta-analysis of endoscopic and surgical bypass results. Cancer Treat Rev. 2007 Apr;33(2):213-21. Epub 2006 Dec 8. Review.

12. Appendices: Sponsor Required Protocol Sections

12.1. *Data Management*

12.1.1. Data Collection, Processing, and Review

Subject data will be recorded in a limited access secure electronic data capture (EDC) system.

The clinical database will reside on a production server. All changes made to the clinical data will be captured in an electronic audit trail and available for review by Boston Scientific Corporation (BSC) or its representative. The associated software and database have been designed to meet regulatory compliance for deployment as part of a validated system compliant with laws and regulations applicable to the conduct of clinical studies pertaining to the use of electronic records and signatures. Database backups are performed regularly.

The Investigator provides his/her electronic signature on the appropriate electronic case report forms (eCRFs) in compliance with local regulations. A written signature on printouts of the eCRFs must also be provided if required by local regulation. Changes to data previously submitted to the sponsor require a new electronic signature by the Investigator acknowledging and approving the changes.

Visual and/or electronic data review will be performed to identify possible data discrepancies. Manual and/or automatic queries will be created in the EDC system and will be issued to the site for appropriate response. Site staff will be responsible for resolving all queries in the database.

12.1.2. Data Retention

The Investigator will maintain, at the investigative site, in original format all essential study documents and source documentation that support the data collected on the study subjects in compliance with ICH/GCP guidelines. Documents must be retained for at least 2 years after the last approval of a marketing application or until at least 2 years have elapsed since the formal discontinuation of the clinical investigation of the product. These documents will be retained for a longer period of time by agreement with BSC or in compliance with other local regulations. It is BSC's responsibility to inform the Investigator when these documents no longer need to be maintained. The Investigator will take measures to ensure that these essential documents are not accidentally damaged or destroyed. If for any reason the Investigator withdraws responsibility for maintaining these essential documents, custody must be transferred to an individual who will assume responsibility and BSC must receive written notification of this custodial change.

12.2. *Amendments*

If a protocol revision is necessary which affects the rights, safety or welfare of the subject or scientific integrity of the data, an amendment is required. Appropriate approvals (e.g., IRB/EC/FDA/CA) of the revised protocol must be obtained prior to implementation.

12.3. *Deviations*

An Investigator must not make any changes or deviate from this protocol, except to protect the life and physical well-being of a subject in an emergency. An investigator shall notify the sponsor and the reviewing IRB/EC of any deviation from the investigational plan to protect the life or physical well-being of a subject in an emergency, and those deviations which affect the scientific integrity of the clinical investigation. Such notice shall be given as soon as possible, but no later than 5 working days after the emergency occurred, or per prevailing local requirements, if sooner than 5 working days.

All deviations from the investigational plan, with the reason for the deviation and the date of occurrence, must be documented and reported to the sponsor using the Protocol Deviation EDC CRF. Sites may also be required to report deviations to the IRB/EC, per local guidelines and government regulations.

Deviations will be reviewed and evaluated on an ongoing basis and, as necessary, appropriate corrective and preventive actions (including notification, center re-training, or discontinuation) will be put into place by the sponsor.

12.4. *Device/Equipment Accountability*

There are no investigational devices used in this study. The WallFlex Biliary Fully Covered and Uncovered Stent Systems are available for commercial use in the geographic areas in

which this clinical study is taking place; therefore, there is no requirement for device accountability for the purposes of this study. Device lot information must be maintained in the subject's medical record and recorded on the appropriate case report form.

Any individual country/region requirements that depart from the aforementioned will be implemented on a case-by-case basis.

12.5. *Compliance*

12.5.1. Statement of Compliance

This study will be conducted in accordance with relevant sections of the International Standard (ISO) 14155: Clinical Investigation of Medical devices for Human Subjects – Good Clinical Practice, the relevant parts of the ICH Guidelines for Good Clinical Practices, ethical principles that have their origins in the Declaration of Helsinki, and pertinent individual country laws and regulations. The study will not begin until the required approval/favorable opinion from the EC and/or regulatory authority has been obtained, if appropriate. Any additional requirements imposed by the EC or regulatory authority shall be followed, if appropriate.

12.5.2. Investigator Responsibilities

The Principal Investigator of an investigational center is responsible for ensuring that the study is conducted in accordance with the Clinical Study Agreement, the investigational plan/protocol, ISO 14155, ethical principles that have their origins in the Declaration of Helsinki, any conditions of approval imposed by the reviewing IRB/EC, and prevailing local and/or country laws and/or regulations, whichever affords the greater protection to the subject.

The Principal Investigator's responsibilities include, but are not limited to, the following.

- Prior to beginning the study, sign the Investigator Agreement and Protocol Signature page documenting his/her agreement to conduct the study in accordance with the protocol.
- Provide his/her qualifications and experience to assume responsibility for the proper conduct of the study and that of key members of the center team through up-to-date curriculum vitae or other relevant documentation and disclose potential conflicts of interest, including financial, that may interfere with the conduct of the clinical study or interpretation of results.
- Make no changes in or deviate from this protocol, except to protect the life and physical well-being of a subject in an emergency; document and explain any deviation from the approved protocol that occurred during the course of the clinical investigation.
- Create and maintain source documents throughout the clinical study and ensure their availability with direct access during monitoring visits or audits; ensure that all clinical-investigation-related records are retained per requirements.
- Ensure the accuracy, completeness, legibility, and timeliness of the data reported to the sponsor in the CRFs and in all required reports.

- Record, report, and assess (seriousness and relationship to the device/procedure) every adverse event and observed device deficiency.
- Report to BSC, per the protocol requirements, all SAEs and device deficiencies that could have led to a SADE.
- Report to the IRB/EC and regulatory authorities any SAEs and device deficiencies that could have led to a SADE, if required by the national regulations or this protocol or by the IRB/EC, and supply BSC with any additional requested information related to the safety reporting of a particular event.
- Maintain the device accountability records and control of the device, ensuring that the investigational device is used only by authorized/designated users and in accordance with this protocol and instructions/directions for use.
- Allow the sponsor to perform monitoring and auditing activities, and be accessible to the monitor and respond to questions during monitoring visits.
- Allow and support regulatory authorities and the IRB/EC when performing auditing activities.
- Ensure that informed consent is obtained in accordance with this protocol and local IRB/EC requirements.
- Provide adequate medical care to a subject during and after a subject’s participation in a clinical study in the case of adverse events, as described in the Informed Consent Form (ICF).
- Inform the subject of the nature and possible cause of any adverse events experienced.
- As applicable, provide the subject with necessary instructions on proper use, handling, storage, and return of the investigational device when it is used/operated by the subject.
- Inform the subject of any new significant findings occurring during the clinical investigation, including the need for additional medical care that may be required.
- Provide the subject with well-defined procedures for possible emergency situations related to the clinical study, and make the necessary arrangements for emergency treatment, including decoding procedures for blinded/masked clinical investigations, as needed.
- Ensure that clinical medical records are clearly marked to indicate that the subject is enrolled in this clinical study.
- Ensure that, if appropriate, subjects enrolled in the clinical investigation are provided with some means of showing their participation in the clinical investigation, together with identification and compliance information for concomitant treatment measures (contact address and telephone numbers shall be provided).
- Inform, with the subject’s approval or when required by national regulations, the subject’s personal physician about the subject’s participation in the clinical investigation.
- Make all reasonable efforts to ascertain the reason(s) for a subject’s premature withdrawal from clinical investigation while fully respecting the subject’s rights.

- Ensure that an adequate investigation site team and facilities exist and are maintained and documented during the clinical investigation.
- Ensure that maintenance and calibration of the equipment relevant for the assessment of the clinical investigation is appropriately performed and documented, where applicable.

12.5.3. Delegation of Responsibility

When specific tasks are delegated by an investigator, included but not limited to conducting the informed consent process, the investigator is responsible for providing appropriate training and adequate supervision of those to whom tasks are delegated. The investigator is accountable for regulatory violations resulting from failure to adequately supervise the conduct of the clinical study.

12.6. *Institutional Review Board/ Ethics Committee*

Prior to gaining Approval-to-Enroll status, the investigational center will provide to the sponsor documentation verifying that their IRB/EC is registered or that registration has been submitted to the appropriate agency, as applicable according to national/regulatory requirements.

A copy of the written IRB/EC and/or competent authority approval of the protocol (or permission to conduct the study) and Informed Consent Form, must be received by the sponsor before recruitment of subjects into the study and shipment of investigational product/equipment. Prior approval must also be obtained for other materials related to subject recruitment or which will be provided to the subject.

Annual IRB/EC approval and renewals will be obtained throughout the duration of the study as required by local/country or IRB/EC requirements. Copies of the Investigator's reports and the IRB/EC continuance of approval must be provided to the sponsor.

12.7. *Sponsor Responsibilities*

All information and data sent to BSC concerning subjects or their participation in this study will be considered confidential by BSC. Only authorized BSC personnel or a BSC representative will have access to these confidential records. Authorized regulatory personnel have the right to inspect and copy all records pertinent to this study. Study data collected during this study may be used by BSC for the purposes of this study, publication, and to support future research and/or other business purposes. All data used in the analysis and reporting of this study will be without identifiable reference to specific subject name.

Boston Scientific will keep subjects' health information confidential in accordance with all applicable laws and regulations. Boston Scientific may use subjects' health information to conduct this research, as well as for additional purposes, such as overseeing and improving the performance of its device, new medical research and proposals for developing new medical products or procedures, and other business purposes. Information received during the study will not be used to market to subjects; subject names will not be placed on any mailing lists or sold to anyone for marketing purposes.

12.8. *Insurance*

Where required by local/country regulation, proof and type of insurance coverage, by BSC for subjects in the study will be obtained.

12.9. *Monitoring*

Monitoring will be performed during the study to assess continued compliance with the protocol and applicable regulations. In addition, the monitor verifies that study records are adequately maintained, that data are reported in a satisfactory manner with respect to timeliness, adequacy, and accuracy and that the Investigator continues to have sufficient staff and facilities to conduct the study safely and effectively. The Investigator/institution guarantees direct access to original source documents by BSC personnel, their designees and appropriate regulatory authorities.

The study may also be subject to a quality assurance audit by BSC or its designees, as well as inspection by appropriate regulatory authorities. It is important that the Investigator and relevant study personnel are available during on-site monitoring visits or audits and that sufficient time is devoted to the process.

12.10. *Informed Consent*

Subject participation in this clinical study is voluntary. Informed Consent is required from all subjects or their legally authorized representative. The Investigator is responsible for ensuring that Informed Consent is obtained prior to the use of any investigational devices, study-required procedures and/or testing, or data collection.

The obtaining and documentation of Informed Consent must be in accordance with the principles of the Declaration of Helsinki, ISO 14155, any applicable national regulations, and local Ethics Committee and/or Regulatory authority body, as applicable. The ICF must be approved by the center's IRB/EC, or central IRB, if applicable.

Boston Scientific will provide a study-specific template of the ICF to investigators participating in this study. The ICF template may be modified to meet the requirements of the investigative center's IRB/EC. Any modification requires approval from BSC prior to use of the form. The ICF must be in a language understandable to the subject and if needed, BSC will assist the center in obtaining a written consent translation. Translated consent forms must also have IRB/EC approval prior to their use. Privacy language shall be included in the body of the form or as a separate form as applicable.

The process of obtaining Informed Consent shall:

- be conducted by the Principal Investigator or designee authorized to conduct the process,
- include a description of all aspects of the clinical study that are relevant to the subject's decision to participate throughout the clinical study,
- avoid any coercion of or undue influence of subjects to participate,
- not waive or appear to waive subject's legal rights,

- use native language that is non-technical and understandable to the subject or his/her legal representative,
- provide ample time for the subject to consider participation and ask questions if necessary,
- ensure important new information is provided to new and existing subjects throughout the clinical study.

The ICF shall always be signed and personally dated by the subject or legal representative and by the investigator or an authorized designee responsible for conducting the informed consent process. If a legal representative signs, the subject shall be asked to provide informed consent for continued participation as soon as his/her medical condition allows. The original signed ICF will be retained by the center and a copy of the signed and dated document and any other written information must be given to the person signing the form.

Failure to obtain subject consent will be reported by BSC to the applicable regulatory body according to their requirements (e.g., FDA requirement is within 5 working days of learning of such an event). Any violations of the informed consent process must be reported as deviations to the sponsor and local regulatory authorities (e.g. IRB/EC), as appropriate.

If new information becomes available that can significantly affect a subject's future health and medical care, that information shall be provided to the affected subject(s) in written form via a revised ICF or, in some situations, enrolled subjects may be requested to sign and date an addendum to the ICF. In addition to new significant information during the course of a study, other situations may necessitate revision of the ICF, such as if there are amendments to the protocol, a change in Principal Investigator, administrative changes, or following annual review by the IRB/EC. The new version of the ICF must be approved by the IRB/EC. Boston Scientific approval is required if changes to the revised ICF are requested by the center’s IRB/EC. The IRB/EC will determine the subject population to be re-consented.

12.11. *Committees*

12.11.1. Safety Monitoring Process

An independent data review (IDR) board comprised of physician experts in surgery and gastroenterological endoscopy not participating in the clinical study and with no affiliation with BSC, will provide external oversight and review for potential safety concerns.

During the course of the clinical study, the IDR board will periodically review aggregate accumulating safety data to monitor for the incidence of major adverse events and other trends.

Any IDR recommendations for clinical study modification or termination because of concerns over subject safety or issues relating to data monitoring or quality control will be submitted in writing to BSC for consideration.

12.12. *Suspension or Termination*

12.12.1. Premature Termination of the Study

Boston Scientific Corporation reserves the right to terminate the study at any stage but intends to exercise this right only for valid scientific or administrative reasons and reasons related to protection of subjects. Investigators, associated IRBs/ECs, and regulatory authorities, as applicable, will be notified in writing in the event of study termination.

12.12.2. Criteria for Premature Termination of the Study

Possible reasons for premature study termination include, but are not limited to, the following.

- The occurrence of unanticipated adverse device effects that present a significant or unreasonable risk to subjects enrolled in the study.
- An enrollment rate far below expectation that prejudices the conclusion of the study.
- A decision on the part of Boston Scientific to suspend or discontinue development of the device.

12.12.3. Termination of Study Participation by the Investigator or Withdrawal of IRB/ EC Approval

Any investigator, or IRB/ EC in the Preoperative Biliary Drainage RCT Study may discontinue participation in the study or withdrawal approval of the study, respectively, with suitable written notice to Boston Scientific. Investigators, associated IRBs/ECs, and regulatory authorities, as applicable, will be notified in writing in the event of these occurrences.

12.12.4. Requirements for Documentation and Subject Follow-up

In the event of premature study termination a written statement as to why the premature termination has occurred will be provided to all participating centers by Boston Scientific. The IRB/EC and regulatory authorities, as applicable, will be notified. Detailed information on how enrolled subjects will be managed thereafter will be provided.

In the event an IRB or EC terminates participation in the study, participating investigators, associated IRBs/ECs, and regulatory authorities, as applicable, will be notified in writing. Detailed information on how enrolled subjects will be managed thereafter will be provided by Boston Scientific.

In the event an investigator terminates participation in the study, study responsibility will be transferred to a co-investigator, if possible. In the event there are no opportunities to transfer investigator responsibility; detailed information on how enrolled subjects will be managed thereafter will be provided by Boston Scientific.

The investigator must return all documents and investigational product to Boston Scientific, unless this action would jeopardize the rights, safety, or welfare of the subjects.

12.12.5. Criteria for Suspending/Terminating a Study Center

Boston Scientific Corporation reserves the right to stop the inclusion of subjects at a study center at any time after the study initiation visit if no subjects have been enrolled for a period beyond 2 months after center initiation, or if the center has multiple or severe protocol violations/noncompliance without justification and/or fails to follow remedial actions.

In the event of termination of investigator participation, all study devices and testing equipment, as applicable, will be returned to BSC unless this action would jeopardize the rights, safety or well-being of the subjects. The IRB/EC and regulatory authorities, as applicable, should be notified. All subjects enrolled in the study at the center will continue to be followed per protocol. The Principal Investigator at the center must make provision for these follow-up visits unless BSC notifies the investigational center otherwise.

12.13. *Publication Policy*

In accordance with the Corporate Policy on the Conduct of Human Subject Research, BSC requires disclosure of its involvement as a sponsor or financial supporter in any publication or presentation relating to a BSC study or its results. In accordance with the Corporate Policy for the Conduct of Human Subject Research, BSC will submit study results for publication (regardless of study outcome) following the conclusion or termination of the study. Boston Scientific Corporation adheres to the Contributorship Criteria set forth in the Uniform Requirements of the International Committee of Medical Journal Editors (ICMJE; http://www.icmje.org). In order to ensure the public disclosure of study results in a timely manner, while maintaining an unbiased presentation of study outcomes, BSC personnel may assist authors and investigators in publication preparation provided the following guidelines are followed.

- All authorship and contributorship requirements as described above must be followed.
- BSC involvement in the publication preparation and the BSC Publication Policy should be discussed with the Coordinating Principal Investigator(s) and/or Executive/Steering Committee at the onset of the project.
- The First and Senior authors are the primary drivers of decisions regarding publication content, review, approval, and submission.

12.14. *Definitions of complication criteria (per van der Gaag article):*

<u>Specific PBD (ERCP, PTC) related:</u>

- **Acute pancreatitis** Abdominal pain and a serum concentration of pancreatic enzymes (amylase or lipase) three or more times the upper limit of normal, that required more than one night of hospitalization
- **Acute cholecystitis** No suggestive clinical or radiographic signs of acute cholecystitis before the procedure and if emergency cholecystectomy is subsequently required
- **Perforation** Retroperitoneal or bowel-wall perforation documented by any radiographic technique or direct visual evidence

- **Stent Occlusion** Recurring obstructive jaundice with necessary stent replacement

Specific surgery related:

- **Pancreaticojejunostomy leakage** Drain output of any measurable volume of fluid on or after postoperative day 3 with an amylase content greater than 3 times the serum amylase activity, graded according to clinical course (ISGPS grade A, B, C), or direct visual evidence of defect at anastomosis
- **Delayed gastric emptying** Gastric stasis requiring nasogastric intubation for 10 days or more, or the inability to tolerate a regular (solid) diet on or before the fourteenth postoperative day, not due to sequelae of intra-abdominal complications (i.e. abscess, anastomotic leakage)
- **Biliary leakage** Bilirubin in abdominal drain or dehiscence found at laparotomy
- **Gastro/-duodenojejunostomy leakage** Conclusive radiographic or direct visual evidence of a defect of the anastomosis
- **Intra-abdominal abscess formation** Intra-abdominal fluid collection with positive cultures identified by ultrasonography or computed tomography, associated with persistent fever and elevations of white blood cells
- **Wound infection** Requiring intervention otherwise considered as minor complication
- **Portal Vein Thrombosis** Conclusive radiologic evidence of thrombosis

Following either procedure:

- **Cholangitis** Elevation in temperature more than 38°C, thought to have a biliary cause, without concomitant evidence of acute cholecystitis, requiring intervention
- **Hemorrhage** Bleeding after the index procedure requiring transfusion of ≥4 units of packed cells within a 24-hour period, or leading to relaparotomy/intervention
- **(Emergency) (re)laparotomy** Any (other) reason following either preoperative biliary drainage or another surgical procedure
- **Pneumonia** Pulmonary infection with radiological confirmation and requiring antibiotic treatment
- **Mortality** In-hospital death, due to protocol complications or any cause, including progression of disease, within the study period

Summary of changes to the protocol since the original version

The following deletions (strikethrough) and additions (underlined) were made from the original protocol to the final version:

Objective(s): To demonstrate that preoperative biliary drainage using self-expanding metal stents (SEMS) ~~improves~~ <u>does not negatively impact</u> overall surgical outcomes in patients undergoing pancreaticoduodenectomy for treatment of pancreatic or periampullary cancer.

Test Device: The WallFlex Biliary stents are indicated for use in the palliative treatment of biliary strictures produced by malignant neoplasms~~; the~~ <u>and relief of malignant biliary obstruction prior to surgery</u>
<u>Note: The</u> WallFlex <u>Biliary</u> FC stent is also indicated for the treatment of benign biliary strictures per CE Mark <u>or other local regulations</u>

Planned Number of Centers ~~/ Countries~~: ~~7 – 12~~ <u>Up to 20</u> sites ~~Countries: Australia, Belgium, China, France, Germany, India, Italy, Japan (this list of countries may change)~~ <u>world-wide</u>

Study Devices: The WallFlex Biliary RX Fully Covered and the WallFlex Biliary RX Uncovered Stent Systems are indicated for use in the palliative treatment of biliary strictures produced by malignant neoplasms and <u>relief of malignant biliary obstruction prior to surgery</u>; the WallFlex Biliary RX Fully Covered Stent System is also indicated for treatment of benign biliary strictures, per CE Mark <u>or other local regulations</u>.

Study Visits:
Pre-Operative Visit (Week 2 <u>+/- 3 days</u>)
Post-Operative Follow-up Visit (30 days Post-Surgery <u>+/- 7 days</u>
Long Term Follow-up Visit (120 days – 150 days post randomization <u>+/- 14 days</u>

Anticipated Adverse Device Effects with Use of WallFlex Biliary Stent:
- Bile duct <u>or duodenum</u> ulceration
- <u>Stent fracture</u>
- <u>Hepatic abscess</u>

Boston Scientific Device Deficiencies: ~~Device failures and malfunctions should also be documented in the subject's medical record.~~

Original statistical analysis plan

Statistical Analysis Plan

Version AA

Randomized Multi-Center Study Comparing No Drainage to Preoperative Biliary Drainage Using Metal Stents in Patients with Resectable Pancreatic or Periampullary Cancer

Preoperative Biliary Drainage RCT

E7059

CONFIDENTIAL

DO NOT COPY OR DISTRIBUTE WITHOUT WRITTEN PERMISSION

Revision History

Version AA - Initial Release

Original Release: July 3, 2012

Revision History

Revision Number/Release Date	Section	Change	Reason for Change

TABLE OF CONTENTS

1 PROTOCOL SUMMARY 98

2 INTRODUCTION 102

3 ENDPOINT ANALYSIS 102

3.1 Primary Endpoint 102

3.1.1 Hypotheses 103

3.1.2 Sample Size 103

3.1.3 Statistical Methods 103

4 GENERAL STATISTICAL METHODS 104

4.1 Analysis Sets 104

5 ADDITIONAL DATA ANALYSES 104

5.1 Secondary Endpoints 104

5.2 Baseline Data 105

5.3 Post-Procedure Endpoints 105

5.4 Subgroup Analyses 105

5.5 Justification of Pooling 105

5.6 Multivariable Analysis 105

5.7 Analysis of LFT's and Obstruction symptoms 105

5.8 Analysis of Impact of Adverse Events on Endpoints 106

5.9 Baseline Tumor Stage vs. Pathology Tumor Stage 106

5.10 Changes to Planned Analyses 106

6 VALIDATION 106

7 PROGRAMMING CONSIDERATIONS 106

7.1 Statistical Software 106

7.2 Format of output 106

7.3 Rules and Definitions 106

1 PROTOCOL SUMMARY

Full Title	**Randomized Multi-Center Study Comparing No Drainage to Preoperative Biliary Drainage Using Metal Stents in Patients with Resectable Pancreatic or Periampullary Cancer**
Short Title	Preoperative Biliary Drainage RCT
Objective(s)	To demonstrate that preoperative biliary drainage using self-expanding metal stents (SEMS) improves overall surgical outcomes in patients undergoing pancreaticoduodenectomy for treatment of pancreatic or periampullary cancer.
Test Device	<u>Devices</u>:<br>WallFlex Biliary RX Fully Covered stent<br>Note: WallFlex Biliary RX Uncovered stents may be used in in some cases as outlined in the protocol<br><u>Cleared Indication</u>:<br>The WallFlex Biliary stents are indicated for use in the palliative treatment of biliary strictures produced by malignant neoplasms; the WallFlex FC stent is also indicated for the treatment of benign biliary strictures per CE Mark.
Study Design	• Prospective, multi-center, randomized, post-market (on label)<br>• Patients will be block randomized at baseline in a 1:1 ratio between **Group 1:** No Pre-Operative Biliary Drainage and **Group 2:** Pre-Operative Biliary Drainage with a SEMS.
Planned Number of Subjects	**294 total number of subjects:**<br>• Group 1: 147<br>• Group 2: 147
Planned Number of Centers / Countries	7-12 sites<br><br>Countries: Australia, Belgium, China, France, Germany, India, Italy, Japan (this list of countries may change)
Primary Endpoint	Serious pre-operative, operative and post-operative adverse events to 120 days post randomization or to 30 days post-surgery, whichever comes last
**Secondary Endpoints**	1. Adverse events: rate, severity, seriousness, relatedness to stent or endoscopic or surgical procedure, impact on time of surgery,

	length of hospitalization and ICU stay<br>2. Time to surgery<br>3. Curative Intent Surgery details pertaining to intraoperative assessment of resectability, surgical resection and reconstruction techniques<br>4. Intraoperative blood loss and blood transfusions, duration of surgery<br>5. Biliary obstructive symptoms assessment (Week 2, surgery/transition to palliation, 30 day post-operative follow up and 120 day follow up)<br>6. Improvement of LFT levels as relative to baseline (Week 2, surgery/transition to palliation, 30 day post-operative follow up and 120 day follow up)<br>7. Stent placement success: ability to deploy the stent in satisfactory position across the stricture **(Group 2)**<br>8. Stent removal success: successful SEMS removal, either *en bloc* at time of surgery or endoscopically prior to surgery without stent removal related SAEs<br>9. Number, type, reason and timing of biliary re-interventions<br>10. Number and duration of hospital and ICU admissions
**Follow-up Schedule and Assessments**	• **Screening:** Count all consecutive patients seen at investigational site presenting with biliary obstructive symptoms and suspicion of pancreatic cancer, distal common bile duct cholangiocarcinoma or peri-ampullary cancer. Of those, a subset will be invited to participate in trial: Informed Consent (enrollment) and Eligibility Criteria Assessment<br>• **Baseline Visit (Day 0):** Demographics, Medical History, Tumor Diagnosis, Staging and Characteristics, Assessment of Biliary Obstructive Symptoms, Laboratory Tests, Randomization<br>• **Stent Placement Procedure Visit (Group 2 only, Day 0):** Stent Details, Procedure Details, Adverse Events (AEs)<br>• **Pre-Operative Visit (Week 2):** Assessment of Biliary Obstructive Symptoms, Laboratory Tests**,** AEs<br>***Group 1 (No Pre-Operative Biliary Drainage):*** Patients will undergo resection per institutional standard of practice. Time to surgery is not to exceed 4 weeks unless there is a need to delay planned surgery due to a complication, biliary obstructive symptoms, decline in patient physiologic status, or other factors that preclude surgery.<br>***Group 2 (Pre-Operative Biliary Drainage with a SEMS):*** Patients will undergo resection after resolution of jaundice is achieved

	(defined as Bilirubin below 100μmol per liter), or at 4 weeks, whichever comes first.<br>• **Biliary Reintervention Visit (as needed):** Timing, Reason for Biliary Reintervention, Type of Biliary Reintervention, AEs<br>Group 1 patients who fail their treatment algorithm by requiring biliary drainage prior to surgery will receive a study SEMS.<br>Group 2 patients who require re-stenting or placement of a stent for a new stricture, will receive a study SEMS.<br>• **Curative Intent Surgery: Assessment of Biliary Obstructive Symptoms,** Laboratory Tests**,** Operative Details, Stent Removal, Blood Loss, Intra- and Post-Operative Transfusion, Post-Operative Course, Specimen Pathology, AEs<br>• **Transition to Palliative Management Visit (as needed):** Assessment of Biliary Obstructive Symptoms, **Laboratory Tests**, AEs<br>• **Post-Operative Follow up** Visit **(**30 days Post-Surgery, if applicable): Assessment of Biliary Obstructive Symptoms, Laboratory Tests, AEs<br>• **Long Term Follow-up Visit (120 days--150 days post randomization):** Assessment of Biliary Obstructive Symptoms, Laboratory Tests, AEs<br>Patients who undergo surgery as planned will be evaluated at 30 days post-surgery and will be followed up to 120 days after randomization.<br>Patients with delay in planned surgery may require follow up longer than 120 days to allow for a 30 day post-surgery follow up visit but follow up is not to exceed 150 days post-randomization.<br>Patients with a delay in planned surgery whose rescheduled surgery is unable to occur within 120 days will be followed up to 120 days after randomization.<br>Patients who do not undergo surgery as planned due to conversion to palliative management or patient choice will be followed up to 120 days after randomization. If patient's course of treatment requires placement of a biliary stent, stenting will be done per standard of care at each institution. If a metal stent is required, a study WallFlex Biliary FC stent will be placed.
Key Inclusion Criteria	1. Age 18 or older<br>2. Willing and able to comply with the study procedures and provide written informed consent to participate in the study<br>3. Diagnosis of probable pancreatic cancer, distal common bile duct

	(CBD) cholangiocarcinoma and other periampullary cancers (histology not required)<br>4. Biliary obstructive symptoms or signs<br>5. Bilirubin level at/above 100 μmol per liter (5.8 mg/dL)<br>6. Distal biliary obstruction consistent with pancreatic cancer, distal CBD cholangiocarcinoma or other periampullary malignancy<br>7. Location of distal biliary obstruction is such that it would allow the proximal end of a stent to be positioned at least 2 cm from the hilum<br>8. Patients deemed as resectable by pancreatic protocol CT or MRI<br>9. Surgical candidate per pancreatobiliary surgeon after multi-disciplinary discussion<br>10. Surgery intent within 4 weeks<br>11. Endoscopic and surgical treatment to be provided by same team
Key Exclusion Criteria	1. Biliary strictures caused by confirmed benign tumors<br>2. Biliary strictures caused by malignancies other than pancreatic, cancer, distal CBD cholangiocarcinoma and other periampullary cancers<br>3. Surgically altered biliary tract anatomy, not including prior cholecystectomy<br>4. Neoadjuvant chemotherapy for current malignancy<br>5. Palliative indication due to reasons other than surgical candidate status<br>6. Previous biliary drainage by ERCP/PTC<br>7. Patients for whom endoscopic techniques are contraindicated<br>8. Participation in another investigational trial within 90 days<br>9. Pregnancy
Primary Statistical Hypothesis	A literature search of preoperative biliary drainage with self-expanding metal stents in patients with pancreatic or periampullary cancer yielded eight articles with 305 patients.[1-8]<br>A meta-analysis of the probability for pre-operative, operative and peri-operative complications was performed. A rate of 24.2% [95% CI: 13.2%, 37.2%] was calculated using the eight articles.<br><br>Statistical testing will be performed to determine if the rate of complications for the *Pre-Operative Biliary Drainage with SEMS* group is non-inferior to the *No Pre-Operative Biliary Drainage* group. The null hypothesis is

	that the complication rate is inferior in the *Pre-Operative Biliary Drainage with SEMS* versus the *No Pre-Operative Biliary Drainage* group:<br><br>$Ho: \pi_{test} - \pi_{control} \geq \Delta$ (Inferior)<br><br>$Ha: \pi_{test} - \pi_{control} < \Delta$ (Non-inferior)<br><br>where π_{test} and $\pi_{control}$ are the probabilities of having pre-operative, operative and peri-operative complications in the *Pre-Operative Biliary Drainage with SEMS* arm and *No Pre-Operative Biliary Drainage* arm respectively, and Δ is defined as the non-inferiority margin.<br><br>The sample size was calculated for a one-sided 0.050 exact Farrington-Manning test using StatXact 9®. If the P value from the exact Farrington-Manning test is <0.05 then the *Pre-Operative Biliary Drainage with SEMS* group will be considered non-inferior to the *No Pre-Operative Biliary Drainage* group. The expected probability of complications in the *Pre-Operative Biliary Drainage with SEMS* arm and *No Pre-Operative Biliary Drainage* arm is 37.2%, which was taken from the upper limit of the 95% CI from the meta-analysis described above and from the only available Level 1 study comparing no drainage to preoperative stenting in which the complication rate was reported to be 39% in the no drainage arm.[9] The non-inferiority margin (Δ) is 15%. Given these assumptions and a one-sided 5% significance level, 2 x 132 = 264 subjects will provide 80% power to reject the null hypothesis, that the *Pre-Operative Biliary Drainage with SEMS* group is inferior to the *No Pre-Operative Biliary Drainage* group.<br><br>To compensate for possible loss of subjects after enrollment and complete assessment of inclusion/exclusion criteria, an additional 10% of subjects will be enrolled, for a total of 2 x 147 = 294 subjects.

2 INTRODUCTION

This statistical plan addresses the planned analyses for the WallFlex Biliary Preoperative Biliary Drainage RCT based on the protocol dated 17 October 2012, Version AC. All of the specified analyses may not be provided in reports to Competent Authorities but may be used for scientific presentations and/or manuscripts. The primary analysis will be based on the data through 120 days post-randomization or 30 days post-surgery whichever comes last.

3 ENDPOINT ANALYSIS

3.1 Primary Endpoint

The rate of serious adverse events is the primary endpoint for the study.

3.1.1 Hypotheses

Statistical testing will be performed to determine if the rate of complications for the Pre-Operative Biliary Drainage with SEMS group is non-inferior to the No Pre-Operative Biliary Drainage group. The null hypothesis is that the complication rate is inferior in the Pre-Operative Biliary Drainage with SEMS versus the No Pre-Operative Biliary Drainage group:

Ho: $\pi_{test} - \pi_{control} \geq \Delta$ (Inferior)

Ha: $\pi_{test} - \pi_{control} < \Delta$ (Non-inferior)

where π_{test} and $\pi_{control}$ are the probabilities of having pre-operative, operative and peri-operative complications in the Pre-Operative Biliary Drainage with SEMS arm and No Pre-Operative Biliary Drainage arm respectively, and Δ is defined as the non-inferiority margin.

3.1.2 Sample Size

A literature search of preoperative biliary drainage with self-expanding metal stents in patients with pancreatic or periampullary cancer yielded 8 articles with 305 patients.[21-24, 27-31]

The following meta-analysis of the probability for pre-operative, operative and post-operative complications was done:

- A rate of 24.2% [95% CI: 13.2%, 37.2%] was calculated using the 8 articles.

The sample size was calculated for a one-sided 0.050 exact Farrington-Manning test using StatXact 9®. If the P value from the exact Farrington-Manning test is <0.05 then the *Pre-Operative Biliary Drainage with SEMS* group will be considered non-inferior to the *No Pre-Operative Biliary Drainage* group. The expected probability of complications in the *Pre-Operative Biliary Drainage with SEMS* arm and *No Pre-Operative Biliary Drainage* arm is 37.2%, which was taken from the upper limit of the 95% CI from the meta-analysis described above and from the only available Level 1 study comparing no drainage to preoperative stenting in which the complication rate was reported to be 39% in the no drainage arm.[9] The non-inferiority margin (Δ) is 15%. Given these assumptions and a one-sided 5% significance level, 2 x 132 = 264 subjects will provide 80% power to reject the null hypothesis, that the *Pre-Operative Biliary Drainage with SEMS* group is inferior to the *No Pre-Operative Biliary Drainage* group.

To compensate for possible loss of subjects after enrollment and complete assessment of inclusion/exclusion criteria, an additional 10% of subjects will be enrolled, for a total of 2 x 147 = 294 subjects.

3.1.3 Statistical Methods

For calculating the rate of serious adverse events after randomization, patients with insufficient follow-up will be excluded from the calculation; that is, only patients that have at least follow-up through the long term visit (120/150 day follow-up) will be included unless the subject has already had a serious adverse event. All patients with a serious adverse will be counted in the analysis. Sensitivity analyses, such as a tipping point analysis will be

performed to assess the impact of the missing data, assuming extremes of either no SAEs or all SAEs for the primary endpoint, if the data support such analysis.

4 GENERAL STATISTICAL METHODS

4.1 Analysis Sets

Primary endpoint and selected secondary endpoints will be done for the following cohorts.

Enrolled Cohort

A subject is considered "enrolled" after signing the study-specific ICF. Subjects who sign the ICF but subsequently do not meet one or more of the eligibility criteria provided in the synopsis above will be considered screen failures and excluded from the study.

Intent-to-Treat Cohort (ITT)

This cohort consists of those "enrolled" subjects who meet all inclusion/exclusion criteria and are subsequently randomized.

Per-Protocol Cohort (PP)

The per-protocol cohort is a subset of the ITT subjects who are treated per protocol after randomization (Group 1 no stent, and Group 2 received a study stent) and who had no major protocol deviations (ICH E9 definitions).

5 ADDITIONAL DATA ANALYSES

5.1 Secondary Endpoints

1. Adverse events: rate, severity, seriousness, relatedness to stent or endoscopic or surgical procedure, impact on time of surgery, length of hospitalization and ICU stay
2. Time to surgery
3. Curative Intent Surgery details pertaining to intraoperative assessment of resectability, surgical resection and reconstruction techniques
4. Intraoperative blood loss and blood transfusions, duration of surgery
5. Biliary obstructive symptoms assessment (Week 2, surgery/transition to palliation, 30 day post-operative follow up and 120 day follow up)
6. Improvement of LFT levels as relative to baseline (Week 2, surgery/transition to palliation, 30 day post-operative follow up and 120 day follow up)
7. Stent placement success: ability to deploy the stent in satisfactory position across the stricture (Group 2)
8. Stent removal success: successful SEMS removal, either *en bloc* at time of surgery or endoscopically prior to surgery without stent removal related SAEs
9. Number, type, reason and timing of biliary re-interventions and time to first re-intervention.

10. Number and duration of hospital and ICU admissions

5.2 Baseline Data

Baseline data will be summarized. Subject demographics, clinical history, risk factors, LFTs, tumor diagnosis, and obstruction symptoms will be summarized using descriptive statistics (e.g., mean, standard deviation, n, minimum, maximum) for continuous variables and frequency statistics for discrete variables.

5.3 Post-Procedure Endpoints

Post-procedure information will be collected at regularly scheduled follow-up examinations as detailed in the clinical study event schedule and will be summarized using descriptive statistics for continuous variables (e.g., mean, standard deviation, n, minimum, maximum) and frequency tables or proportions for discrete variables.

5.4 Subgroup Analyses

The subgroup analyses will include tabulating the primary endpoint and select secondary endpoints by gender.

5.5 Justification of Pooling

The analyses will be presented using pooled data across institutions. An analysis of the poolability will be made using logistic regression for binary outcomes, proportional hazards regression for time-to-event outcomes, or analysis of variance for continuous outcomes, to assess differences between study institutions and to justify pooling data across institutions.

5.6 Multivariable Analysis

Univariate and multivariate analyses will be performed to assess possible predictors of the rate of serious adverse events. Possible predictors may include any but not limited to demographic/baseline data and medical history data. Factors from the univariate model with $p \leq 0.20$ will also be modeled multivariately using a stepwise procedure in a generalized linear model or Cox proportional hazards regression model. The significance thresholds for entry and exit into the model will be set to $p<0.10$.

5.7 Analysis of LFT's and Obstruction symptoms

For analysis of LFT's and obstruction symptoms, a paired t-test and McNemar's Test will be used to test differences from baseline. The data will also be analyzed using a generalized linear model, including treatment group and baseline covariates as predictors. Interactions between time and treatment group will be explored. Other possible predictors may include any but not limited to demographic/baseline data and medical history data. Different correlation structures will be fit to determine the best model fit.

5.8 Analysis of Impact of Adverse Events on Endpoints

For an analysis on the effect of the adverse events impact on time of surgery, length of hospitalization, and ICU stay, subjects with and without AEs will be analyzed to determine if any differences occur. Appropriate testing will be done to determine this, i.e. a 2x2 ANOVA analysis with a treatment interaction.

5.9 Baseline Tumor Stage vs. Pathology Tumor Stage

A frequency table will be conducted to look for the differences between the baseline tumor stage and the pathology tumor stage diagnosis. This table will include all stages including a missing category for both time points.

5.10 Changes to Planned Analyses

Any changes to the planned statistical analyses made prior to performing the analyses will be documented in a Statistical Analysis Plan approved prior to performing the analyses.

6 Validation

All clinical data reports generated per this plan will be validated per Global WI: Clinical Data Reporting Validation.

7 Programming Considerations

7.1 Statistical Software

All statistical analyses will be done using The SAS System software, version 8 or higher (Copyright © 2000 SAS Institute Inc., SAS Campus Drive, Cary, North Carolina 27513, USA. All rights reserved).

7.2 Format of output

Results of analysis will be output programmatically to Word documents from SAS with no manual intervention. All output for the final statistical report will be in the form of a Word document containing tables, figures, graphs, and listings, as appropriate.

7.3 Rules and Definitions

Binary event rates (proportions) will be reported on a per patient basis.

The last follow-up date will be the latest of the following dates for each patient: date of an adverse event, index procedure date, follow-up visit date, any stent procedure/reintervention date, surgery date, stent removal date, and device event date.

Serious Adverse Event will be defined as an adverse event that:

- Led to death
- Led to a serious deterioration in the health of the subject that either resulted in:
  - a life-threatening illness or injury, or
  - a permanent impairment of a body structure or a body function, or
  - in-patient hospitalization or prolonged hospitalization (of an existing hospitalization), or
  - medical or surgical intervention to prevent life-threatening illness or injury or permanent impairment to a body structure or a body function
- Led to fetal distress, fetal death, or a congenital abnormality or birth defect.

Note: Planned hospitalization for a pre-existing condition, or a procedure required by the protocol, without serious deterioration in health, is not considered a serious adverse event.

When calculating rates of adverse events, missing and partial dates will be handled as follows:

**Partial Date Description**	**Action Taken**
Entire onset date is missing	The procedure date will be used for the onset date.
The month and the day of the month are missing but the year is available	January 1 will be used for the month and day of the onset date. However, if the imputed date falls before the procedure date, then the procedure date will be used for the onset date.
Day is missing, but the month and year are available	The 1st will be used as the day of the onset date. However, if the imputed date falls before the procedure date, then the procedure date will be used for the onset date.

Definitions of complication criteria (per van der Gaag article):

<u>Specific PBD (ERCP, PTC) related:</u>

- **Acute pancreatitis** Abdominal pain and a serum concentration of pancreatic enzymes (amylase or lipase) three or more times the upper limit of normal, that required more than one night of hospitalization
- **Acute cholecystitis** No suggestive clinical or radiographic signs of acute cholecystitis before the procedure and if emergency cholecystectomy is subsequently required
- **Perforation** Retroperitoneal or bowel-wall perforation documented by any radiographic technique or direct visual evidence

- **Stent Occlusion** Recurring obstructive jaundice with necessary stent replacement

Specific surgery related:

- **Pancreaticojejunostomy leakage** Drain output of any measurable volume of fluid on or after postoperative day 3 with an amylase content greater than 3 times the serum amylase activity, graded according to clinical course (ISGPS grade A, B, C), or direct visual evidence of defect at anastomosis
- **Delayed gastric emptying** Gastric stasis requiring nasogastric intubation for 10 days or more, or the inability to tolerate a regular (solid) diet on or before the fourteenth postoperative day, not due to sequelae of intra-abdominal complications (i.e. abscess, anastomotic leakage)
- **Biliary leakage** Bilirubin in abdominal drain or dehiscence found at laparotomy
- **Gastro/-duodenojejunostomy leakage** Conclusive radiographic or direct visual evidence of a defect of the anastomosis
- **Intra-abdominal abscess formation** Intra-abdominal fluid collection with positive cultures identified by ultrasonography or computed tomography, associated with persistent fever and elevations of white blood cells
- **Wound infection** Requiring intervention otherwise considered as minor complication
- **Portal Vein Thrombosis** Conclusive radiologic evidence of thrombosis

Following either procedure:

- **Cholangitis** Elevation in temperature more than 38°C, thought to have a biliary cause, without concomitant evidence of acute cholecystitis, requiring intervention
- **Hemorrhage** Bleeding after the index procedure requiring transfusion of ≥4 units of packed cells within a 24-hour period, or leading to relaparotomy/intervention
- **(Emergency) (re)laparotomy** Any (other) reason following either preoperative biliary drainage or another surgical procedure
- **Pneumonia** Pulmonary infection with radiological confirmation and requiring antibiotic treatment
- **Mortality** In-hospital death, due to protocol complications or any cause, including progression of disease, within the study period

Final statistical analysis plan including changes since the original version

Statistical Analysis Plan

Version B

Randomized Multi-Center Study Comparing No Drainage to Preoperative Biliary Drainage Using Metal Stents in Patients with Resectable Pancreatic or Periampullary Cancer

Preoperative Biliary Drainage RCT

E7059

CONFIDENTIAL

DO NOT COPY OR DISTRIBUTE WITHOUT WRITTEN PERMISSION

Revision History

Version B - Initial Release

Original Release: July 3, 2012

Revision History

Revision Number/Release Date	Section	Change	Reason for Change
1/16FEB2021	7.3	Removed instructions for fixing missing dates	Missing dates are not allowed in the database
1/16FEB2021	5.10	Added in survival analysis	At request of clinical added in to define a survival analysis to 120 days
1/16FEB2021	7.1	Added StatXact	Primary endpoint analysis will be done using StatXact not SAS
1/16FEB2021	1	Updated protocol summary	To match current version of protocol
1/16FEB2021	5.4	Added subgroup analysis	Investigators, required prespecified PJ vs. PG subgroup analysis
1/16FEB2021	5.7	Removed language	McNemar's test not necessarily appropriate analysis
1/16FEB2021	5.8	Added "Serious"	Analysis should be about serious AEs not all AEs

TABLE OF CONTENTS

1 PROTOCOL SUMMARY 113

2 INTRODUCTION 10217

3 ENDPOINT ANALYSIS 10217

3.1 Primary Endpoint 10217

3.1.1 Hypotheses 10318

3.1.2 Sample Size 10318

3.1.3 Statistical Methods 10318

4 GENERAL STATISTICAL METHODS 10419

4.1 Analysis Sets 10419

5 ADDITIONAL DATA ANALYSES 10419

5.1 Secondary Endpoints 10419

5.2 Baseline Data 10520

5.3 Post-Procedure Endpoints 10520

5.4 Subgroup Analyses 10520

5.5 Justification of Pooling 10520

5.6 Multivariable Analysis 10520

5.7 Analysis of LFT's and Obstruction symptoms 10520

5.8 Analysis of Impact of Adverse Events on Endpoints 10621

5.9 Baseline Tumor Stage vs. Pathology Tumor Stage 10621

5.10 Changes to Planned Analyses 10621

6 VALIDATION 10621

7 PROGRAMMING CONSIDERATIONS 10621

7.1 Statistical Software 10621

7.2 Format of output 10622

7.3 Rules and Definitions 10622

1 PROTOCOL SUMMARY

Full Title	**Randomized Multi-Center Study Comparing No Drainage to Preoperative Biliary Drainage Using Metal Stents in Patients with Resectable Pancreatic or Periampullary Cancer**
Short Title	Preoperative Biliary Drainage RCT
Objective(s)	To demonstrate that preoperative biliary drainage using self-expanding metal stents (SEMS) does not negatively impact overall surgical outcomes in patients undergoing pancreaticoduodenectomy for treatment of pancreatic or periampullary cancer.
Test Device	<u>Devices</u>:<br>WallFlex Biliary RX Fully Covered stent<br>Note: WallFlex Biliary RX Uncovered stents may be used in some cases as outlined in the protocol<br><u>Cleared Indications</u>:<br>The WallFlex Biliary stents are indicated for use in the palliative treatment of biliary strictures produced by malignant neoplasms and relief of malignant biliary obstruction prior to surgery<br>Note: The WallFlex Biliary FC stent is also indicated for the treatment of benign biliary strictures per CE Mark or other local regulations
Study Design	• Prospective, multi-center, randomized, post-market (on label)<br>• Patients will be block randomized at baseline in a 1:1 ratio between **Group 1:** No Pre-Operative Biliary Drainage and **Group 2:** Pre-Operative Biliary Drainage with a SEMS.
Planned Number of Subjects	**294 total number of subjects:**<br>• Group 1: 147<br>• Group 2: 147
Planned Number of Centers	Up to 20 sites world-wide
Primary Endpoint	Serious pre-operative, operative and post-operative adverse events to 120 days post randomization or to 30 days post-surgery, whichever comes last
**Secondary Endpoints**	**1. Adverse events: rate, severity, seriousness, relatedness to stent**

	**or endoscopic or surgical procedure, impact on time of surgery, length of hospitalization and ICU stay**<br>2. **Time to surgery**<br>3. **Curative Intent Surgery details pertaining to intraoperative assessment of resectability, surgical resection and reconstruction techniques**<br>4. **Intraoperative blood loss and blood transfusions, duration of surgery**<br>5. Biliary obstructive symptoms assessment (Week 2, surgery/transition to palliation, 30 day post-operative follow up and 120 day follow up)<br>6. Improvement of LFT levels as relative to baseline (Week 2, surgery/transition to palliation, 30 day post-operative follow up and 120 day follow up)<br>7. Stent placement success: ability to deploy the stent in satisfactory position across the stricture **(Group 2)**<br>8. **Stent removal success**: successful SEMS removal, either *en bloc* at time of surgery or endoscopically prior to surgery without stent removal related SAEs<br>9. Number, type, reason and timing of biliary re-interventions<br>10. Number and duration of hospital and ICU admissions
**Follow-up Schedule and Assessments**	• **Screening:** Count all consecutive patients seen at investigational site presenting with biliary obstructive symptoms and suspicion of pancreatic cancer, distal common bile duct cholangiocarcinoma or peri-ampullary cancer. Of those, a subset will be invited to participate in trial: Informed Consent (enrollment) and Eligibility Criteria Assessment<br>• **Baseline Visit (Day 0):** Demographics, Medical History, Tumor Diagnosis, Staging and Characteristics, Assessment of Biliary Obstructive Symptoms, Laboratory Tests, Randomization<br>• **Stent Placement Procedure Visit (Group 2 only, Day 0):** Stent Details, Procedure Details, Adverse Events (AEs)<br>• **Pre-Operative Visit (Week 2):** Assessment of Biliary Obstructive Symptoms, Laboratory Tests**,** AEs<br>***Group 1 (No Pre-Operative Biliary Drainage):*** Patients will undergo resection per institutional standard of practice. Time to surgery is not to exceed 4 weeks unless there is a need to delay planned surgery due to a complication, biliary obstructive symptoms, decline in patient physiologic status, or other factors that preclude surgery.<br>***Group 2 (Pre-Operative Biliary Drainage with a SEMS):*** Patients

	will undergo resection after resolution of jaundice is achieved (defined as Bilirubin below 100μmol per liter), or at 4 weeks, whichever comes first.<br>• **Biliary Reintervention Visit (as needed):** Timing, Reason for Biliary Reintervention, Type of Biliary Reintervention, AEs<br>Group 1 patients who fail their treatment algorithm by requiring biliary drainage prior to surgery will receive a study SEMS.<br>Group 2 patients who require re-stenting or placement of a stent for a new stricture, will receive a study SEMS.<br>• **Curative Intent Surgery**: **Assessment of Biliary Obstructive Symptoms**, Laboratory Tests**,** Operative Details, Stent Removal, Blood Loss, Intra- and Post-Operative Transfusion, Post-Operative Course, Specimen Pathology, AEs<br>• **Transition to Palliative Management Visit (as needed):** Assessment of Biliary Obstructive Symptoms, Laboratory Tests, AEs<br>• **Post-Operative Follow up Visit** (**30 days Post-Surgery, if applicable): Assessment of Biliary Obstructive Symptoms**, Laboratory Tests**, AEs**<br>• **Long Term Follow-up Visit (120 days--150 days post randomization): Assessment of Biliary Obstructive Symptoms,** Laboratory Tests**, AEs**<br>Patients who undergo surgery as planned will be evaluated at 30 days post-surgery and will be followed up to 120 days after randomization.<br>Patients with delay in planned surgery may require follow up longer than 120 days to allow for a 30 day post-surgery follow up visit but follow up is not to exceed 150 days post-randomization.<br>Patients with a delay in planned surgery whose rescheduled surgery is unable to occur within 120 days will be followed up to 120 days after randomization.<br>Patients who do not undergo surgery as planned due to conversion to palliative management or patient choice will be followed up to 120 days after randomization. If patient's course of treatment requires placement of a biliary stent, stenting will be done per standard of care at each institution. If a metal stent is required, a study WallFlex Biliary FC stent will be placed.
Key Inclusion Criteria	1. Age 18 or older<br>2. Willing and able to comply with the study procedures and provide written informed consent to participate in the study<br>3. Diagnosis of probable pancreatic cancer, distal common bile duct

	(CBD) cholangiocarcinoma and other periampullary cancers (histology not required)<br>4. Biliary obstructive symptoms or signs<br>5. Bilirubin level at/above 100 μmol per liter (5.8 mg/dL)<br>6. Distal biliary obstruction consistent with pancreatic cancer, distal CBD cholangiocarcinoma or other periampullary malignancy<br>7. Location of distal biliary obstruction is such that it would allow the proximal end of a stent to be positioned at least 2 cm from the hilum<br>8. Patients deemed as resectable by pancreatic protocol CT or MRI<br>9. Surgical candidate per pancreatobiliary surgeon after multi-disciplinary discussion<br>10. Surgery intent within 4 weeks<br>11. Endoscopic and surgical treatment to be provided by same team
Key Exclusion Criteria	1. Biliary strictures caused by confirmed benign tumors<br>2. Biliary strictures caused by malignancies other than pancreatic, cancer, distal CBD cholangiocarcinoma and other periampullary cancers<br>3. Surgically altered biliary tract anatomy, not including prior cholecystectomy<br>4. Neoadjuvant chemotherapy for current malignancy<br>5. Palliative indication due to reasons other than surgical candidate status<br>6. Previous biliary drainage by ERCP/PTC<br>7. Patients for whom endoscopic techniques are contraindicated<br>8. Participation in another investigational trial within 90 days<br>9. Pregnancy
Primary Statistical Hypothesis	A literature search of preoperative biliary drainage with self-expanding metal stents in patients with pancreatic or periampullary cancer yielded eight articles with 305 patients.[1-8]<br>A meta-analysis of the probability for pre-operative, operative and peri-operative complications was performed. A rate of 24.2% [95% CI: 13.2%, 37.2%] was calculated using the eight articles.<br><br>Statistical testing will be performed to determine if the rate of complications for the *Pre-Operative Biliary Drainage with SEMS* group is non-inferior to the *No Pre-Operative Biliary Drainage* group. The null hypothesis is

	that the complication rate is inferior in the *Pre-Operative Biliary Drainage with SEMS* versus the *No Pre-Operative Biliary Drainage* group:<br><br>$Ho: \pi_{test} - \pi_{control} \geq \Delta$ (Inferior)<br><br>$Ha: \pi_{test} - \pi_{control} < \Delta$ (Non-inferior)<br><br>where π_{test} and $\pi_{control}$ are the probabilities of having pre-operative, operative and peri-operative complications in the *Pre-Operative Biliary Drainage with SEMS* arm and *No Pre-Operative Biliary Drainage* arm respectively, and Δ is defined as the non-inferiority margin.<br><br>The sample size was calculated for a one-sided 0.050 exact Farrington-Manning test using StatXact 9®. If the P value from the exact Farrington-Manning test is <0.05 then the *Pre-Operative Biliary Drainage with SEMS* group will be considered non-inferior to the *No Pre-Operative Biliary Drainage* group. The expected probability of complications in the *Pre-Operative Biliary Drainage with SEMS* arm and *No Pre-Operative Biliary Drainage* arm is 37.2%, which was taken from the upper limit of the 95% CI from the meta-analysis described above and from the only available Level 1 study comparing no drainage to preoperative stenting in which the complication rate was reported to be 39% in the no drainage arm.[9] The non-inferiority margin (Δ) is 15%. Given these assumptions and a one-sided 5% significance level, 2 x 132 = 264 subjects will provide 80% power to reject the null hypothesis, that the *Pre-Operative Biliary Drainage with SEMS* group is inferior to the *No Pre-Operative Biliary Drainage* group.<br><br>To compensate for possible loss of subjects after enrollment and complete assessment of inclusion/exclusion criteria, an additional 10% of subjects will be enrolled, for a total of 2 x 147 = 294 subjects.

2 INTRODUCTION

This statistical plan addresses the planned analyses for the Preoperative Biliary Drainage RCT based on the protocol dated 20 October 2014, Version AD. All of the specified analyses may not be provided in reports to Competent Authorities but may be used for scientific presentations and/or manuscripts. The primary analysis will be based on the data through 120 days post-randomization or 30 days post-surgery whichever comes last.

3 ENDPOINT ANALYSIS

3.1 Primary Endpoint

The rate of serious adverse events is the primary endpoint for the study.

3.1.1 Hypotheses

Statistical testing will be performed to determine if the rate of complications for the Pre-Operative Biliary Drainage with SEMS group is non-inferior to the No Pre-Operative Biliary Drainage group. The null hypothesis is that the complication rate is inferior in the Pre-Operative Biliary Drainage with SEMS versus the No Pre-Operative Biliary Drainage group:

Ho: $\pi_{test} - \pi_{control} \geq \Delta$ (Inferior)

Ha: $\pi_{test} - \pi_{control} < \Delta$ (Non-inferior)

where π_{test} and $\pi_{control}$ are the probabilities of having pre-operative, operative and peri-operative complications in the Pre-Operative Biliary Drainage with SEMS arm and No Pre-Operative Biliary Drainage arm respectively, and Δ is defined as the non-inferiority margin.

3.1.2 Sample Size

A literature search of preoperative biliary drainage with self-expanding metal stents in patients with pancreatic or periampullary cancer yielded 8 articles with 305 patients.[21-24, 27-31]

The following meta-analysis of the probability for pre-operative, operative and post-operative complications was done:

- A rate of 24.2% [95% CI: 13.2%, 37.2%] was calculated using the 8 articles.

The sample size was calculated for a one-sided 0.050 exact Farrington-Manning test using StatXact 9®. If the P value from the exact Farrington-Manning test is <0.05 then the *Pre-Operative Biliary Drainage with SEMS* group will be considered non-inferior to the *No Pre-Operative Biliary Drainage* group. The expected probability of complications in the *Pre-Operative Biliary Drainage with SEMS* arm and *No Pre-Operative Biliary Drainage* arm is 37.2%, which was taken from the upper limit of the 95% CI from the meta-analysis described above and from the only available Level 1 study comparing no drainage to preoperative stenting in which the complication rate was reported to be 39% in the no drainage arm.[9] The non-inferiority margin (Δ) is 15%. Given these assumptions and a one-sided 5% significance level, 2 x 132 = 264 subjects will provide 80% power to reject the null hypothesis, that the *Pre-Operative Biliary Drainage with SEMS* group is inferior to the *No Pre-Operative Biliary Drainage* group.

To compensate for possible loss of subjects after enrollment and complete assessment of inclusion/exclusion criteria, an additional 10% of subjects will be enrolled, for a total of 2 x 147 = 294 subjects.

3.1.3 Statistical Methods

For calculating the rate of serious adverse events after randomization, patients with insufficient follow-up will be excluded from the calculation; that is, only patients that have at least follow-up through the long term visit (120/150 day follow-up) will be included unless the subject has already had a serious adverse event. All patients with a serious adverse will be counted in the analysis. Sensitivity analyses, such as a tipping point analysis will be

performed to assess the impact of the missing data, assuming extremes of either no SAEs or all SAEs for the primary endpoint, if the data support such analysis.

4 GENERAL STATISTICAL METHODS

4.1 Analysis Sets

Primary endpoint and selected secondary endpoints will be done for the following cohorts.

Enrolled Cohort

A subject is considered "enrolled" after signing the study-specific ICF. Subjects who sign the ICF but subsequently do not meet one or more of the eligibility criteria provided in the synopsis above will be considered screen failures and excluded from the study.

Intent-to-Treat Cohort (ITT)

This cohort consists of those "enrolled" subjects who meet all inclusion/exclusion criteria and are subsequently randomized.

Per-Protocol Cohort (PP)

The per-protocol cohort is a subset of the ITT subjects who are treated per protocol after randomization (Group 1 no stent, and Group 2 received a study stent) and who had no major protocol deviations (ICH E9 definitions).

5 ADDITIONAL DATA ANALYSES

5.1 Secondary Endpoints

1. Adverse events: rate, severity, seriousness, relatedness to stent or endoscopic or surgical procedure, impact on time of surgery, length of hospitalization and ICU stay
2. Time to surgery
3. Curative Intent Surgery details pertaining to intraoperative assessment of resectability, surgical resection and reconstruction techniques
4. Intraoperative blood loss and blood transfusions, duration of surgery
5. Biliary obstructive symptoms assessment (Week 2, surgery/transition to palliation, 30 day post-operative follow up and 120 day follow up)
6. Improvement of LFT levels as relative to baseline (Week 2, surgery/transition to palliation, 30 day post-operative follow up and 120 day follow up)
7. Stent placement success: ability to deploy the stent in satisfactory position across the stricture (Group 2)
8. Stent removal success: successful SEMS removal, either *en bloc* at time of surgery or endoscopically prior to surgery without stent removal related SAEs
9. Number, type, reason and timing of biliary re-interventions and time to first re-intervention.

10. Number and duration of hospital and ICU admissions

5.2 Baseline Data

Baseline data will be summarized. Subject demographics, clinical history, risk factors, LFTs, tumor diagnosis, and obstruction symptoms will be summarized using descriptive statistics (e.g., mean, standard deviation, n, minimum, maximum) for continuous variables and frequency statistics for discrete variables.

5.3 Post-Procedure Endpoints

Post-procedure information will be collected at regularly scheduled follow-up examinations as detailed in the clinical study event schedule and will be summarized using descriptive statistics for continuous variables (e.g., mean, standard deviation, n, minimum, maximum) and frequency tables or proportions for discrete variables.

5.4 Subgroup Analyses

The subgroup analyses will include tabulating the primary endpoint and select secondary endpoints by gender and the method of pancreatic anastomosis performed (PJ vs. PG).

5.5 Justification of Pooling

The analyses will be presented using pooled data across institutions. An analysis of the poolability will be made using logistic regression for binary outcomes, proportional hazards regression for time-to-event outcomes, or analysis of variance for continuous outcomes, to assess differences between study institutions and to justify pooling data across institutions.

5.6 Multivariable Analysis

Univariate and multivariate analyses will be performed to assess possible predictors of the rate of serious adverse events. Possible predictors may include any but not limited to demographic/baseline data and medical history data. Factors from the univariate model with $p \leq 0.20$ will also be modeled multivariately using a stepwise procedure in a generalized linear model or Cox proportional hazards regression model. The significance thresholds for entry and exit into the model will be set to $p<0.10$.

5.7 Analysis of LFT's and Obstruction symptoms

For analysis of LFT's and obstruction symptoms.The data will also be analyzed using a generalized linear model, including treatment group and baseline covariates as predictors. Interactions between time and treatment group will be explored. Other possible predictors may include any but not limited to demographic/baseline data and medical history data. Different correlation structures will be fit to determine the best model fit.

5.8 Analysis of Impact of Serious Adverse Events on Endpoints

For an analysis on the effect of the adverse events impact on time to surgery, length of hospitalization, and ICU stay, subjects with and without SAEs will be analyzed to determine if any differences occur. Appropriate testing will be done to determine this, i.e. a 2x2 ANOVA analysis with a treatment interaction.

5.9 Baseline Tumor Stage vs. Pathology Tumor Stage

A frequency table will be conducted to look for the differences between the baseline tumor stage and the pathology tumor stage diagnosis. This table will include all stages including a missing category for both time points.

5.10 Survival Analysis to 120 Days Post Randomization

An analysis of survival differences between the groups will be done to test if differences occur to 120 days post randomization. This will include Kaplan-Meir graphs with a Log-Rank Test to see if unadjusted differences occur. Also, a Cox proportional hazards regression model will be used. Possible predictors may include any but not limited to demographic/baseline data and medical history data. A step-wise procedure will be used to determine important predictors to stay in the model and the significance thresholds for entry and exit into the model will be set to $p<0.10$.

5.11 Changes to Planned Analyses

Any changes to the planned statistical analyses made prior to performing the analyses will be documented in a Statistical Analysis Plan approved prior to performing the analyses.

6 Validation

All clinical data reports generated per this plan will be validated per Global WI: Clinical Data Reporting Validation.

7 Programming Considerations

7.1 Statistical Software

All statistical analyses will be done using The SAS System software, version 8 or higher (Copyright © 2000 SAS Institute Inc., SAS Campus Drive, Cary, North Carolina 27513, USA. All rights reserved). For the primary endpoint analysis, StatXact 9® software can be used since SAS does not provide exact non-inferiority analysis as of the writing of this SAP.

7.2 Format of output

Results of analysis will be output programmatically to Word documents from SAS with no manual intervention. All output for the final statistical report will be in the form of a Word document containing tables, figures, graphs, and listings, as appropriate.

7.3 Rules and Definitions

Binary event rates (proportions) will be reported on a per patient basis.

The last follow-up date will be the latest of the following dates for each patient: date of an adverse event, index procedure date, follow-up visit date, any stent procedure/reintervention date, surgery date, stent removal date, and device event date.

Serious Adverse Event will be defined as an adverse event that:

- Led to death
- Led to a serious deterioration in the health of the subject that either resulted in:
  - a life-threatening illness or injury, or
  - a permanent impairment of a body structure or a body function, or
  - in-patient hospitalization or prolonged hospitalization (of an existing hospitalization), or
  - medical or surgical intervention to prevent life-threatening illness or injury or permanent impairment to a body structure or a body function
- Led to fetal distress, fetal death, or a congenital abnormality or birth defect.

Note: Planned hospitalization for a pre-existing condition, or a procedure required by the protocol, without serious deterioration in health, is not considered a serious adverse event.

Definitions of complication criteria (per van der Gaag article):

Specific PBD (ERCP, PTC) related:

- **Acute pancreatitis** Abdominal pain and a serum concentration of pancreatic enzymes (amylase or lipase) three or more times the upper limit of normal, that required more than one night of hospitalization
- **Acute cholecystitis** No suggestive clinical or radiographic signs of acute cholecystitis before the procedure and if emergency cholecystectomy is subsequently required
- **Perforation** Retroperitoneal or bowel-wall perforation documented by any radiographic technique or direct visual evidence
- **Stent Occlusion** Recurring obstructive jaundice with necessary stent replacement

Specific surgery related:

- **Pancreaticojejunostomy leakage** Drain output of any measurable volume of fluid on or after postoperative day 3 with an amylase content greater than 3 times the serum amylase activity, graded according to clinical course (ISGPS grade A, B, C), or direct visual evidence of defect at anastomosis
- **Delayed gastric emptying** Gastric stasis requiring nasogastric intubation for 10 days or more, or the inability to tolerate a regular (solid) diet on or before the fourteenth postoperative day, not due to sequelae of intra-abdominal complications (i.e. abscess, anastomotic leakage)
- **Biliary leakage** Bilirubin in abdominal drain or dehiscence found at laparotomy
- **Gastro/-duodenojejunostomy leakage** Conclusive radiographic or direct visual evidence of a defect of the anastomosis
- **Intra-abdominal abscess formation** Intra-abdominal fluid collection with positive cultures identified by ultrasonography or computed tomography, associated with persistent fever and elevations of white blood cells
- **Wound infection** Requiring intervention otherwise considered as minor complication
- **Portal Vein Thrombosis** Conclusive radiologic evidence of thrombosis

Following either procedure:

- **Cholangitis** Elevation in temperature more than 38°C, thought to have a biliary cause, without concomitant evidence of acute cholecystitis, requiring intervention
- **Hemorrhage** Bleeding after the index procedure requiring transfusion of $\geq$4 units of packed cells within a 24-hour period, or leading to relaparotomy/intervention
- **(Emergency) (re)laparotomy** Any (other) reason following either preoperative biliary drainage or another surgical procedure
- **Pneumonia** Pulmonary infection with radiological confirmation and requiring antibiotic treatment
- **Mortality** In-hospital death, due to protocol complications or any cause, including progression of disease, within the study period
